# Dosage-sensitive regulation of Cofilin-1 and LIMK signaling controls myoblast differentiation and fusion

**DOI:** 10.1007/s00018-026-06437-1

**Published:** 2026-09-18

**Authors:** Dora Gjirlić, Anja Weber, Guido Posern, Anurag Kumar Singh

**Affiliations:** Institute for Physiological Chemistry, Medical Faculty, Martin Luther University Halle- Wittenberg, 06114 Halle (Saale), Germany

**Keywords:** Myogenesis, Cofilin-1, Cofilin-2, Actin cytoskeleton, LIM Kinase, MRTF signaling, Myoblast differentiation, Cell fusion, Cytoskeletal regulation

## Abstract

Skeletal muscle differentiation requires coordinated cytoskeletal remodeling and transcriptional reprogramming. Members of the actin-depolymerizing factor/Cofilin family regulate actin filament turnover, yet the isoform-specific functions of Cofilin-1 and Cofilin-2 during myogenesis remain incompletely understood. Murine C2C12 myoblasts were used to investigate the expression and function of Cofilin isoforms during differentiation. CRISPR/Cas9-mediated knockout, shRNA-mediated knockdown, immunofluorescence, gene expression analyses, MRTF reporter assays, and pharmacological inhibition of LIM kinase were used to assess myogenic progression and signaling. Myogenic differentiation was accompanied by a pronounced isoform transition characterized by progressive downregulation of Cofilin-1 and Actin-Depolymerizing Factor together with increased expression of Cofilin-2. Loss of Cofilin-1 resulted in marked morphological abnormalities, impaired cell-cycle exit, elevated MRTF activity, and reduced myotube formation, whereas Cofilin-2 deficiency produced comparatively mild effects during early differentiation. Importantly, partial reduction of Cofilin-1 enhanced myoblast fusion, while stronger depletion impaired differentiation, demonstrating a dosage-sensitive requirement for Cofilin-1. In addition, Cofilin-1 activity was dynamically regulated by transient LIM kinase-mediated phosphorylation during early differentiation, and inhibition of this pathway disrupted myogenic progression. Analyses of mRNA and protein stability indicated that isoform switching is primarily regulated at the level of gene expression. Cofilin-1 functions as a dosage-sensitive regulator of myogenic progression that links cytoskeletal remodeling to signaling pathways controlling proliferation and differentiation. Coordinated regulation of Cofilin-1 expression and activity, together with the transition to Cofilin-2, is required for efficient myoblast fusion and muscle formation.

**Supplementary Information:**

The online version contains supplementary material available at https://doi.org/10.1007/s00018-026-06437-1.

## Introduction

Myogenesis is a tightly regulated developmental program governed by the myogenic regulatory factor (MRF) family. Differentiation of myoblasts to myotubes is accompanied by cell cycle arrest and the progressive accumulation of contractile proteins, including myosin heavy chain (MHC), during the later stages of differentiation [1, 2]. Commitment of progenitor cells to myoblasts is initiated by MyoD and followed by the activation of Myogenin (MyoG) and other MRFs [3, 4]. During fusion, myoblasts first migrate and establish close proximity through lamellipodia and filopodia, recognize one another, elongate, and align laterally before membrane merger occurs [1]. Of the two fusing cells, one develops a unilateral actin layer that serves as a scaffold, while the adjacent cell generates invasive membrane protrusions enriched in branched F-actin, thereby forming a fusogenic synapse [5–8]. This process is driven by MyoD-dependent fusogenic factors, including Myomaker and Myomixer/Myomerger (MymK and MymX), as well as Caveolin-3 (Cav3), a scaffolding protein essential for caveolar membrane formation and membrane remodeling [3, 9–12].

Actin remodeling is regulated by multiple protein families, among which the Actin Depolymerizing Factor (ADF)/Cofilin family-comprising Cofilin-1, Cofilin-2, and ADF/Destrin-plays a central role. These small (~ 19 kDa) proteins bind F-actin and form co-filaments that induce torsional undertwisting. While historically thought to sever filaments independently, recent structural evidence demonstrates that Cofilin acts as a cooperative platform, priming the filament for rapid severing and depolymerization by associated factors like AIP1 and Coronin to maintain the cellular pool of available G-actin [13–15]. Cofilin-1 and Cofilin-2 share approximately 80% sequence identity and are ~ 70% identical to ADF in mouse [16]. Cofilin activity is regulated through reversible phosphorylation at Ser3, primarily mediated by LIM kinases 1 and 2 (LIMK1/2) downstream of the Rac1, Cdc42, and RhoA-ROCK signaling pathways [17, 18]. Phosphorylation at Ser3 inactivates Cofilin and stabilizes F-actin structures, such as the actin scaffold at the fusogenic synapse [5, 19]. Interestingly, the biochemical and functional properties vary between isoforms, suggesting that the Cofilin-1 to Cofilin-2 transition represents a shift in how the local actin cytoskeleton responds to upstream signaling cascades [16].

Although ADF/Cofilin isoforms were long considered functionally redundant, growing evidence indicates that they possess distinct properties tailored to specific developmental contexts [16, 20]. Cofilin activity, actively promotes the assembly of contractile actin bundles and stress fibers by accelerating filament turnover, generating new barbed ends for rapid polymerization [21], and balancing myosin-II binding [22]. Consequently, these proteins have emerged as central nodes that integrate local cytoskeletal architecture with broader tissue homeostasis and complex developmental programs [20, 23]. Beyond structural remodeling, Cofilin-mediated actin dynamics provide a direct regulatory link to myogenesis through the mechanosensitive Myocardin-Related Transcription Factor/Serum Response Factor (MRTF/SRF) signaling axis. MRTF-A and MRTF-B are mechanosensitive co-activators that regulate the balance between myogenic proliferation and differentiation, with MRTF-A levels progressively decreasing as myogenesis proceeds [24, 25]. Mechanistically, the MRTF/SRF transcriptional activity depends directly on monomeric G-actin abundance, which is tightly modulated by Cofilin-mediated depolymerization. Under basal conditions, the binding of monomeric G-actin physically retains MRTFs in the cytoplasm, whereas a reduction in free G-actin availability-driven by altered actin turnover-promotes their nuclear translocation and subsequent transcriptional activation of downstream myogenic programs [26].

However, despite the well-documented tissue-specific distribution of Cofilin isoforms and emerging evidence for their non-equivalence, it remains unclear how these differences are functionally exploited during myogenesis. Specifically, whether Cofilin-1 levels must be precisely regulated during myoblast differentiation and fusion, and how the developmental transition from Cofilin-1 to Cofilin-2 is coordinated, remain largely unresolved. Therefore, we systematically examined the expression dynamics and functional contributions of Cofilin-1, Cofilin-2, and ADF in C2C12 myoblasts undergoing myogenic differentiation. Using a combination of CRISPR/Cas9-mediated knockout, shRNA knockdown approaches, and pharmacological perturbation of LIM kinase signaling, we investigated how modulation of these actin regulatory proteins influences the transition from proliferating myoblasts to differentiated myotubes. Our analyses reveal a pronounced isoform transition characterized by progressive downregulation of Cofilin-1 and ADF together with increased expression of the muscle-specific isoform Cofilin-2. Functionally, we identify Cofilin-1 as a key regulator of early myogenic progression, whose activity is controlled through both dosage-dependent mechanisms and dynamic phosphorylation, thereby governing skeletal muscle differentiation.

## Materials and methods

### Cell culture

C2C12 myoblasts (DSMZ - German Collection of Microorganisms and Cell Cultures) were maintained in Dulbecco’s Modified Eagle Medium (DMEM) supplemented with 10% fetal bovine serum (FBS), 2 mM L-glutamine, 1 mM sodium pyruvate, and antibiotic-antimycotic (Thermo Fisher Scientific) at 37 °C and 5% CO₂. Cells were cultured up to approximately 60% confluency before passaging. For differentiation, C2C12 myoblasts were seeded at high density to achieve near confluency. The following day (designated day 0 of differentiation), growth medium was replaced with differentiation medium supplemented with 2% horse serum (HS). Transient inhibition of LIMK activity was achieved by treating the cells with 5 µM BMS-5 (LIMKi3; HY-18305, MedChemExpress) exclusively from day 0 to day 1 of differentiation. On the following day, the inhibitor-containing medium was removed, and the cells were washed and subsequently maintained in standard differentiation medium until day 6.

### Generation of knockout (KO) and knockdown (KD) cell lines

C2C12 cell lines with modified expression profiles were generated using lentiviral delivery systems. For all constructs, functional lentiviral particles were produced by co-transfecting the respective transfer vector with the VSV-G envelope plasmid pMD2.G (Addgene plasmid #12259) and the packaging plasmid psPAX2 (Addgene plasmid #12260; both kindly provided by Didier Trono).

For CRISPR/Cas9-mediated gene knockouts, two independent guide RNAs (sgRNAs) per target gene were designed using the online tool CHOPCHOP (https://chopchop.cbu.uib.no/) based on predicted high on-target efficiency and minimal off-target potential (Supplementary Table S1). These sgRNA sequences were cloned into lentiCRISPRv2 (Addgene plasmid #52961, a gift from Feng Zhang). Following lentiviral infection, stable pools for Cofilin-1, Cofilin-2, and ADF/Destrin were obtained via one week of puromycin selection, and knockout efficiency was validated at the protein level by Western blotting. Due to severely impaired differentiation capacity, single-cell clonal selection was not pursued.

For stable RNA interference-mediated knockdowns, shRNA constructs were designed using the siRNA Wizard tool (InvivoGen). After initially testing three candidate shRNAs for Cofilin-1 and Cofilin-2, and two for ADF, the most efficient sequence for each target was selected via Western blotting. These sequences were cloned into a modified pLVX-shRNA2-Crimson-puro vector (Takara Bio Inc.), in which the native ZsGreen cassette was replaced by a PGK promoter-driven E2-Crimson cassette fused to a puromycin resistance gene via an EMCV IRES.

While standard stable knockdowns were selected with puromycin, fine-tuned expression titrations were achieved by applying these lentiviral shRNA constructs transiently in a dose-dependent manner. For these titration experiments, C2C12 cells were seeded in 12-well plates and transduced overnight with either low or high virus-containing supernatant (0.5 mL versus 4.0 mL per well) to modulate the extent of knockdown. Following transduction, cells were directly switched to differentiation medium to allow myogenic progression and collected at the indicated time points for downstream analysis.

### Immunoblotting

Western blot analysis was performed using standard protocols. Cells were lysed in RIPA buffer (0.1% SDS, 0.5% sodium deoxycholate, 1% Triton X-100, 1 mM EDTA, 150 mM NaCl, and 50 mM Tris, pH 7.4) supplemented with protease and phosphatase inhibitors (1 mM PMSF, 10 µg/mL aprotinin, and 2 mM sodium orthovanadate). Protein concentrations were determined using the Pierce BCA Protein Assay Kit (#23227; Thermo Scientific) according to the manufacturer’s instructions prior to SDS-PAGE. The primary antibodies used were: anti-Cofilin-1 (5175 S; Cell Signaling Technology; 1:1,000), anti-Cofilin-2 (MA5-36233; Invitrogen; 1:1,000), anti-ADF/Destrin (67657-1-Ig; Proteintech; 1:1,500), anti-phospho-Cofilin (Ser3) (3313 S; Cell Signaling Technology; 1:1,000), anti-Tubulin (T9026; Sigma-Aldrich; 1:2,000), anti-FLAG (F7425; Sigma-Aldrich; 1:1,000), anti-CAV3 (sc-5310; Santa Cruz Biotechnology; 1:1,000), anti-Myosin Heavy Chain 1/2/4/6 (sc-32732; Santa Cruz Biotechnology; 1:1,000), anti-Myogenin (sc-12732; Santa Cruz Biotechnology; 1:1,000), anti-MyoD (MA1-41017; Invitrogen; 1:1,000), anti-Phospho-Histone H3 (Ser10) (53348 S; Cell Signaling Technology; 1:1,000), and anti-RhoB (63876 S; Cell Signaling Technology; 1:500). Primary antibodies were incubated overnight at 4 °C. Horseradish peroxidase (HRP)-conjugated secondary antibodies (1:5,000; Thermo Fisher Scientific) were applied for 1 h at room temperature. Protein bands were detected via chemiluminescence using a Bio-Rad ChemiDoc MP Imaging System. Densitometric quantification was performed using ImageJ/Fiji software.

### Reverse transcription-quantitative PCR (RT-qPCR)

Total RNA was extracted using the NucleoSpin RNA Mini Kit (Macherey-Nagel Inc.) according to the manufacturer’s instructions. cDNA synthesis was performed using the Verso cDNA Synthesis Kit (Thermo Fisher Scientific). Quantitative PCR was performed using a LightCycler 480 system (Roche) with Dynamo ColorFlash SYBR Green chemistry. Primer sequences are listed in Supplementary Table S1. Expression levels were normalized to the geometric mean of two independent housekeeping genes. Relative quantification was calculated using either the ΔCt or ΔΔCt method (Pfaffl, 2001), depending on experimental design. Statistical analysis was performed using one-way ANOVA and Student’s t-test.

### Staining and immunofluorescence microscopy

To assess individual cell morphology, cells were seeded at a density of 5 × 10⁴ cells (for Day 0 morphological visualization) or 3 × 10⁵ cells (for differentiation time points) in 35 mm culture dishes. Following the indicated experimental timelines, cells were fixed with 3.7% paraformaldehyde (PFA) for 10 min, stained with 0.5% crystal violet (dissolved in methanol) for 15 min, rinsed thoroughly with water, and air-dried prior to imaging. For immunofluorescence analysis, cells were seeded onto coverslips in 12-well plates at a density of either 5 × 10⁴ or 2 × 10⁵ cells. Samples were collected at days 0, 4, and 6 of differentiation. Cells were fixed in 3.7% PFA for 15 min, washed in 50 mM glycerol-containing wash buffer, permeabilized with 0.2% Triton X-100 for 10 min, and blocked in blocking solution (10% FBS, 1% BSA, 0.05% Triton X-100) for 30 min. Primary antibodies-including Cofilin-1, Cofilin-2, ADF/Destrin, in-house MRTF-A, and MYH (dilutions 1:100–1:200), alongside Alexa Fluor™ 488 Phalloidin (Thermo Fisher Scientific; 1:200)-were prepared in blocking solution. Coverslips were incubated face-down on 45 µL droplets of the primary antibody solution for 1 h. Following three washes, secondary antibodies (1:200; anti-mouse or anti-rabbit) and DAPI (1:5000) were applied for 2 h. Coverslips were washed and mounted prior to imaging with a Zeiss Axio Observer Z1 inverted fluorescence microscope. For fusion index analysis, at least 10 random fields per sample were captured at 20× magnification. The fusion index was calculated as: (number of nuclei within MYH-positive myotubes containing ≥ 3 nuclei / total number of nuclei) × 100.

### MRTF reporter assay

MRTF-dependent transcriptional activity was quantified using the Dual-Glo^®^ Luciferase Assay System (Promega). Briefly, freshly selected knockdown C2C12 cells were seeded in 12-well plates at a density of 8 × 10⁴ cells per well. Cells were co-transfected using polyethylenimine (PEI) with a total of 1 µg DNA, consisting of 250 ng p3DA-Luc (MRTF/SRF-responsive reporter; [27, 28]), 50 ng pRL-TK (Renilla luciferase internal control), and 700 ng pLVX-puro as carrier DNA. At 30–35 h post-transfection, cells were harvested and lysed according to the manufacturer’s protocol. Firefly and Renilla luciferase activities were measured using a Promega GloMax^®^ Plate Reader. Relative luciferase activity was calculated by normalizing firefly luminescence to the internal Renilla control.

### mRNA and protein stability assays

To assess transcript and protein stability, C2C12 cells were seeded at a density of 3 × 10⁵ (6 well plate) or 7 × 10⁵ (60 mm dish) cells and treated either at the myoblast stage (24 h after seeding) or at the differentiated myotube stage (day 6). For inhibition of protein synthesis, cells were treated with 75 µg/mL Cycloheximide (Cat. No. #01810-1G, Sigma). For inhibition of transcription, cells were treated with 5 µg/mL Actinomycin D (Cat. No. #1229, Tocris). Samples were collected at defined time points following treatment (as indicated in the results section), and RNA or protein levels were assessed as described above.

### Cell viability and metabolic activity assay 

Cell viability and metabolic activity were evaluated using the XTT Cell Viability Kit (#9095S; Cell Signaling Technology) according to the manufacturer’s instructions. Briefly, C2C12 myoblasts were cultured and treated with LIMKi3 (5 µM) or vehicle control (DMSO) in 96-well plates. At the indicated time points (days 1, 3, and 6), XTT detection solution (prepared by mixing XTT reagent with the Electron Coupling Solution at a 50:1 ratio) was added directly to the culture media. Cells were incubated at 37 °C for 2 h. Absorbance was subsequently measured at 450 nm using a microplate spectrophotometer, with specific absorbance calculated by subtracting background absorbance at 650 nm. Data were expressed as a percentage relative to vehicle-treated control cells.

## Results

### ADF/Cofilin family members display distinct expression dynamics during myogenic differentiation

The ADF/Cofilin family consists of three closely related actin-binding proteins-Cofilin-1, Cofilin-2, and actin depolymerizing factor (ADF/Destrin)-that share high sequence similarity and function as central regulators of actin filament turnover. Given the extensive cytoskeletal remodeling required during myogenic differentiation, we assessed whether these family members exhibit distinct expression patterns throughout this process.

**Fig. 1:**
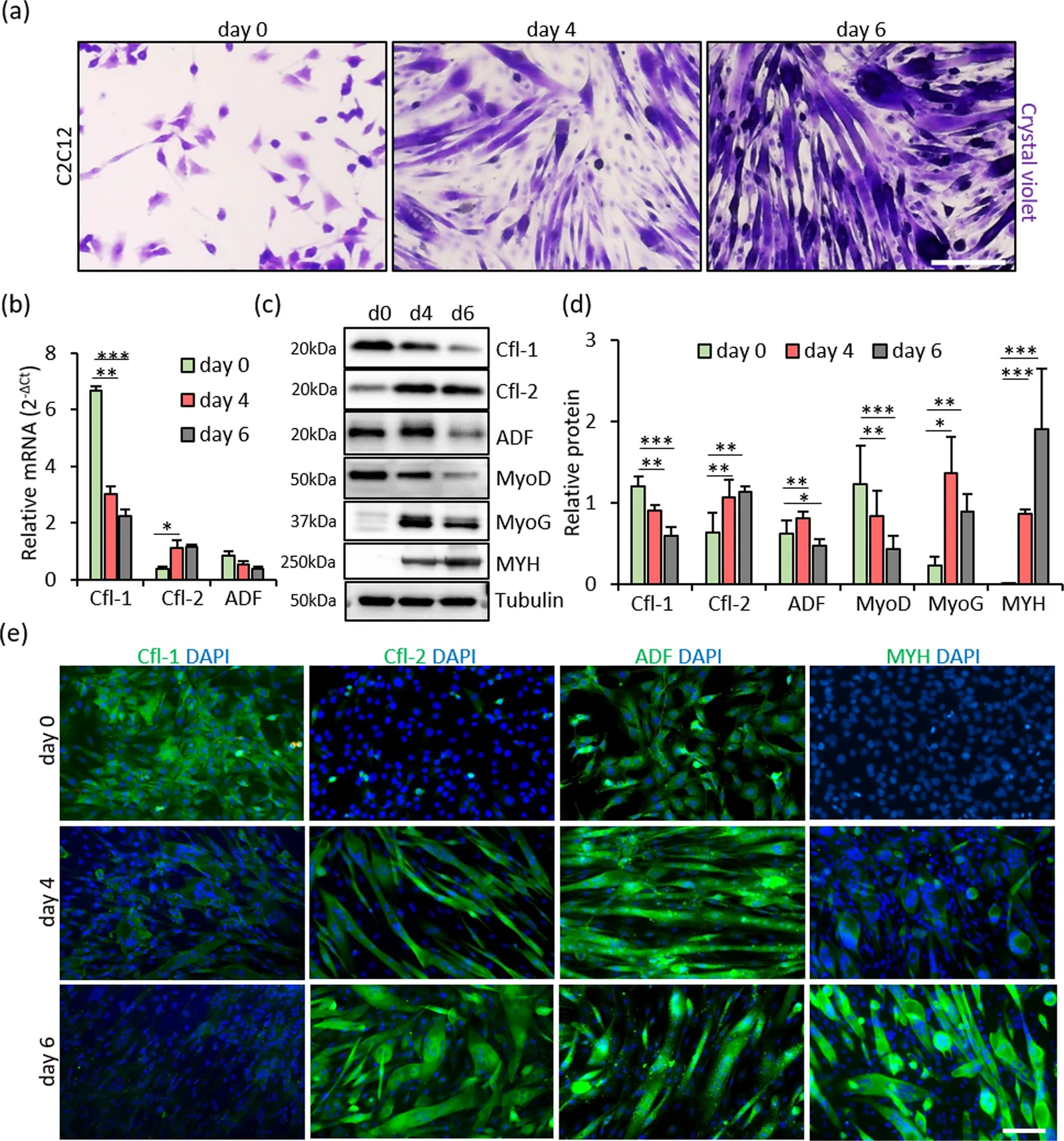
Distinct expression patterns of ADF/Cofilin family members during C2C12 myogenic differentiation. (**a**) Crystal violet staining of C2C12 cultures at differentiation days 0, 4, and 6. For Day 0, 5 × 10^4^ cells were seeded to visualize distinct single-cell morphological features, whereas differentiation time points (Days 4 and 6) were seeded at 3 × 10^5^ cells to achieve confluence prior to induction of differentiation. (**b**) Relative mRNA expression of Cofilin-1 (Cfl-1), Cofilin-2 (Cfl-2), and ADF/Destrin during differentiation (days 0, 4, and 6), quantified by RT-qPCR and normalized to the geometric mean of GAPDH and HPRT. Error bars, SD (*n* = 3), one-way ANOVA (**c**) Immunoblots of the myogenic marker proteins MyoD, Myogenin (MyoG), and Myosin Heavy Chain (MYH), together with ADF/Cofilin family members. (**d**) Quantification of immunoblots normalized to Tubulin control. Error bars, SEM (*n* = 3), Student’s t-test (**e**) Representative immunofluorescence images showing Cofilin-1, Cofilin-2, and ADF at days 0, 4, and 6 of differentiation. *p < 0.05, **p < 0.01, ***p < 0.001. Scale bar: 100 μm

Murine C2C12 myoblasts were induced to differentiate by switching from growth medium to differentiation medium. Morphological progression from proliferating myoblasts (day 0) to nascent multinucleated myotubes was evident by days 4 and 6, as visualized by crystal violet staining (Fig. 1a). Cofilin-1 mRNA was highly expressed in proliferating myoblasts (day 0) and progressively decreased during differentiation (Fig. 1b). A similar downward trend was observed for ADF, although its expression levels were substantially lower than those of Cofilin-1 at all examined time points. In contrast, Cofilin-2 expression increased steadily during differentiation, reaching its highest level at day 6.

We next examined whether these transcriptional changes were reflected at the protein level. Western blot analysis confirmed that protein expression followed the corresponding mRNA patterns (Fig. 1c). Protein levels of Cofilin-1 and ADF declined significantly as differentiation progressed, whereas Cofilin-2 protein accumulated over time. In parallel, differentiation was validated by the expected temporal regulation of myogenic markers, with MyoD and Myogenin (MyoG) levels shifting alongside a robust accumulation of Myosin Heavy Chain (MYH) isoforms observed at later stages (Fig. 1c, d).

Consistent with the biochemical data, Cofilin-1 immunofluorescence was prominent in myoblasts and diminished during myotube formation, whereas Cofilin-2 staining increased in differentiated cells (Fig. 1e). The accumulation of MYH further verified successful differentiation and myotube maturation. These results demonstrate that members of the ADF/Cofilin family are differentially and reciprocally regulated during C2C12 myogenic differentiation.

### Cofilin-1 is required for efficient myogenic differentiation and myoblast fusion

**Fig. 2:**
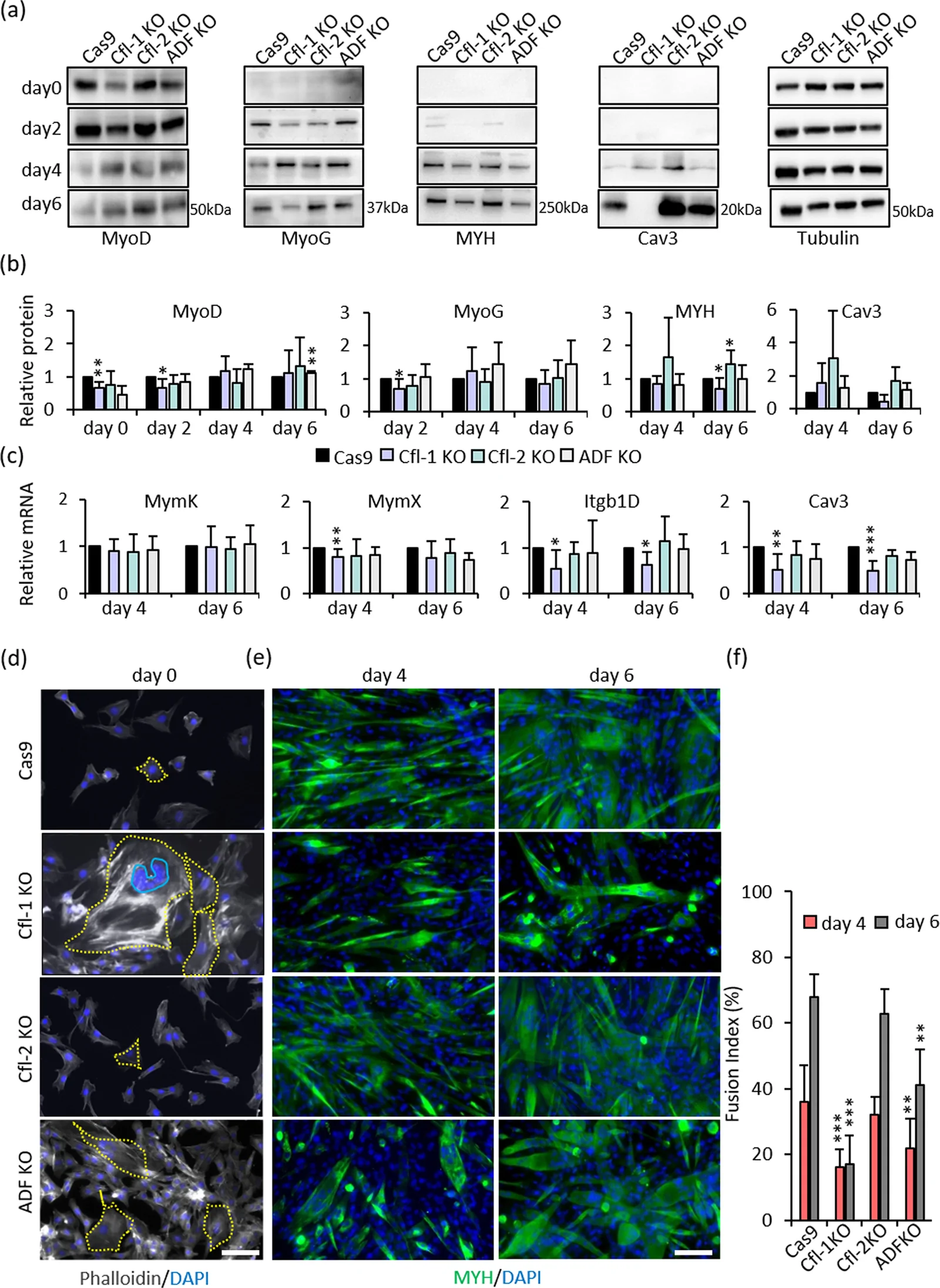
Cofilin-1 knockout impairs differentiation marker expression, profusion gene expression, and myoblast fusion. C2C12 cells with stable CRISPR/Cas9-mediated knockout (KO) of the indicated genes were subjected to myogenic differentiation for 6 days. (**a**) Representative immunoblots of differentiation markers MyoD, Myogenin (MyoG), Myosin Heavy Chain (MYH), and Caveolin-3 (Cav3). (**b**) Quantification of immunoblots relative to Tubulin and normalized to Cas9 control. (**c**) Relative mRNA expression of fusion-related genes Myomaker (Mymk), Myomixer (Mymx), integrin β1D (Itgb1D), and Caveolin-3 (Cav3), determined by RT-qPCR. Values are normalized to housekeeping genes and to Cas9 control. (**d**) Representative fluorescence images of phalloidin-stained cells showing F-actin in Cas9 control and Cofilin KOs myoblasts at differentiation day 0. Yellow line indicates cell border, blue line indicates nuclear shape and size. (**e**) Representative immunofluorescence images of Cas9 control and Cofilin KO cultures at differentiation days 4 and 6. (**f**) Quantification of the fusion index calculated from MYH-positive myotubes at differentiation days 4 and 6. A total of 10 fields from two independent experiments were analyzed for each condition. Error bars, SEM (*n* = 5) for (b), (*n *= 3) for (c) and SD (*n* = 10) for (g). *p < 0.05, **p < 0.01, ***p < 0.001. Scale bars: 100 μm

To directly assess the functional contribution of Cofilin isoforms to myogenic differentiation, C2C12 knockout (KO) cell lines for Cofilin-1, Cofilin-2, and ADF were generated using CRISPR/Cas9 and validated for efficient depletion of the respective proteins (Fig. S1a). Western blot analysis revealed that the loss of Cofilin-1 severely impaired the expression of key myogenic differentiation markers (Fig. 2a, b). In Cofilin-1 KO cells, protein levels of the early myogenic regulators MyoD and MyoG were reduced at days 0 and 2 of differentiation, while the accumulation of the late differentiation marker MYH was markedly diminished at day 6. In contrast, Cofilin-2 KO cells did not exhibit reduced MyoD or MyoG expression, instead showing increased MYH protein levels and an upward trend in the expression of the pro-fusion associated protein Caveolin-3 compared to Cas9 control cells (Fig. 2b).

Relative expression levels of the fusion-related transcripts Myomaker (MymK), Myomixer (MymX), the splice variant Integrin ß1D (Itgb1D), and Caveolin-3 (Cav3) were calculated as double-relative values (ΔΔCt) normalized to housekeeping genes and Cas9 control cells. Cofilin-1 KO cells displayed a significant reduction in MymX, Itgb1D, and Cav3 transcripts (Fig. 2c), consistent with the observed defects in differentiation and fusion. Conversely, Cofilin-2 KO cells maintained expression levels of pro-fusion transcripts comparable to Cas9 control cells, indicating that the transcriptional activation of the myogenic program remains intact in the absence of this isoform.

In addition to biochemical alterations, Cofilin-1 KO cells exhibited pronounced morphological abnormalities. Phalloidin staining of F-actin revealed a subpopulation of enlarged, frequently polynucleated cells with irregularly shaped nuclei and prominent actin stress fibers within the Cofilin-1 KO group. Similar but less pronounced phenotypic features were observed in ADF KO cells (Fig. 2d, S1b, c). The presence of these prominent stress fibers is indicative of impaired actin-severing activity.

Immunofluorescence analysis further revealed reduced MYH accumulation in Cofilin-1 KO cells at days 4 and 6 of differentiation compared to controls (Fig. 2e). Quantification of the fusion index by analyzing MYH-positive myotubes confirmed a significant reduction in fusion efficiency in Cofilin-1 KO cells at both time points. In contrast, Cofilin-2 KO cells did not display a detectable fusion deficit, while ADF KO cells exhibited a milder defect than that observed in the Cofilin-1 KO cells (Fig. 2f).

Taken together, these results demonstrate that Cofilin-1, but not Cofilin-2, is required for efficient myogenic differentiation and myoblast fusion. This is highly consistent with its elevated baseline expression in myoblasts and during the early stages of differentiation as shown in Fig. 1.

### Cofilin-1 knockdown confirms differentiation and fusion defects observed in knockout cells

**Fig. 3:**
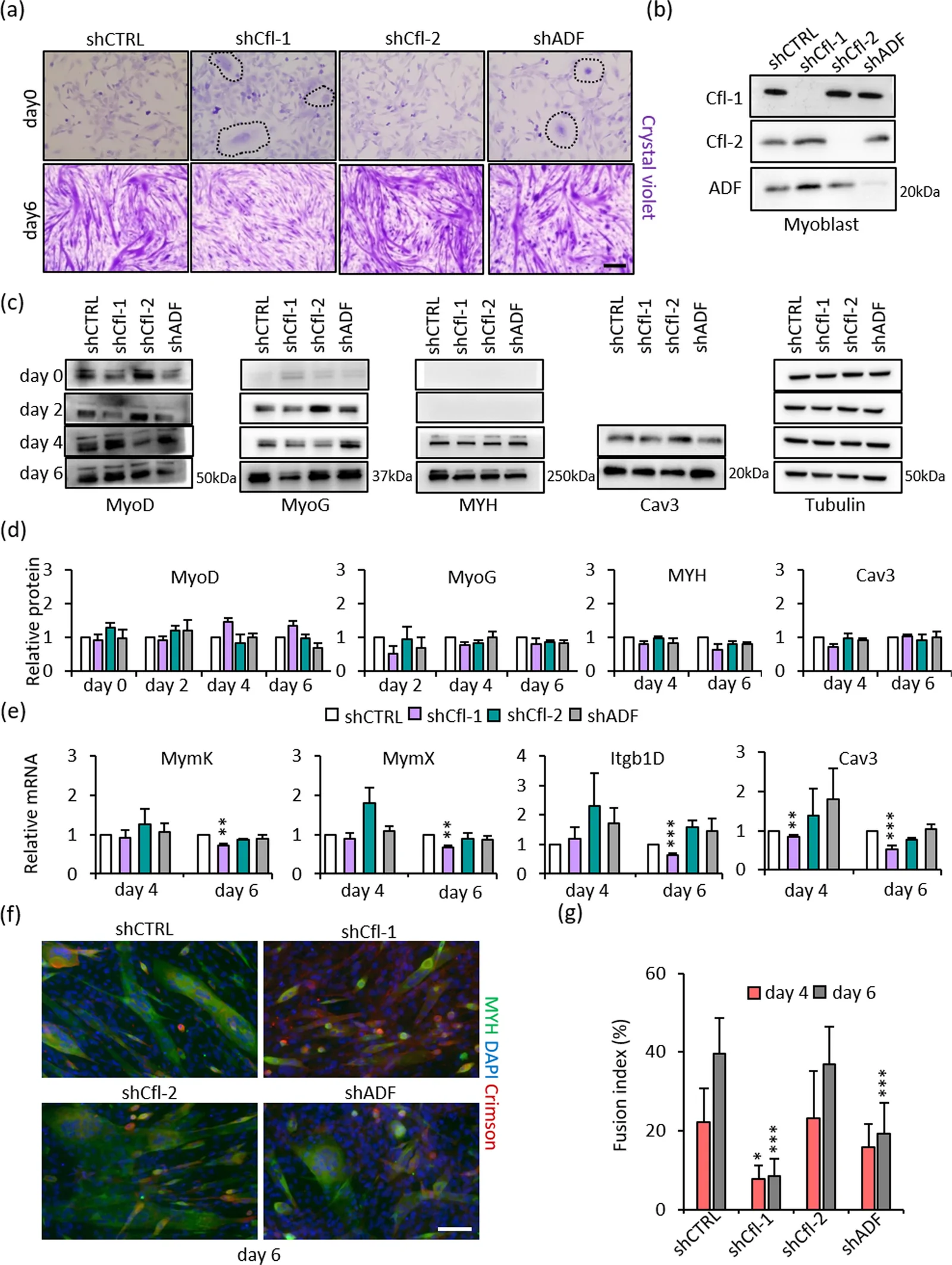
Cofilin-1 knockdown results in milder but consistent defects in myogenic differentiation and fusion. C2C12 cells with stable shRNA-mediated knockdown of the indicated genes were subjected to myogenic differentiation for 6 days. (**a**) Crystal violet staining at the myoblast stage (day 0) and after differentiation (day 6). (**b**) Representative immunoblots of the targeted Cofilin isoforms. (**c**) Immunoblots of myogenic differentiation markers MyoD, Myogenin (MyoG), Myosin Heavy Chain (MYH), and Caveolin-3 (Cav3). (**d**) Quantification of immunoblots relative to Tubulin and normalized to shCTRL. (**e**) Relative mRNA expression of fusion-related genes Myomaker (Mymk), Myomixer (Mymx), integrin β1D (Itgb1D), and Caveolin-3 (Cav3) determined by RT-qPCR. Values are normalized to housekeeping genes and to shCTRL. (**f**) Representative immunofluorescence images showing MYH-positive myotubes at day 6 of differentiation. (**g**) Fusion index calculated from MYH-positive myotubes at day 6 of differentiation. A total of 10 fields from two independent biological repeats were analyzed for each condition. Error bars, SEM (*n* = 3) for (d, e) and SD (*n* = 10) for (g). Statistical significance was determined using Student’s t-test (*p < 0.05, **p < 0.01, ***p < 0.001). Scale bars: 200 μm (a) and 100 μm (f)

To determine whether the phenotypes observed in Cofilin knockout cells could be reproduced using an independent loss-of-function approach, we employed shRNA-mediated knockdown of individual Cofilin isoforms. Crystal violet staining revealed that knockdown cultures retained morphological abnormalities, although these were less pronounced than those observed in the KO cell lines (Fig. 3a). At the myoblast stage (day 0), both shCofilin-1 and shADF populations contained a distinct fraction of enlarged, polynucleated cells. By day 6 of differentiation, shCofilin-1 cultures exhibited a visible reduction in myotube formation compared to control cells, whereas shADF cells showed only a mild impairment (Fig. 3a). Knockdown efficiency was validated at the protein level via Western blot analysis (Fig. 3b).

Analysis of differentiation markers revealed modest alterations in shCofilin-1 cells (Fig. 3c, d). Although these changes were generally small and did not reach statistical significance at any single time point, the overall temporal trend was consistent with a partial impairment of differentiation. RT-qPCR analysis confirmed that the core fusion program was disrupted in shCofilin-1 cells, showing a significant downregulation of key transcripts including Mymk, Mymx, Itgb1D, and Cav3 at day 6 of differentiation compared to shCTRL cells (Fig. 3e). Immunofluorescence staining further revealed reduced MYH accumulation and fewer mature myotubes in shCofilin-1 and shADF knockdown cells (Fig. 3f). Quantitative assessment confirmed a significant reduction in the fusion index of shCofilin-1 cells relative to shCTRL and shCofilin-2 lines (Fig. 3g). Notably, the relative severity of fusion defects across the various cell lines was strictly preserved between the KO and KD conditions, demonstrating that the overall phenotypic trends remain robust and consistent across both experimental genetic approaches.

### Cofilin-1 deficiency enhances MRTF activity and alters cell cycle exit dynamics

**Fig. 4:**
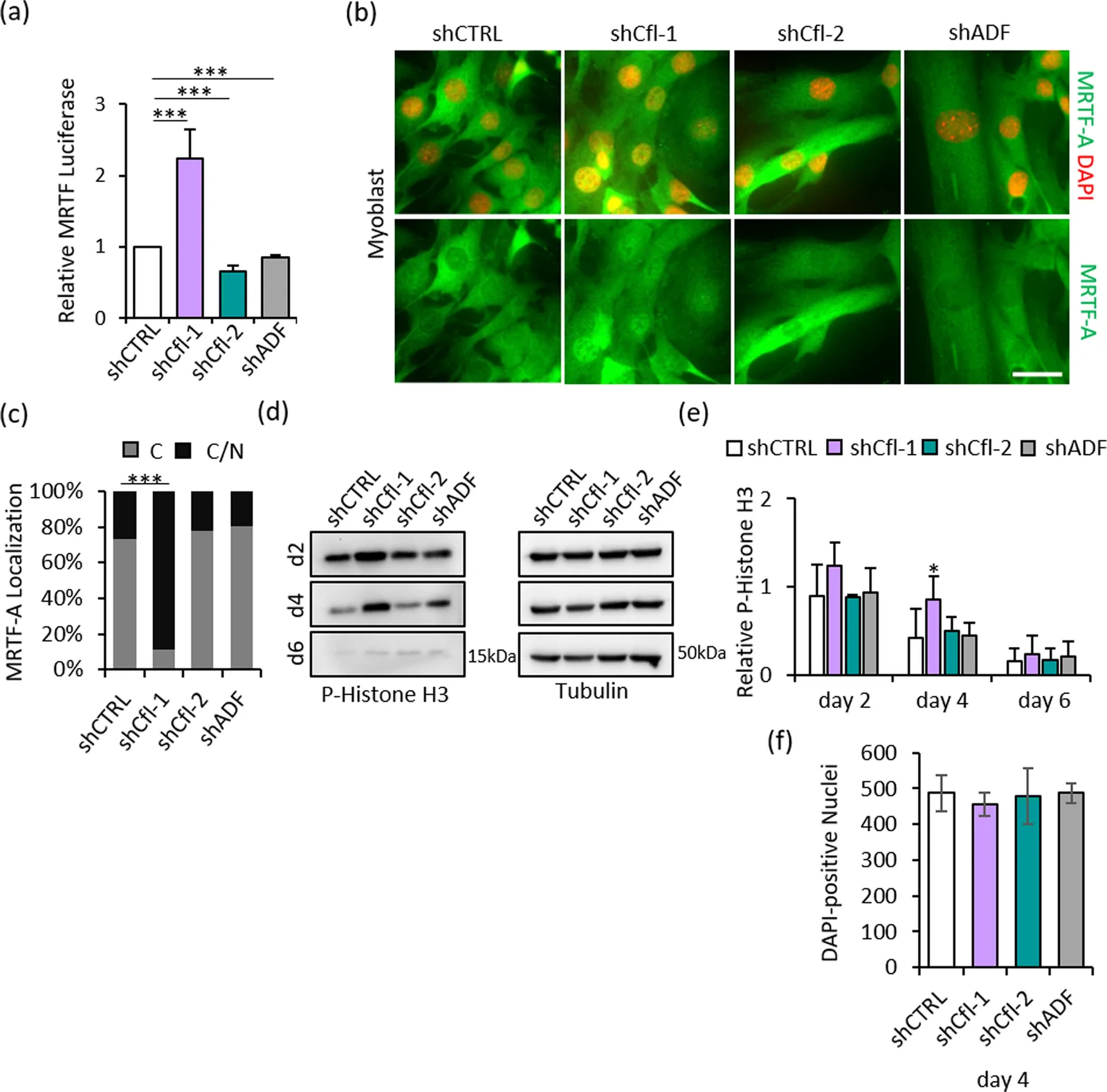
Cofilin-1 deficiency alters MRTF activity, intracellular localization, and mitotic behavior. (**a**) Relative MRTF luciferase reporter activity measured in the indicated Cofilin knockdown C2C12 myoblast cell lines. (**b**) Representative immunofluorescence images using antibodies against MRTF-A in Cofilin KD cell lines at the myoblast stage (day 0). (**c**) Quantification of the percentage of cells displaying cytoplasmic (C) or cytoplasmic/nuclear (C/N) MRTF-A localization (minimum 100 cells counted per group). (**d**) Immunoblots of the mitotic marker Phospho-Histone H3 (P-Histone H3) in Cofilin knockdown cells during differentiation days 2, 4, and 6. (**e**) Quantification of Phospho-H3 immunoblots. (**f**) Quantification of total cell density based on DAPI nuclear counts at differentiation day 4 (minimum 10 random images). Error bars, SD (*n* = 4) for (a, f), and SEM (*n* = 4) for (e). Statistical significance was determined using Student’s t-test for (a, e, f) and Chi-square test with post-hoc pairwise Holm-Bonferroni correction for (c). (**p < 0.01, ***p < 0.001). Scale bar: 25 μm

Because Cofilin-1 knockdown resulted in an accumulation of F-actin bundles alongside severely impaired myoblast differentiation, we investigated the underlying mechanisms driving this phenotype. Cofilin-controlled actin dynamics communicate directly with transcription via serum response factor (SRF) and its mechanosensitive co-activators, Myocardin-Related Transcription Factors A and B (MRTF-A/B) [26]. We employed an MRTF/SRF-responsive luciferase reporter assay to measure MRTF activity. Knockdown of Cofilin-1 led to an approximately twofold increase in MRTF reporter activity compared to control cells (Fig. 4a). In contrast, shCofilin-2 and shADF cells displayed a decrease in reporter activity (Fig. 4a). To evaluate whether the elevated transcription in shCofilin-1 cells was driven by altered localization, we performed immunofluorescence staining for MRTF-A. In control myoblasts, MRTF-A was predominantly located within the cytoplasm. Conversely, Cofilin-1-deficient myoblasts exhibited an observable accumulation of MRTF-A within the nucleus (Fig. 4b), while no distinct shifts were detected in Cofilin-2 or ADF knockdown lines. To quantitatively validate this observation, we scored the proportion of cells with predominant nuclear MRTF-A localization. Quantification revealed a significant accumulation of nuclear MRTF-A specifically in shCofilin-1 cells compared to control, whereas shCofilin-2 and shADF cells exhibited a slight, non-significant reduction in nuclear MRTF-A, closely reflecting the luciferase reporter activity trends (Fig. 4c).

During skeletal muscle development, myoblasts withdraw from the cell cycle to initiate terminal differentiation and fusion. Because Cofilins play a role in executing cell division, we investigated whether mitotic exit was perturbed in our knockdown lines by tracking Phospho-Histone H3 (P-H3), a marker of condensed mitotic chromatin. In control cells, P-H3 levels progressively declined from day 2 to day 6, reflecting normal, timely cell cycle withdrawal. In contrast, shCofilin-1 cells maintained higher P-H3 levels compared to controls, culminating in a significant elevation at day 4 (Figs. 4d, e). Neither Cofilin-2 nor ADF knockdowns exhibited deviations from the control timeline. Since P-H3 is only present in dividing cells, this persistence indicates that Cofilin-1-deficient cells fail to complete mitotic exit. To determine whether this persistent P-H3 signal reflected active hyperproliferation or mitotic arrest, we quantified overall cell density by scoring DAPI-stained nuclei at day 4. Total nuclear counts revealed no significant increase in cell density in shCofilin-1 cultures compared to control, with shCofilin-1 cells showing a subtle downward trend (Fig. 4f). This confirms that elevated P-H3 reflects an accumulation of cells stalled during late M-phase rather than active hyperproliferation.

### Gradual reduction of Cofilin-1 levels reveals a dose-dependent effect on myoblast fusion

Based on our observation that Cofilin-1 levels naturally decline during the normal progression of C2C12 differentiation, we hypothesized that this downregulation might be a required regulatory event rather than a mere byproduct of the myogenic program. To test whether a gradual reduction would differentially affect myotube formation, we titrated the volume of shRNA-encoding viral supernatant in transient transductions of C2C12 cells without antibiotic selection, immediately followed by the induction of differentiation. We reasoned that altering viral input would adjust population-wide transduction efficiency, allowing us to establish cultures with varying overall degrees of Cofilin-1 depletion. This strategy enabled the assessment of dose-dependent effects of global Cofilin-1 reduction during early differentiation.

**Fig. 5:**
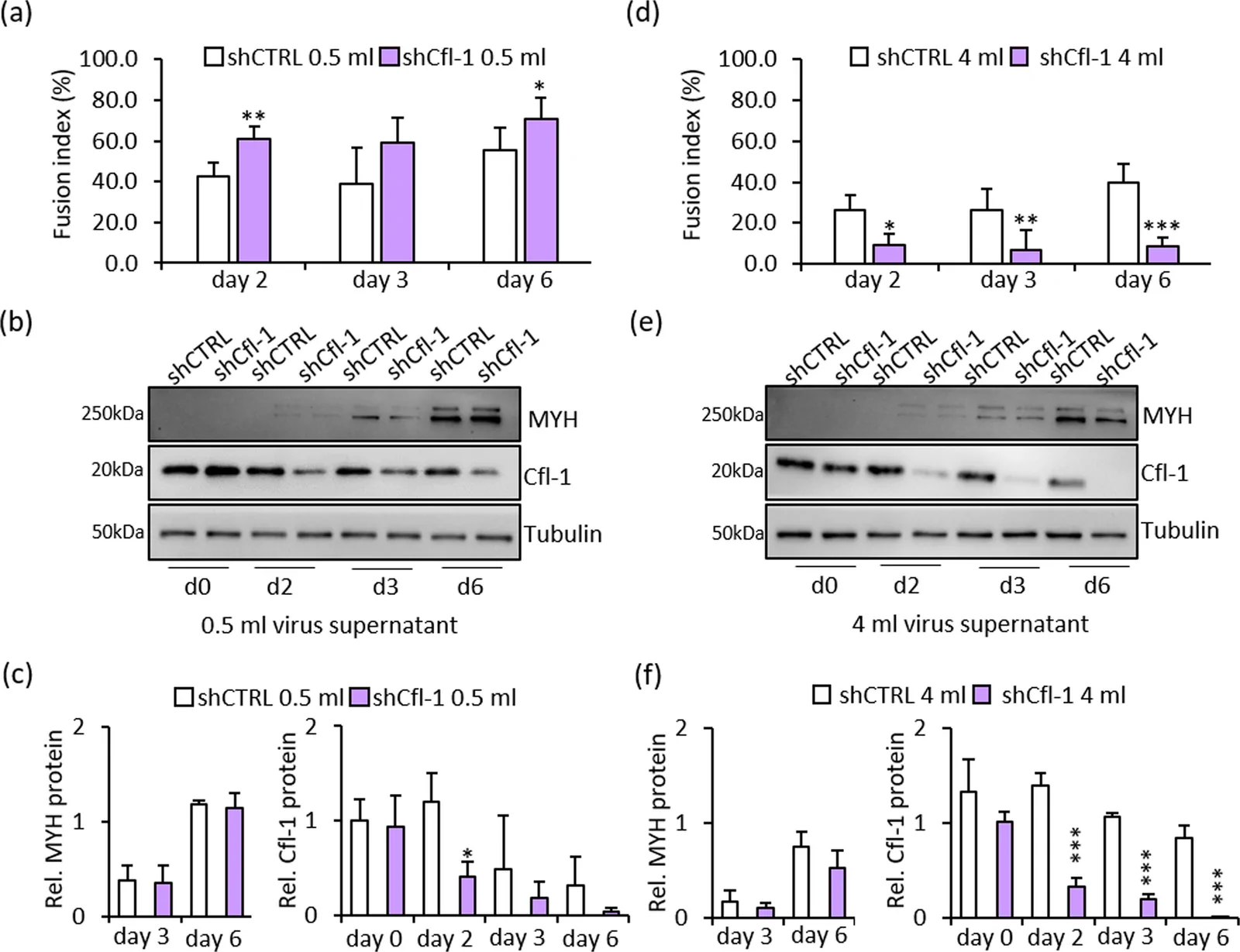
Gradual reduction of Cofilin-1 levels differentially modulates myoblast fusion efficiency. Transiently transduced C2C12 cells with low-level Cofilin-1 knockdown (0.5 mL virus-containing supernatant) (**a-c**) versus high-level Cofilin-1 knockdown (4 mL virus-containing supernatant) (**d-f**). (**a**, **d**) Fusion indices calculated from MYH-positive myotubes. (**b**, **e**) Immunoblots and (**c**, **f**) corresponding quantification of MYH and Cfl-1 protein levels after normalization to Tubulin. Error bars represent SD (*n* = 10) for fusion indices (a, d) and SD (*n* = 3) for Western blot quantification (c, f). *p < 0.05, **p < 0.01, ***p < 0.001, Student’s t-test

A low volume of virus-containing supernatant (e.g., 0.5 mL) resulted in enhanced MYH-positive myotube formation compared to shCTRL cells (Fig. 5a). Increased fusion indices were detectable as early as day 2 and persisted through day 6 of differentiation. Western blot analysis for MYH and Cofilin-1 revealed a slight but significant decrease in Cofilin-1 expression at day 2 of differentiation, while MYH levels remained comparable to controls (Fig. 5b, c).

In contrast, a high volume of viral supernatant (e.g., 4.0 mL) reproduced the fusion-defective phenotype previously observed in our stable knockdown and knockout models (Fig. 5d). Consistently, Cofilin-1 levels decreased more robustly under these conditions, extending into later time points (Fig. 5e, f). Furthermore, MYH accumulation showed a slight decrease at days 3 and 6, in contrast to the unaltered MYH levels observed at low virus concentrations.

Taken together, these data suggest that myoblast fusion is highly sensitive to the precise extent of Cofilin-1 reduction; while high viral doses drive a robust, population-wide depletion that impairs differentiation, a moderate reduction in global Cofilin-1 availability enhances fusion efficiency.

### Stability of Cofilin mRNA and protein does not account for isoform switching during myogenic differentiation

**Fig. 6:**
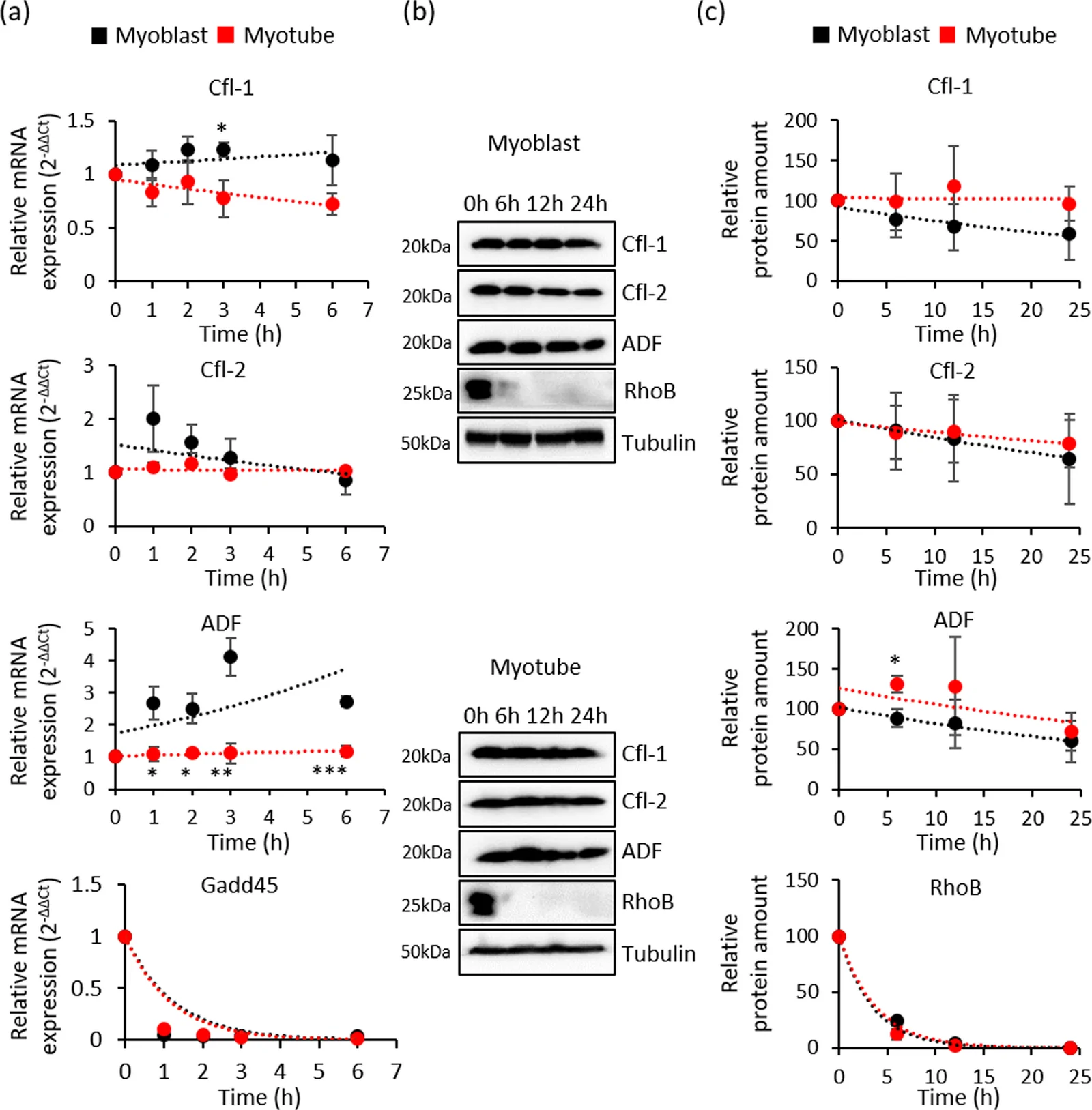
Differential expression of Cofilin isoforms during myogenesis is not explainable by altered mRNA or protein stability. (**a**) Relative mRNA abundance of Cofilin-1 (Cfl-1), Cofilin-2 (Cfl-2), and ADF following transcriptional arrest with Actinomycin D (ActD, 5 µg/mL) to assess mRNA decay kinetics in C2C12 myoblasts and differentiated myotubes at day 6. Samples were collected 1, 2, 3 and 6 h after ActD treatment, and mRNA levels were quantified by RT-qPCR. Gadd45 served as positive control for a short-lived mRNA. Values are expressed as double-normalized relative expression levels (normalized to housekeeping genes and to untreated control, set to 1). (**b**) Representative Western blot analysis of Cofilin-1, Cofilin-2, and ADF following inhibition of protein translation with cycloheximide (CHX, 75 µg/mL) to assess protein stability in myoblasts and myotubes at day 6. RhoB served as positive control for a short-lived protein. (**c**) Quantification of protein abundance shown in (b) at the indicated time points. Error bars represent SD (*n* = 4) for (a) and SD (*n* = 6) for (c). Statistical significance was assessed using Student’s t-test (*p < 0.05, **p < 0.01, ***p < 0.001)

We next investigated whether the reciprocal regulation of Cofilin isoforms observed during myogenic differentiation could be explained by differences in mRNA or protein stability rather than transcriptional control. To assess mRNA stability, transcription was inhibited in proliferating C2C12 myoblasts and differentiated myotubes (day 6) using Actinomycin D, and the decay kinetics of the Cofilin transcripts were analyzed over a 6-hour time course (Fig. 6a). Despite high experimental variation, the mRNAs of all Cofilin isoforms appeared highly stable in both myoblasts and myotubes, exhibiting no detectable negative rate constants within the measured window. Roughly estimated half-lives were all above 10 h, exceeding the experimental time course and suggesting high overall stability in both differentiation states. In contrast, Gadd45 transcripts, which are known for rapid turnover, displayed the expected decay kinetics with a calculated half-life of approximately 50 min, thereby confirming effective transcriptional inhibition.

To evaluate protein stability, translation was inhibited using Cycloheximide, and protein levels were analyzed at 6, 12, and 24 h post-treatment (Fig. 6b). As a validation of experimental efficacy, the short-lived protein RhoB exhibited the expected rapid depletion. Quantification of the Cofilin isoforms revealed that relative protein degradation rates did not differ significantly at any time point (Fig. 6c). While exponential trend lines suggested a slightly faster Cofilin-1 turnover in myoblasts, the protein remained highly stable in both myoblasts and myotubes. Collectively, these results indicate that neither differential mRNA stability nor altered protein degradation rates account for the reciprocal regulation of Cofilin isoforms during myogenic differentiation, suggesting instead that this regulation occurs predominantly at the level of gene transcription.

### Dynamic Ser3 phosphorylation modulates Cofilin-1 function during early myogenic differentiation

**Fig. 7:**
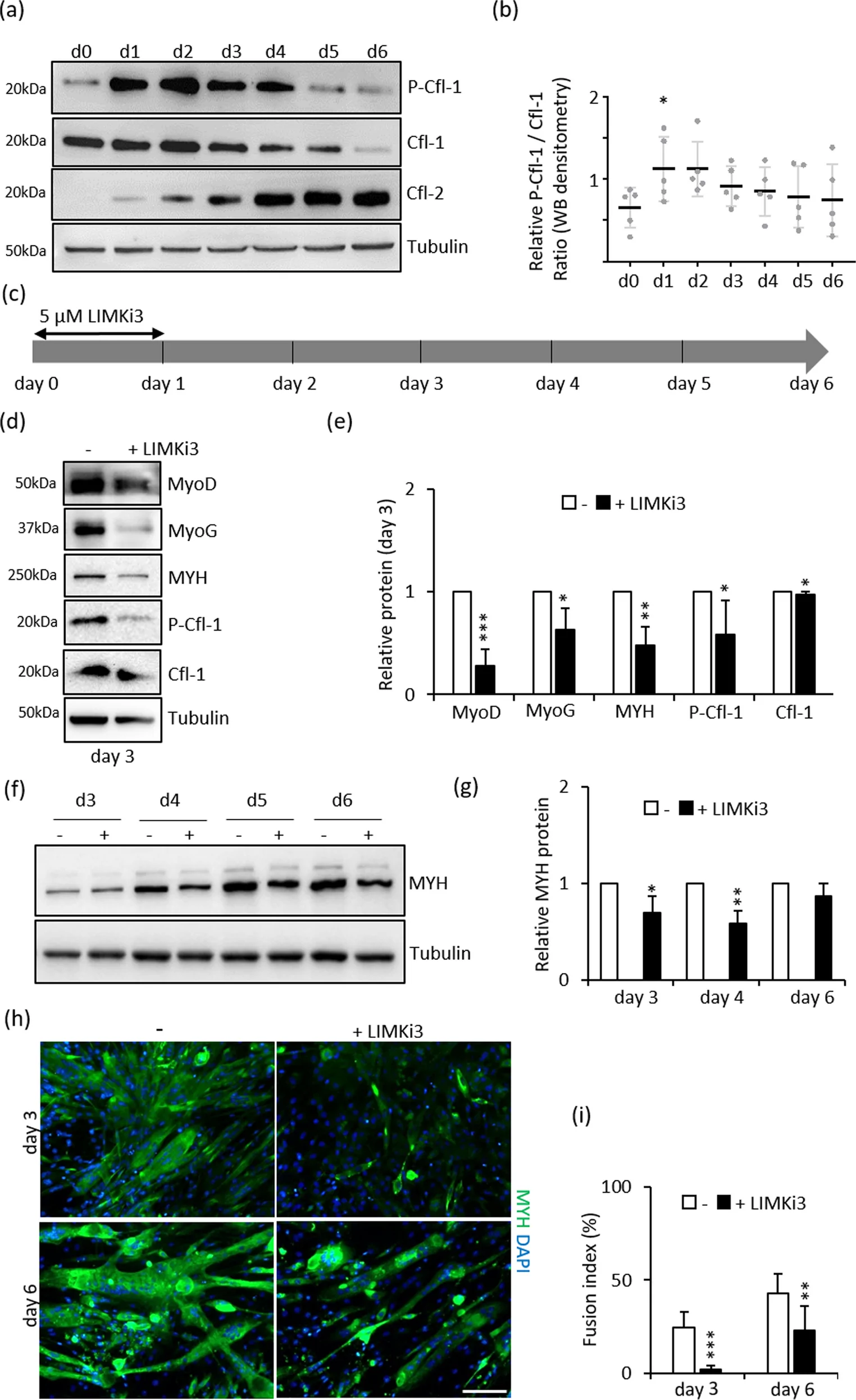
Dynamic Ser3 phosphorylation regulates Cofilin-1 activity during early myogenic differentiation. (**a**) Representative Western blot analysis of total Cofilin-1, Ser3-phosphorylated Cofilin-1 (P-Cfl-1) and Cofilin-2 during C2C12 myoblast differentiation. (**b**) Quantification showing the ratio of P- Cofilin-1 to total Cofilin-1 across differentiation days. Ratios larger than 1 result from arbitrary densitometric units. (**c**) Schematic representation of LIMK inhibition time course (LIMKi3) during differentiation. (**d**) Western blots of differentiation markers MyoD, Myogenin (MyoG), Myosin Heavy Chain (MYH), Cofilin-1 and P–Cofilin-1 following overnight treatment with LIM kinase inhibitor 3 (LIMKi3, 5 µM) with samples collected on day 3. (**e**) Quantification of Western blot data shown in (**d**). (**f**, **g**) Western blot analysis and quantification of MYH through differentiation day 3–6 post LIMKi3-treatment. (**h**) Representative immunofluorescence images showing MYH-positive myotubes in control (CTRL) and LIMKi3-treated cultures. (**i**) Quantification of the fusion index calculated from MYH-positive myotubes. A total of 10 fields from two independent experiments were analyzed for each condition. Error bars represent SD (*n* = 5) for (b), SD (*n* = 3) for (e, g), and SD (*n* = 10) for (i). Statistical significance was determined using Student’s t-test (*p < 0.05, **p < 0.01, ***p < 0.001). Scale bar: 100 μm

Cofilin-1 activity is negatively regulated by phosphorylation at Ser3, which inhibits its interaction with F-actin. To determine the dynamics of Ser3 phosphorylation of Cofilin-1 during differentiation, we quantified Ser3-phosphorylated Cofilin-1 using phospho-specific antibody. Unexpectedly, P-Ser3-Cofilin-1 levels increased sharply during the first two days of differentiation, whereas total Cofilin-1 protein levels gradually declined over the course of differentiation (Fig. 7a). Consequently, the ratio of phosphorylated to total Cofilin-1 was significantly elevated on differentiation days 1 and 2, before partially declining at later stages (Fig. 7b). These data indicate a temporally restricted inactivation of Cofilin-1 activity during early myogenesis.

Cofilin-1 is a well-established downstream target of the LIM kinases: LIMK1 and LIMK2. Publicly available expression datasets indicate that both LIMK1 and LIMK2 are expressed in C2C12 myoblasts and decrease during differentiation (Fig. S2; GSE4694). To assess whether LIMK-mediated phosphorylation of Cofilin-1 functionally contributes to myogenic differentiation, we pharmacologically inhibited LIM kinase activity using LIMK inhibitor 3 (LIMKi3/BMS-5) overnight between days 0 and 1, followed by continued culture in standard differentiation medium until day 6 (Fig. 7c). This treatment effectively reduced Ser3 phosphorylation of Cofilin-1 and resulted in significantly decreased expression of the differentiation markers MyoD, Myogenin, and MYH when analyzed at day 3 (Figs. 7d, e). The persistence of these defects through day 3, despite the inhibitor being present only during the initial 24-hour window, suggests that the early peak of Cofilin-1 phosphorylation observed on days 1 and 2 is required for the initiation of the myogenic program.

To further validate the long-term impact of this early inhibition, we monitored the accumulation of Myosin Heavy Chain (MYH) from day 3 to day 6. Western blot analysis revealed a significant reduction in MYH expression at day 3 and day 4 in transiently treated cells, followed by a partial biochemical recovery by day 6 once the transient inhibitor effect subsided (Fig. 7g). This temporal profile was corroborated by immunofluorescence analysis and fusion index calculations, which demonstrated a severe blockade of myoblast fusion at day 3 followed by a partial recovery in fusion capacity by day 6 (Fig. 7h, i).

To confirm that the initial fusion impairment was not merely a secondary consequence of non-specific cytotoxicity or population collapse, we evaluated metabolic viability via an XTT assay alongside DAPI-stained nuclear counts (Fig. S3). LIMKi3 treatment resulted in a modest, statistically significant reduction in cell density (~ 20–30%) and metabolic activity (~ 75–80% remaining viable relative to control) across time points (Fig. S3a, b). However, while cell viability remained at ~ 76.7% at day 3, the fusion index dropped precipitously from ~ 23% in control cells to < 2% in LIMKi3-treated cultures representing a > 90% reduction in fusion efficiency (Fig. 7i). Furthermore, while cell density and viability remained virtually stable between day 3 and day 6 (~ 75% of control), LIMKi3-treated cells exhibited a clear recovery in both MYH expression and fusion capacity by day 6 (recovering from a > 90% fusion blockade at day 3 to reach over 50% of control levels by day 6) (Fig. 7f–i, S3). This clear disparity between a modest ~ 20% drop in cell density and a temporary, near-total blockade of fusion-followed by parallel biochemical and structural recovery-confirms that transient LIMK inhibition induces a direct, reversible kinetic delay in myogenic differentiation rather than non-specific population depletion.

Notably, our analysis focused on Cofilin-1 dynamics, as it remains the prominent isoform during the early stages of differentiation. While the Phospho-Ser3 antibody recognizes a motif conserved across all three ADF/Cofilin isoforms, the corresponding Western blot profiles primarily reflect Cofilin-1 phosphorylation during early myogenesis, given its high relative abundance in myoblasts compared to Cofilin-2 or ADF (Fig. S4). To definitively test whether rising Cofilin-2 levels during late differentiation contribute to the Phospho-Ser3 signal and potentially skew the P-Cfl/total Cfl ratio, we tracked Phospho-Ser3 kinetics across differentiation (Days 0, 4, and 6) in a Cofilin-2-knockout C2C12 line (Fig. S4). The temporal decline of the Phospho-Ser3 signal was identical in both control and Cofilin-2-KO cells, confirming that Cofilin-2 expression does not significantly contribute to or confound the observed Phospho-Ser3 Western blot profiles. These findings indicate that the dynamic Ser3 phosphorylation of Cofilin-1 is essential for the proper progression of myogenic differentiation. Disrupting this regulatory checkpoint by transiently inhibiting early LIMK-dependent phosphorylation severely impairs the downstream myogenic differentiation program.

## Discussion

Skeletal muscle differentiation demands a dramatic shift in actin dynamics, transitioning from a highly fluid network that supports motile, dividing precursors into an organized, force-generating contractile apparatus. Our study identifies the ADF/Cofilin family as a crucial coordinator of this structural transformation during C2C12 myogenesis. We demonstrate that successful muscle development depends on a temporally synchronized, multi-layered regulatory network. Rather than serving redundant roles, the systematic clearance of Cofilin-1 and ADF-coupled with the parallel induction of the muscle-specific Cofilin-2-defines a strict functional transition. Specifically, our findings uncover a critical, dosage-sensitive threshold for Cofilin-1 that orchestrates both transcriptional commitment and the physical cell-cycle exit mandatory for terminal myoblast fusion.

While Cofilin-1 and ADF are highly homologous, they exhibit distinct kinetic and physiological specializations. Although broadly expressed across non-muscle tissues and early embryonic muscle, they are systematically replaced by Cofilin-2 during myogenesis, which becomes the predominant postnatal isoform [19, 20, 29, 30]. In a cell environment, Cofilin-1 preferentially localizes to regions of rapid actin turnover, whereas Cofilin-2 targets the highly organized, less dynamic actin networks of developing myofibers and localizes to the I-bands of nascent myofibrils [13, 20, 31]. Because Cofilin-2 exhibits a higher affinity for, and a slower dissociation rate from, ADP-actin filaments compared to Cofilin-1 and more effectively suppresses actin-tropomyosin interactions, it dampens actin turnover to stabilize F-actin during nascent myofibrillar assembly [13, 16, 20, 31, 32]. Consequently, while proliferating myoblasts rely on the aggressive actin turnover driven by Cofilin-1 to navigate migration and cytokinesis, terminal differentiation requires this fluid actin machinery to be systematically decommissioned.

### Cofilin-1 performs a dominant and non-redundant role in early myogenesis

Our results demonstrate that Cofilin-1 acts as the primary, non-redundant regulator during the initial stages of myogenesis. Expression analysis revealed a clear isoform transition upon differentiation: Cofilin-1 and ADF levels progressively decline, whereas the muscle-specific isoform Cofilin-2 is strongly upregulated following the induction of differentiation (Fig. 1). This reciprocal pattern is well-supported by transcriptomic analyses of differentiating C2C12 cells (Fig. S5; GSE4694, GSE989) [33, 34] and mouse skeletal muscle injury models (GSE45577) [35]. These observations suggest that myogenic differentiation involves a coordinated transition between cytoskeletal regulatory programs, moving from the highly dynamic actin-remodeling machinery required for myoblast proliferation and migration to components optimized for the stable structural assembly of maturing muscle cells [36].

Although these isoforms share conserved actin-binding domains, their unique biochemical specializations dictate their cellular roles. Cofilin-1 preferentially binds ADP-actin in older filaments to promote rapid turnover in dynamic environments [16]. In contrast, Cofilin-2 displays a broader affinity for multiple nucleotide states (ATP-, ADP-Pi-, and ADP-actin), enabling it to interact effectively with the highly organized actin networks characteristic of mature contractile architectures [16, 30, 31]. Our data support a model where proliferating myoblasts rely on the high-turnover kinetics of Cofilin-1, while Cofilin-2 becomes functionally relevant only as cells transition toward the structurally ordered environment of the maturing myotube.

Consistent with this framework, the loss of Cofilin-1 in our knockout and knockdown experiments resulted in pronounced morphological defects, including enlarged cell size and multinucleation. These phenotypes are characteristic of cytokinesis failure and match previous reports describing mitotic defects and cytoskeletal over-stabilization following Cofilin-1 depletion [37, 38]. Such structural defects occur when actin filaments fail to undergo normal severing and instead accumulate into thick, stabilized bundles or stress-fiber-like structures (Fig. 2d) [37, 38].

In contrast, the deletion of Cofilin-2 produced comparatively mild phenotypes during early differentiation, and even slightly potentiated the early fusion program. This observation aligns with the developmental timing of Cofilin-2 expression, which peaks during late-stage myotube maturation and myofibrillar organization rather than during the initial commitment and fusion phase [39, 40].

### Cofilin-1 orchestrates the proliferation-differentiation switch by gating MRTF activity and mitotic exit

Our results support a direct mechanistic link between Cofilin-1 abundance and the control of the myoblast proliferative state. The transcriptional co-activator MRTF-A serves as a sensitive molecular rheostat that couples actin dynamics to gene expression via the Serum Response Factor (SRF) [26, 27]. In Cofilin-1-deficient cells, the loss of actin-severing activity shifts the cytoskeletal equilibrium toward stable F-actin filaments, depleting the monomeric G-actin pool that normally restrains the coactivator. This structural shift explains the nuclear accumulation of MRTF-A and the increase in MRTF/SRF reporter activity observed in our shCofilin-1 models (Fig. 4). Intriguingly, shCofilin-2 and shADF cells displayed a decrease in reporter activity. This divergence indicates that these isoforms are not functionally redundant in myoblasts; while Cofilin-1 acts as a negative regulator of MRTF-A, Cofilin-2 and ADF appear necessary for sustaining baseline SRF homeostasis.

During normal myogenesis, terminal commitment and cell cycle withdrawal require a coordinated downregulation of MRTF-A, which is driven post-transcriptionally by a specialized muscle miRNA network [25]. Chronically high nuclear MRTF-A signaling actively blocks this developmental transition, likely by driving progenitor-retaining factors such as Pax7 and forcing muscle precursors to remain in a non-differentiating state [24]. This transcriptional circuitry is highly consistent with the delayed induction of the early differentiation markers MyoD and Myogenin observed in our Cofilin-1-depleted cultures (Figs. 2 and 3).

Differentiating myoblasts progressively clear the mitotic marker Phospho-Histone H3 (P-H3) as they successfully withdraw from the cell cycle into a post-mitotic state. However, shCofilin-1 cells maintained elevated P-H3 levels, culminating in a significant elevation at day 4 (Fig. 4). Because P-H3 marks condensed chromatin from early prophase through telophase, these sustained levels do not necessarily represent productive hyperproliferation. Crucially, because these assays are conducted following the switch to serum-depleted Differentiation Medium (DM), the environment provides a strong extrinsic cue for *G*_0_ cell-cycle withdrawal that naturally dampens global proliferation across all conditions. Within this non-permissive proliferative environment, our quantification of total DAPI-stained nuclei at day 4 confirmed that Cofilin-1 deficiency does not increase overall cell density, ruling out active hyperproliferation or expansion of the progenitor pool (Fig. 4f). Instead, these observations reflect a structural delay or arrest in late M-phase/cytokinesis. Cofilin-1-mediated actin filament severing is essential for contractile ring disassembly and successful mitotic exit [22, 37, 38]; in its absence, an unsevered, rigid cortical actin network creates a physical impediment to cell division. Therefore, the dysregulation of actin dynamics in Cofilin-1-deficient cells establishes a dual barrier to differentiation: it sustains nuclear MRTF-A activity to transcriptionally suppress early myogenic commitment, while simultaneously hindering the physical completion of M-phase and cell cycle exit mandatory for terminal differentiation.

### Cofilin-1 operates within a dosage-sensitive regulatory window

Our study indicates that Cofilin-1 does not act as a simple binary switch but rather operates within a narrow, permissive regulatory window. Viral titration experiments revealed that while a moderate reduction of Cofilin-1 levels actually enhanced the fusion index, more severe depletion significantly impaired myotube formation (Figs. 2, 3 and 5). A moderate decrease in Cofilin-1 may facilitate the partial stabilization of the cortical actin cytoskeleton required for the assembly of fusion-competent structures and the organization of the “fusogenic synapse” between myoblasts [5–7]. In contrast, excessive depletion likely compromises the rapid filament turnover necessary for the massive cytoskeletal rearrangements that precede membrane merging.

Similar dosage sensitivity has been reported in bovine myoblasts, where both overexpression and excessive depletion of Cofilin-1 disrupt myogenic progression [41]. The importance of maintaining precise Cofilin-1 levels is further underscored by clinical observations in muscle pathology. For instance, Vignier et al. (2021) demonstrated that the abnormal accumulation of Cofilin-1 in cardiomyopathy caused by lamin A/C gene mutation destabilizes sarcomere actin and leads to muscle weakness [42]. Together, these findings suggest that the tight regulation of Cofilin-1 dosage is a universal requirement, critical for both developmental myogenesis and the maintenance of adult muscle integrity.

#### Dynamic regulation through the LIMK-Cofilin phosphorylation cycle

Beyond the regulation of protein abundance, Cofilin-1 activity is rapidly modulated via post-translational modification to navigate the immediate demands of early differentiation. We observed a transient increase in Cofilin-1 phosphorylation at Ser3 during the first 48 h of differentiation (Fig. 7a), a modification known to temporarily inactivate its actin-severing function [17, 18]. This transient inactivation phase creates a critical mechanical window, permitting the rapid accumulation of stable F-actin structures necessary for early morphological shifts, such as the cell elongation and alignment that must precede membrane fusion. Importantly, control experiments in Cofilin2-knockout myoblasts confirmed that this temporal Phospho-Ser3 profile reflects genuine Cofilin-1 dynamics without being confounded by rising Cofilin-2 levels during late differentiation (Fig. S4c).

The physiological necessity of this mechanism is clearly demonstrated by our small-molecule interventions. Inhibition of LIM kinase activity using LIMKi3 not only disrupted the initial induction of the early differentiation markers MyoD and Myogenin, but also severely impaired downstream myotube formation and MYH accumulation (Fig. 7). While LIMK inhibition induced a modest, expected reduction in cell density and metabolic viability (~ 20–25%), this minor cytostatic effect cannot account for the near-total blockade of fusion observed at day 3 (> 90% reduction) (Fig. 7i, S3). Crucially, as the transient inhibitor effect subsided, both MYH expression and fusion capacity exhibited a clear recovery by day 6 under static cell density conditions (Fig. 7f-i, S3). This temporal recovery demonstrates that the loss of fusion reflects a direct, kinetic delay in myogenic membrane remodeling rather than non-specific cytotoxicity or population collapse.

The ratio of P-Cofilin-1 to total Cofilin-1 fluctuates dynamically across the differentiation timeline, indicating that a precise, timed phosphorylation-dephosphorylation cycle-rather than a static, constitutive activation or inhibition-is required to navigate early myogenesis. In contrast, Cofilin-2 is specialized to regulate filament length within the stable contractile apparatus and remains largely unperturbed by these early upstream LIMK dynamics [29, 36]. These findings collectively indicate that the dynamic Ser3 phosphorylation of Cofilin-1 serves as an essential regulatory checkpoint for the proper progression of myoblast fusion and muscle development.

## Conclusions

**Fig. 8:**
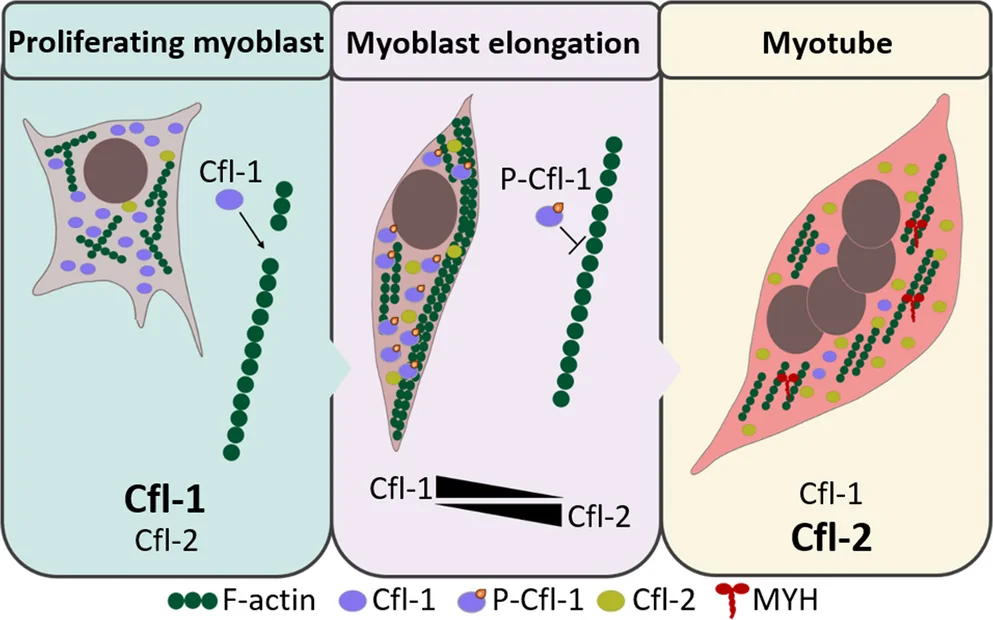
Proposed model of the Cofilin switch during myogenic differentiation. Proliferating myoblasts rely on high baseline levels of Cofilin-1 (Cfl-1) for rapid actin filament turnover. Upon the induction of differentiation and myoblast elongation, Cfl-1 activity is suppressed via LIMK-mediated Ser3 phosphorylation, allowing the stabilized accumulation of cortical F-actin networks. As myogenesis progresses, a transcriptional shift occurs from Cfl-1 to the muscle-specific Cfl-2, which is predominant in myotubes with their highly organized, less dynamic actomyosin system. P-Cfl-1, phospho-Cofilin-1, MYH, myosin heavy chain

In conclusion, our study establishes a model of myogenesis wherein the transition from proliferating myoblasts to differentiated myotubes is regulated by a synchronized, dual-layer control of the actin cytoskeleton. Phosphorylation via the LIMK pathway serves as a rapid, post-translational switch to temporarily pause Cofilin-1 activity, allowing early cellular remodelling to proceed. Concurrently, gradual transcriptional downregulation systematically removes the dominant Cofilin-1 pool from the cell, allowing the stable, muscle-specific architecture of Cofilin-2 to take its place (Fig. 8). Crucially, our results demonstrate that disrupting this actin-severing threshold triggers a multi-layered developmental barrier: the loss of Cofilin-1 simultaneously drives transcriptional changes characterized by the hyperactivation and nuclear accumulation of MRTF-A, while imposing a physical block that stalls normal cell-cycle exit.

The physiological necessity of this precise isoform transition is heavily underscored by human muscle pathologies. On one end of the spectrum, a failure to clear the non-muscle Cofilin-1 isoform leads to severe sarcomere disorganization and contractile force deficits [42]. On the other end, any loss of functional Cofilin-2 is equally catastrophic, as our data highlight a progressive reliance on this muscle-specific isoform during maturation. Indeed, Cofilin-2 mutations are a known cause of congenital nemaline myopathy, characterized by the structural collapse of the contractile apparatus and the aggregation of structural actin into “nemaline bodies” [43]. Ultimately, by showing how the precise control of Cofilin-1 abundance and its phosphorylation state coordinates both downstream signalling and structural dynamics, this study provides a holistic view of how cytoskeletal regulators integrate with broader molecular networks to dictate cell fate decisions during myoblast fusion and muscle development.

## Supplementary Information

Below is the link to the electronic supplementary material.

Supplementary Material 1 (PDF 1.18 MB)

Supplementary Material 2 (PDF 2.28 MB)

## Data Availability

All data generated or analysed during this study are included in this published article and its supplementary information files.

## Declarations

### Running title

Cofilin-1 dosage regulates myogenesis.

## Abbreviations

ActD: Actinomycin D
ADF: Actin Depolymerizing Factor
C2C12: Immortalized mouse myoblast cell line
Cav3: Caveolin-3
CHX: Cycloheximide
Cfl: 1-Cofilin-1
Cfl: 2-Cofilin-2
CRISPR: Clustered Regularly Interspaced Short Palindromic Repeats
DM: Differentiation medium
F: actin-Filamentous actin
G: actin-Globular actin
IF: Immunofluorescence
KD: Knockdown
KO: Knockout
LIMK: LIM kinase
LIMKi3: LIM Kinase Inhibitor 3 (BMS-5)
MRTF: A-Myocardin-related transcription factor A
MRF: Myogenic regulatory factor
MyoD: Myogenic differentiation 1
MyoG: Myogenin
MYH: Myosin Heavy Chain
MymK: Myomaker
MymX: Myomixer/Myomerger
PBS: Phosphate-buffered saline
RT: qPCR-Quantitative polymerase chain reaction
ROCK: Rho-associated coiled-coil containing protein kinase
ROI: Region of Interest
SD: Standard Deviation
shRNA: Short hairpin RNA
shCTRL: Scrambled shRNA control
SSH: Slingshot phosphatase
SRF: Serum response factor
WB: Western Blot
