# Supplementary material for: Dosage-sensitive regulation of Cofilin-1 and LIMK signaling controls myoblast differentiation and fusion
Source: Cell Mol Life Sci. 2026 Sep 18;83(1):338. doi: 10.1007/s00018-026-06437-1 (PMC13589735; doi:10.1007/s00018-026-06437-1)
Supplement: Supplementary file 1 [file 18_2026_6437_MOESM1_ESM.pdf]

Supplementary file

Dosage-sensitive regulation of Cofilin-1 and LIMK signaling controls myoblast differentiation and fusion

Dora Gjirlić, Anja Weber, Guido Posern[#], and Anurag Kumar Singh[#]

Institute for Physiological Chemistry, Medical Faculty, Martin Luther University Halle-Wittenberg, 06114 Halle (Saale), Germany

Figure S1

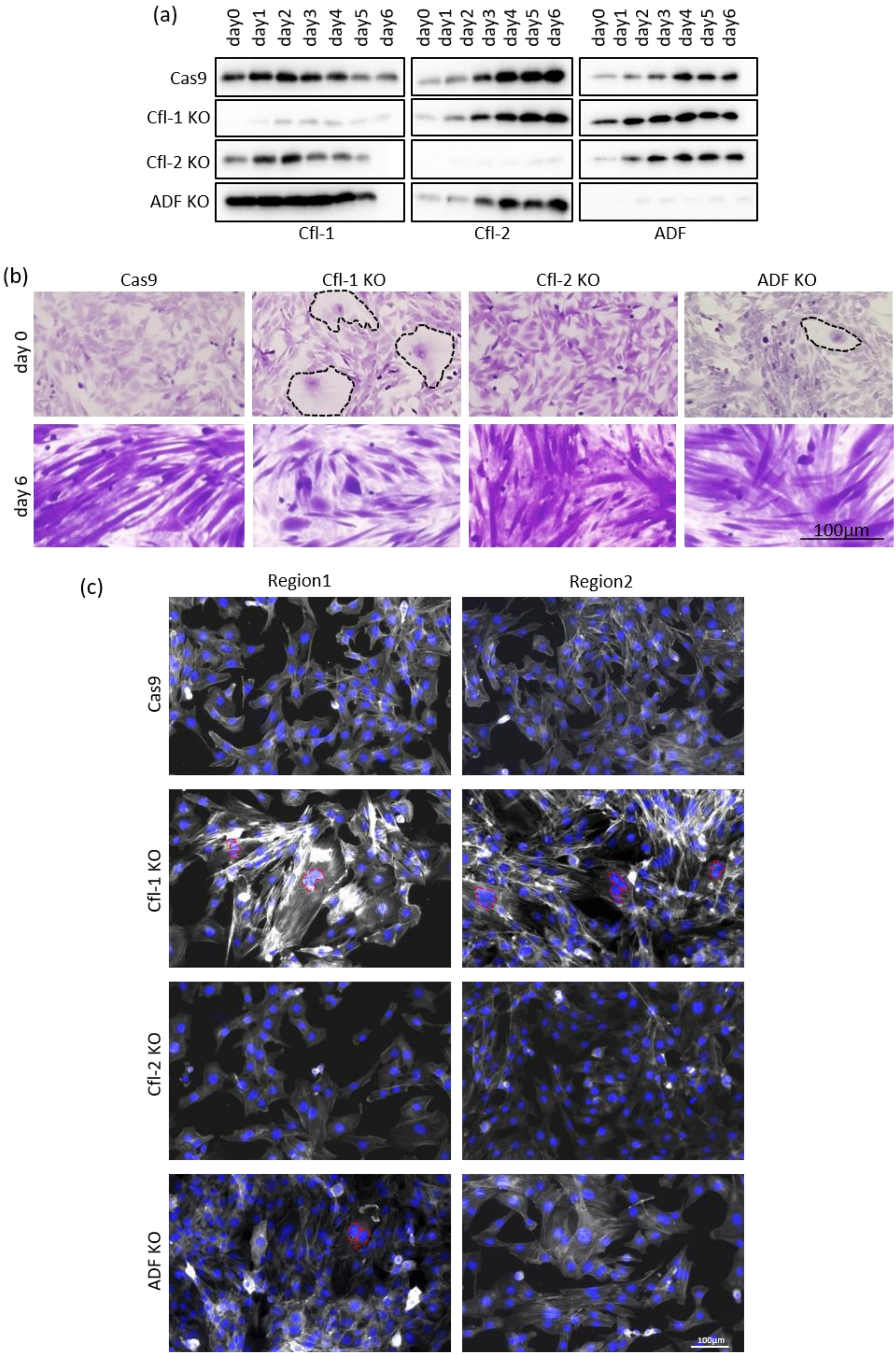

(a)
day0
day1
day2
day3
day4
day5
day6
Cas9
Cfl-1 KO
Cfl-2 KO
ADF KO
Cfl-1
Cfl-2
ADF
(b)
Cas9
Cfl-1 KO
Cfl-2 KO
ADF KO
day 0
day 6
100μm
(c)
Region1
Region2
Cas9
Cfl-1 KO
Cfl-2 KO
ADF KO
100μm

Figure S1. Validation of antibody specificity and morphological characterization of Cofilin family knockout C2C12 cell lines. (**a**) Representative Western blot analysis for Cofilin-1, Cofilin-2, and ADF/Destrin using lysates from Cas9 control cells and individual knockout (KO) C2C12 cell lines. Each antibody was tested across Cofilin-1 KO, Cofilin-2 KO, and ADF KO cells. (**b**) Crystal violet staining of Cas9 control and Cofilin family KO C2C12 cells at the myoblast stage (day 0) and after 6 days of differentiation, (**c**) Phalloidin staining of F-actin (white) and DAPI (blue) in Cas9 control and Cofilin KO myoblasts at differentiation day 0. Pink outlines were added to discern abnormal, polynucleated cells observed within the Cfl-1 KO and ADF KO pooled populations prior to the induction of differentiation (day 0).

Figure S2

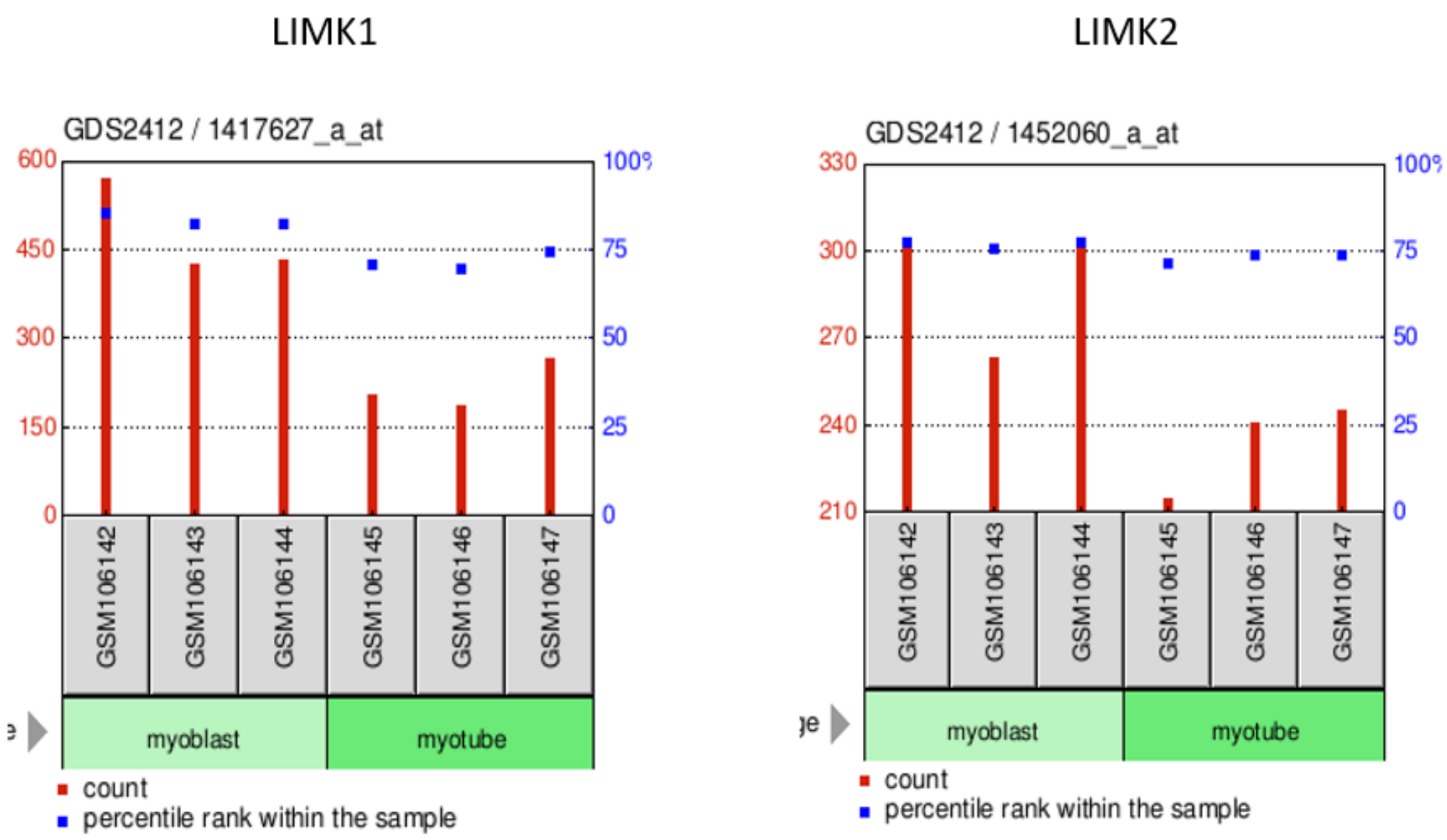

Figure S2. Re-analysis of GEO dataset reveals reduced LIM kinase expression during myogenic differentiation. Expression of LIM kinase family members (LIMK1 and LIMK2) during C2C12 differentiation was examined by re-analysis of the GEO dataset GSE4694, generated using the Affymetrix Mouse Genome 430 2.0 microarray platform [1]. Compared with proliferating C2C12 myoblasts, differentiated myotubes display reduced expression of LIM kinase genes, consistent with

reduced phosphorylation-dependent inhibition of Cofilin during differentiation. Expression profile of Limk1, showing decreased expression upon differentiation. GEO profile: https://www.ncbi.nlm.nih.gov/geoprofiles/?term=31829858. Expression profile of Limk2, similarly demonstrating reduced transcript levels in differentiated myotubes. GEO profile: https://www.ncbi.nlm.nih.gov/geoprofiles/?term=31864255. These data support the notion that regulation of Cofilin activity during myogenesis may involve coordinated modulation of upstream regulatory kinases, including LIMK family members.

Figure S3

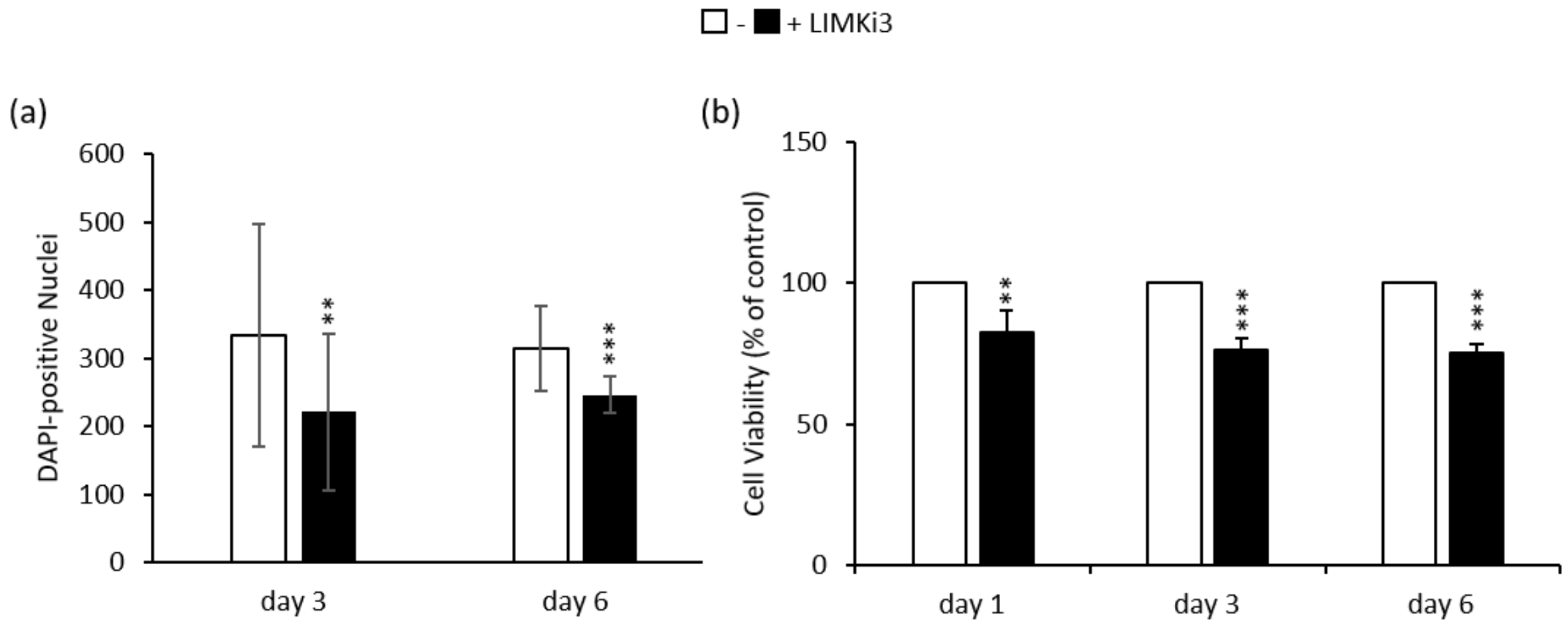

Figure S3. Transient LIMK inhibition moderately reduces cell density and metabolic viability. (a) Quantification of total cell density based on DAPI-positive nuclei counts at days 3 and 6 of differentiation in control (–) and LIMKi3-treated (+ LIMKi3) C2C12 cultures. **(b)** Cell viability and metabolic activity assessed by XTT assay at days 1, 3, and 6, expressed relative to vehicle-treated control cells. Error bars, SEM (n = 4). Statistical significance was determined using Student's t-test (**p < 0.01, ***p < 0.001).

Figure S4

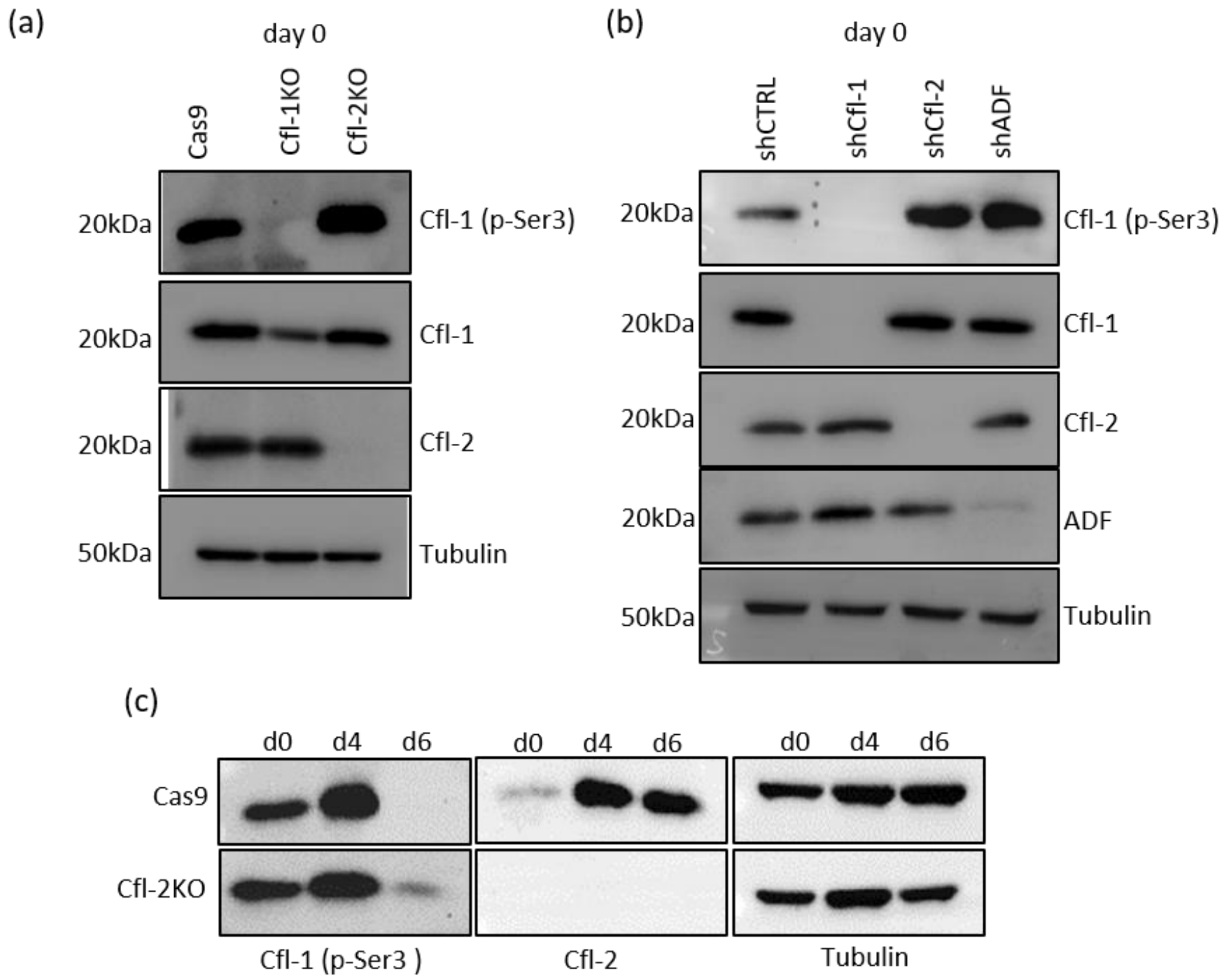

Figure S4. Validation of isoform specificity of Phospho-Cofilin (Ser3) antibody. (**a**, **b**) Representative Western blot profiles evaluating Phospho-Cofilin (Ser3) antibody reactivity across knockout (KO) and knockdown (KD) myoblast populations at day 0 of differentiation. (**c**) Representative Western blot analysis across the differentiation timeline (Days 0, 4, and 6) in control (Cas9) and Cfl2-KO cells. The complete absence of Cofilin-2 protein does not alter the temporal profile or intensity of Phospho-Ser3 levels, confirming that rising Cofilin-2 expression during late differentiation does not significantly contribute to or skew the observed Phospho-Cofilin signal. The distinct loss of signal in Cofilin-1-deficient conditions confirms that the Phospho-Ser3 signal predominantly reflects Cofilin-1 phosphorylation (P-Cfl-1) in this experimental setting.

Figure S5

Profile GDS586 / 99119_at
Title Myogenic differentiation timecourse (MG-U74A)
Organism Mus musculus

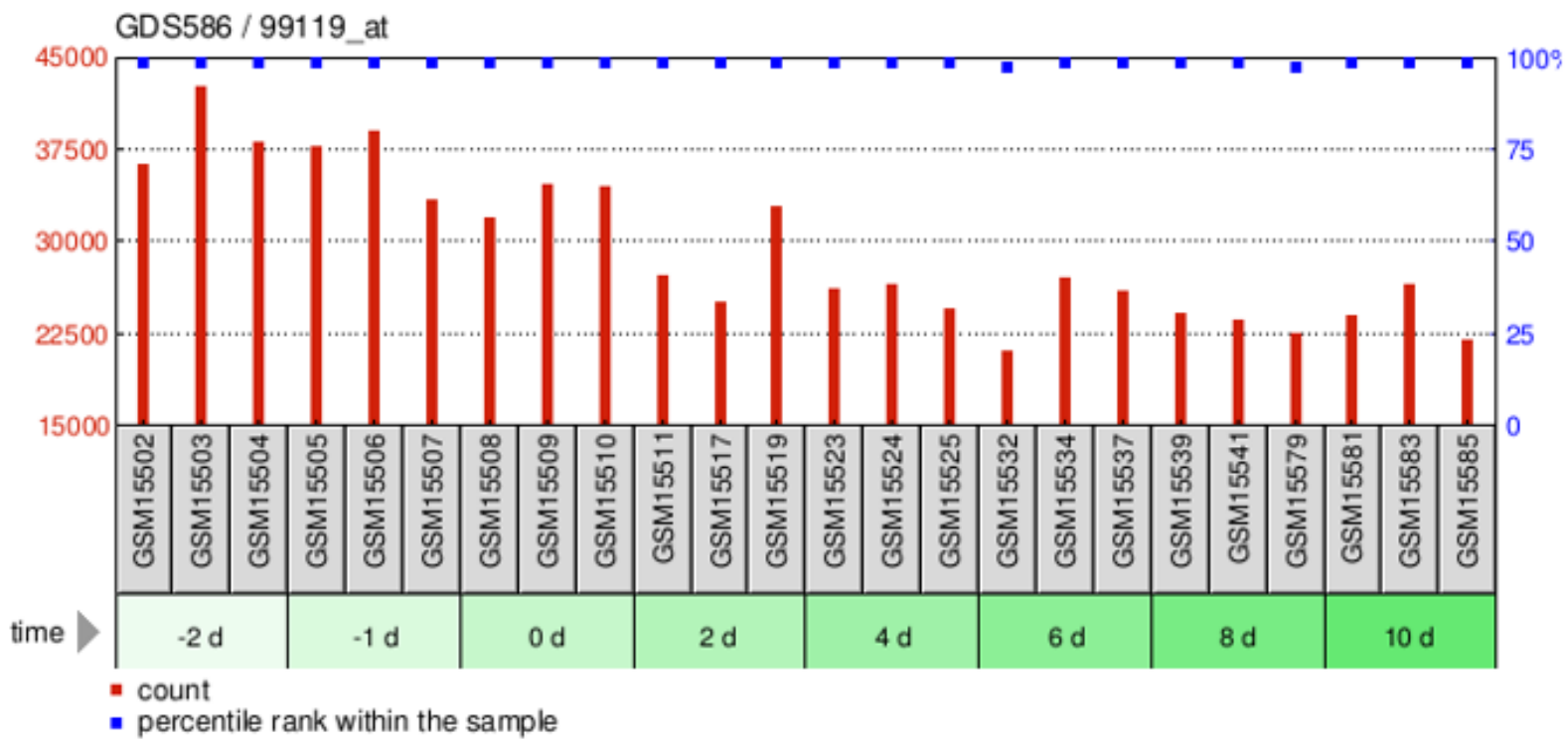

Cofilin-1

Profile GDS586 / 97549_at
Title Myogenic differentiation timecourse (MG-U74A)
Organism Mus musculus

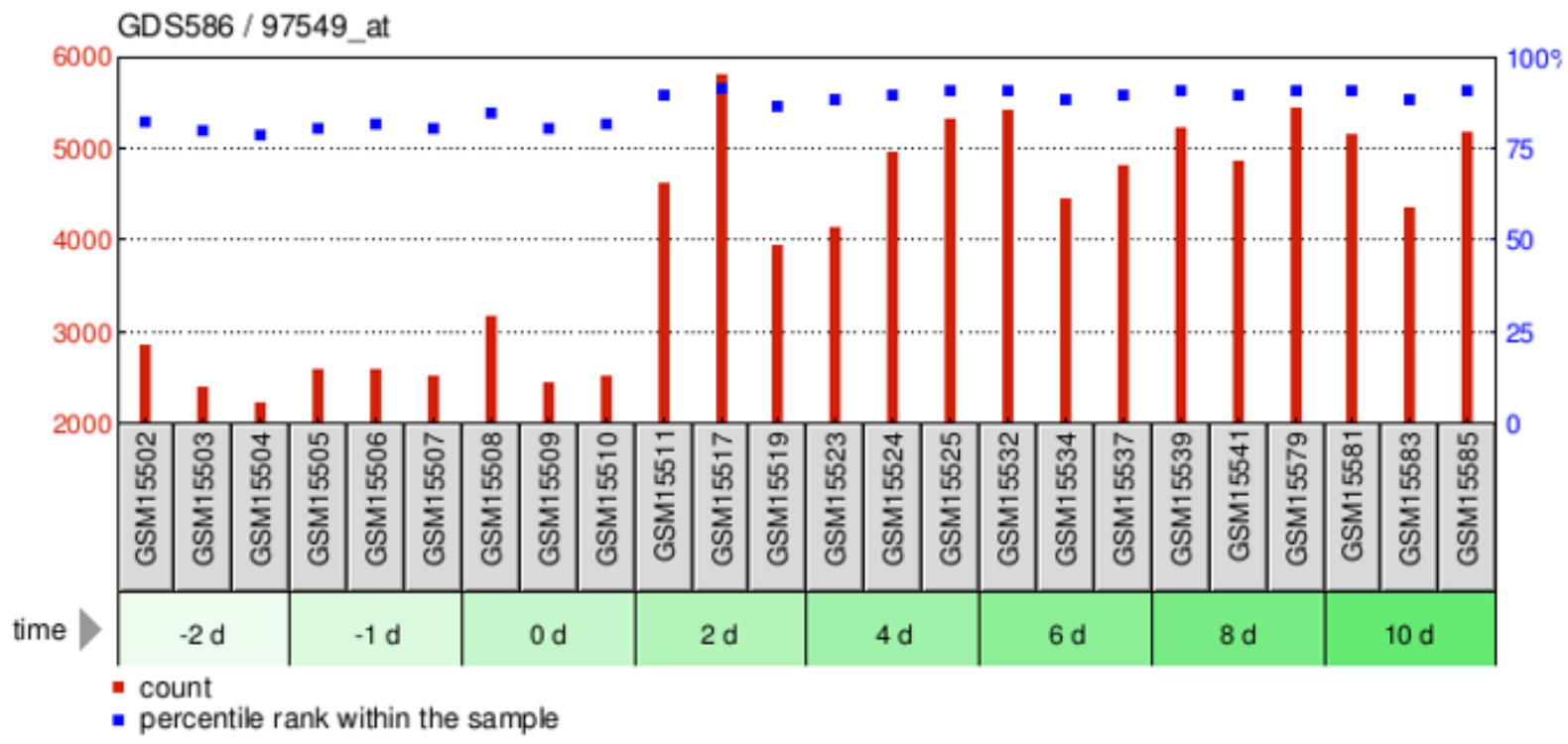

Cofilin-2

Profile GDS586 / 94492_at
Title Myogenic differentiation timecourse (MG-U74A)
Organism Mus musculus

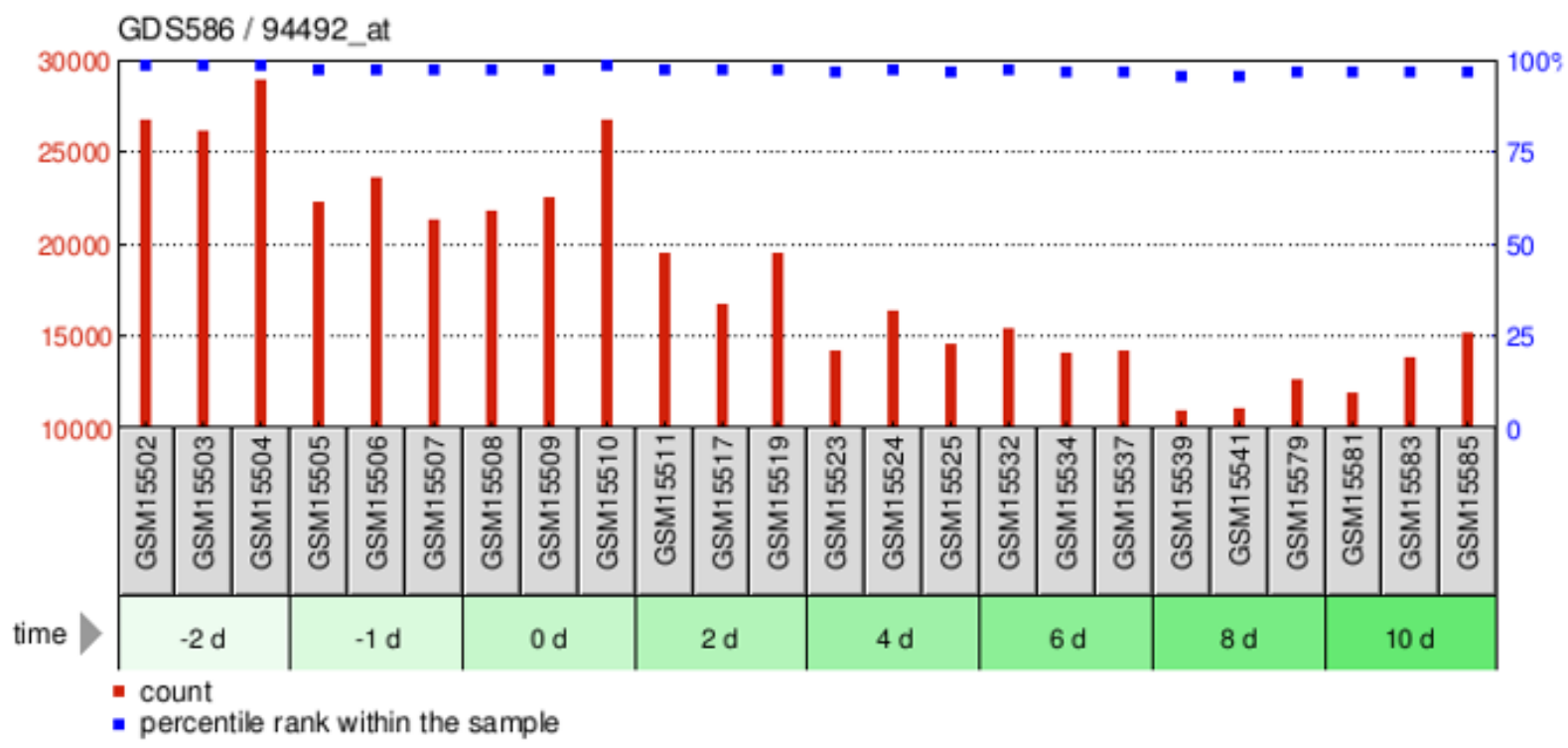

ADF/Destrin

Figure S5. Re-analysis of public transcriptomic dataset reveals dynamic regulation of ADF/Cofilin family members during myogenic differentiation. Expression dynamics of ADF/Cofilin family members were examined by re-analysis of the GEO dataset GSE989 [2], which profiles transcriptional changes during C2C12 myogenic differentiation. In this dataset, C2C12 myoblasts were induced to differentiate and sampled across multiple stages, including proliferating myoblasts (days −2 to −1), cell cycle withdrawal (day 0), and progressive myogenic differentiation and maturation of multinucleated myotubes (days 2–10). Re-analysis revealed distinct expression patterns among ADF/Cofilin family members: Cfl-1 (Cofilin-1) expression progressively declined during differentiation, consistent with reduced actin turnover associated with myotube maturation. GEO profile: https://www.ncbi.nlm.nih.gov/geoprofiles/4575939. ADF (Destrin, Dstn) expression similarly decreased during the differentiation process, indicating coordinated downregulation of non-muscle ADF/Cofilin isoforms. GEO profile: https://www.ncbi.nlm.nih.gov/geoprofiles/4572483. In contrast, Cfl-2 (Cofilin-2) expression displayed inverse regulation, showing progressive induction during differentiation, consistent with its known muscle-enriched expression pattern and role in mature muscle fibers. GEO profile: https://www.ncbi.nlm.nih.gov/geoprofiles/4574807. Together, these publicly available transcriptomic data support the isoform-specific expression switch observed in our experimental system.

Supplementary Table S1: Primer list used for the generation CRISPR/Cas9 KO and shRNA KD cells and quantitative real-time PCR (RT-qPCR).

**primer name**	**primer sequence**
Cfl-1-F	AGACAAGGACTGCCGCTATG
Cfl-1-R	TTGCTCTTGAGGGGTGCATT
Cfl-2-F	GCAGATCTTGGTGGGTGACA
Cfl-2-R	CACTTTCAGGAGCCCAGAATATAA
ADF-F	TCTCCTGCTTTTGCTACCCG
ADF-R	AACTCCTGAGGCCATGTTCG
HPRT-F	AAGCTTGCTGGTGAAAAGGA
HPRT-R	TTGCGCTCATCTTAGGCTTT
GAPDH-F	GGTATCGTGGAAGGACTCATG
GAPDH-R	ATGCCAGTGAGCTTCCCGTTC
ß1D-Integrin-F	CATCCCAATTGTAGCAGGCG
ß1D-Integrin-R	GAGACCAGCTTTACGTCCATAG
Caveolin-3-F	GACCCCAAGAACATCAATGAGGAC
Caveolin-3-R	AGAAGGAGATACAGGCGAACAGGA
Myomaker-F	CCTGCTGTCTCTCCCAAG
Myomaker-R	AGAACCAGTGGGTCCCTAA
Myomixer-F	GTTAGAACTGGTGAGCAGGAG
Myomixer-R	CCATCGGGAGCAATGGAA
Gadd45-F	CTGCTGTGAGAACGACATTGA
Gadd45-R	CTCTCCTCGCAGAACAAACTG

sgRNA oligos

**oligonucleotide name**	**oligonucleotide sequence**
sgRNA-mCfl1.1	CCGGAAACATGGTGAGCATT
sgRNA-mCfl1.2	AGCGCCGTCATTTCCCTGGA
sgRNA-mCfl2.1	TATTAAGGACCGCTCGACGC
sgRNA-mCfl2.2	TGGCATCGTACAAAGCATAT
sgRNA-mADF1	ATGTTCGCTGGGGCGGACTT
sgRNA-mADF2	ACTGACTGGCGGTCTACACA

shRNA oligos

**oligonucleotide name**	**oligonucleotide sequence**
shRNA-mCfl-1.1	GCACTCTATGATGCAACCTAT
shRNA-mCfl-1.2	GCGCAAGTCTTCAACACCAGAA
shRNA-mCfl-1.3	GCCAGACAAGGACTGCCGCTAT
shRNA-mCfl-2.1	TCTAAGAAAGAAGACCTAGTA
shRNA-mCfl-2.2	GTAAATGGCTTGGACGATATT
shRNA-mCfl-2.3	TCTACACAGGAGGAGATCAAA
shRNA-mADF-1	CCAGGTATAAAGCATGAGTAT
shRNA-mADF-2	CCACCATAACTGATCCTTTCA

1. Chen, I.H.B., et al., *Nuclear envelope transmembrane proteins (NETs) that are up-regulated during myogenesis.* BMC Cell Biology, 2006. **7**.
2. Tomczak, K.K., et al., *Expression profiling and identification of novel genes involved in myogenic differentiation.* Faseb j, 2004. **18**(2): p. 403-5.
