# Supplementary material for: Dosage-sensitive regulation of Cofilin-1 and LIMK signaling controls myoblast differentiation and fusion
Source: Cell Mol Life Sci. 2026 Sep 18;83(1):338. doi: 10.1007/s00018-026-06437-1 (PMC13589735; doi:10.1007/s00018-026-06437-1)
Supplement: Supplementary file 2 [file 18_2026_6437_MOESM2_ESM.pdf]

Figure 1 c, d

Uncropped blot membrane

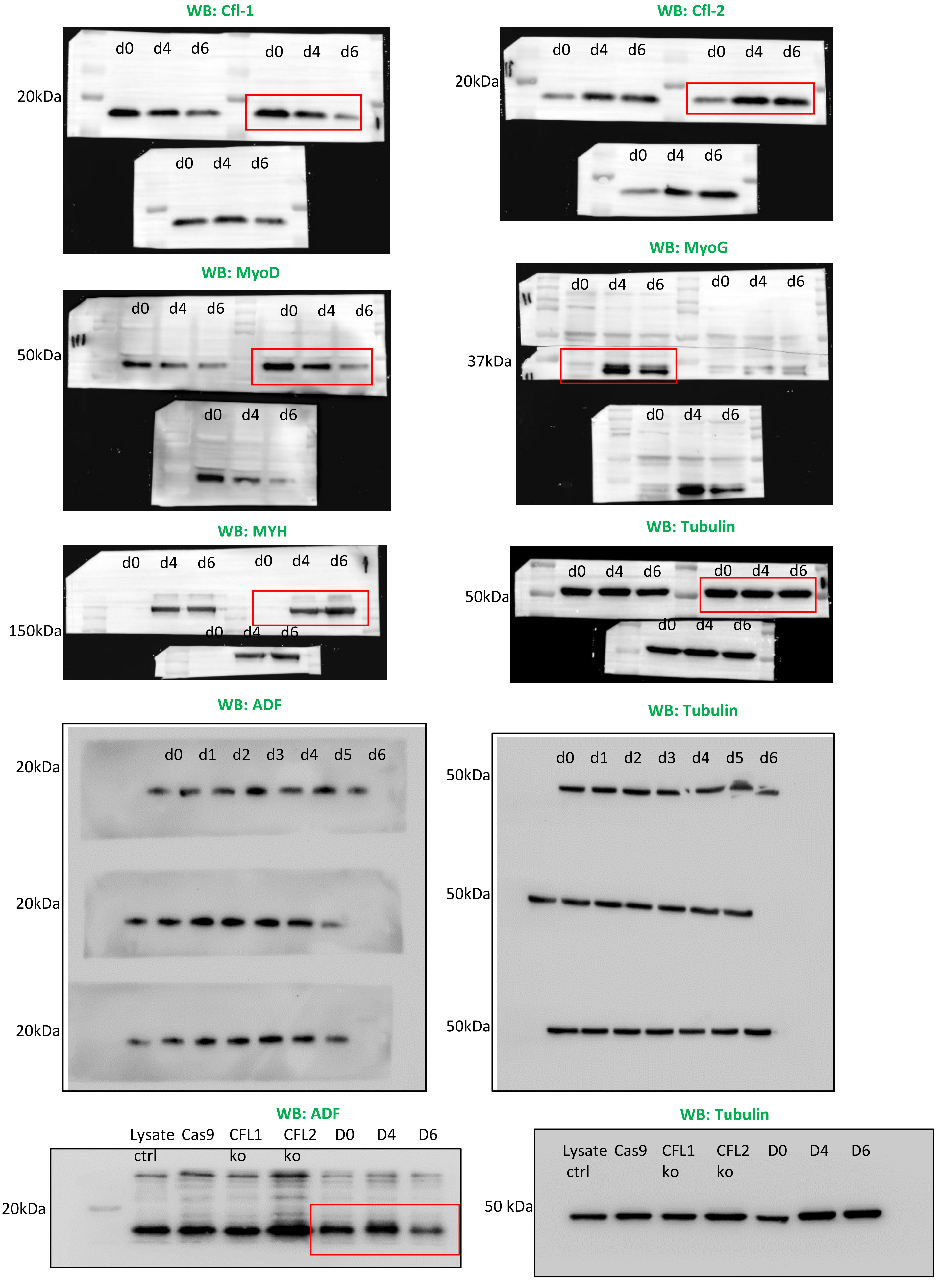

Figure 2 a, b

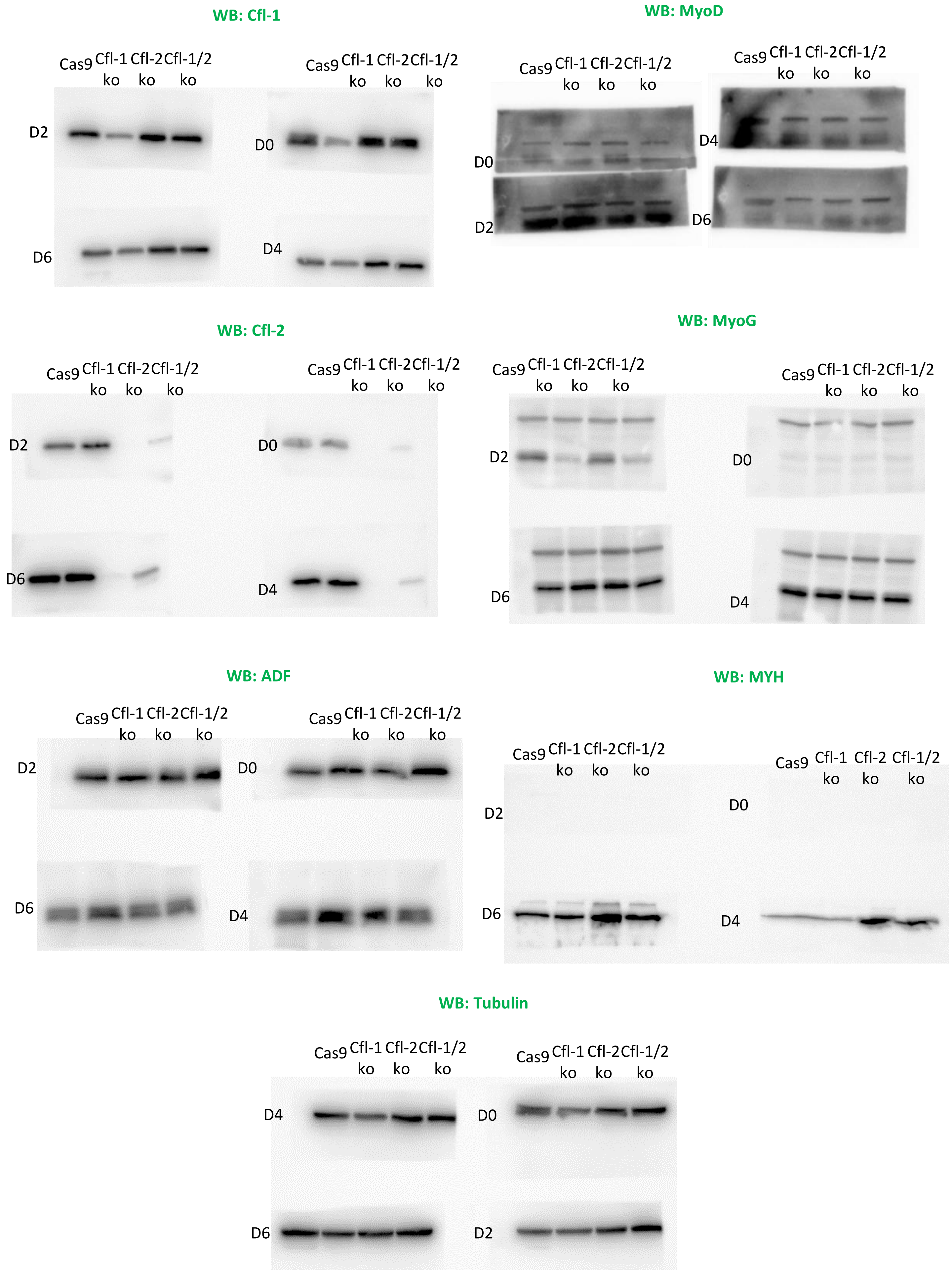

WB: Cfl-1
Cas9 Cfl-1 ko Cfl-2 ko Cfl-1/2 ko
D2
D6
Cas9 Cfl-1 ko Cfl-2 ko Cfl-1/2 ko
D0
D4
WB: MyoD
Cas9 Cfl-1 ko Cfl-2 ko Cfl-1/2 ko
D0
D2
Cas9 Cfl-1 ko Cfl-2 ko Cfl-1/2 ko
D4
D6
WB: Cfl-2
Cas9 Cfl-1 ko Cfl-2 ko Cfl-1/2 ko
D2
D6
Cas9 Cfl-1 ko Cfl-2 ko Cfl-1/2 ko
D0
D4
WB: MyoG
Cas9 Cfl-1 ko Cfl-2 ko Cfl-1/2 ko
D2
D6
Cas9 Cfl-1 ko Cfl-2 ko Cfl-1/2 ko
D0
D4
WB: ADF
Cas9 Cfl-1 ko Cfl-2 ko Cfl-1/2 ko
D2
D6
Cas9 Cfl-1 ko Cfl-2 ko Cfl-1/2 ko
D0
D4
WB: MYH
Cas9 Cfl-1 ko Cfl-2 ko Cfl-1/2 ko
D2
D6
Cas9 Cfl-1 ko Cfl-2 ko Cfl-1/2 ko
D0
D4
WB: Tubulin
Cas9 Cfl-1 ko Cfl-2 ko Cfl-1/2 ko
D4
D6
Cas9 Cfl-1 ko Cfl-2 ko Cfl-1/2 ko
D0
D2

Figure 2 a, b

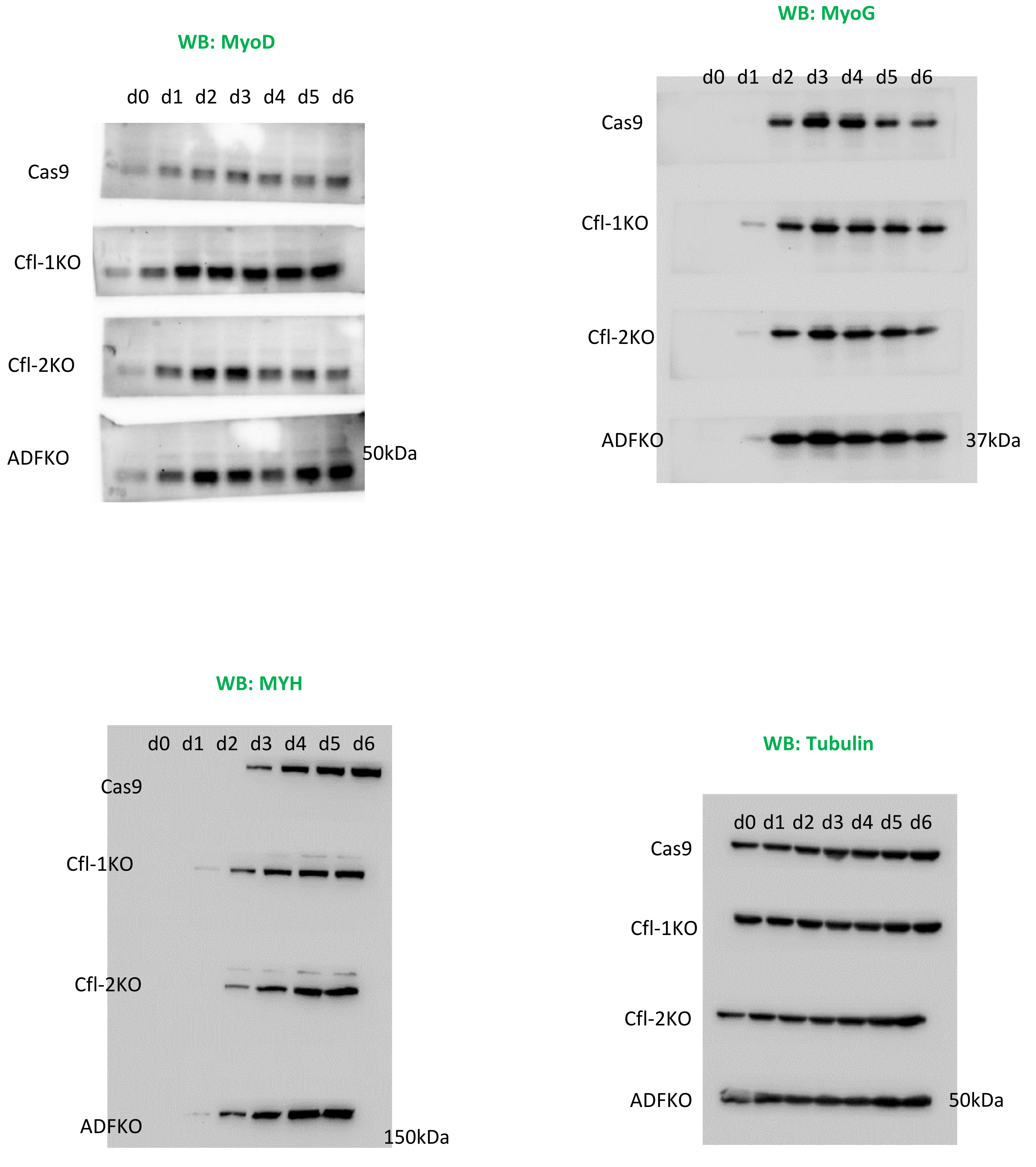
WB: MyoD
d0 d1 d2 d3 d4 d5 d6
Cas9
Cfl-1KO
Cfl-2KO
ADFKO
50kDa
WB: MyoG
d0 d1 d2 d3 d4 d5 d6
Cas9
Cfl-1KO
Cfl-2KO
ADFKO
37kDa
WB: MYH
d0 d1 d2 d3 d4 d5 d6
Cas9
Cfl-1KO
Cfl-2KO
ADFKO
150kDa
WB: Tubulin
d0 d1 d2 d3 d4 d5 d6
Cas9
Cfl-1KO
Cfl-2KO
ADFKO
50kDa

Figure 2 a, b

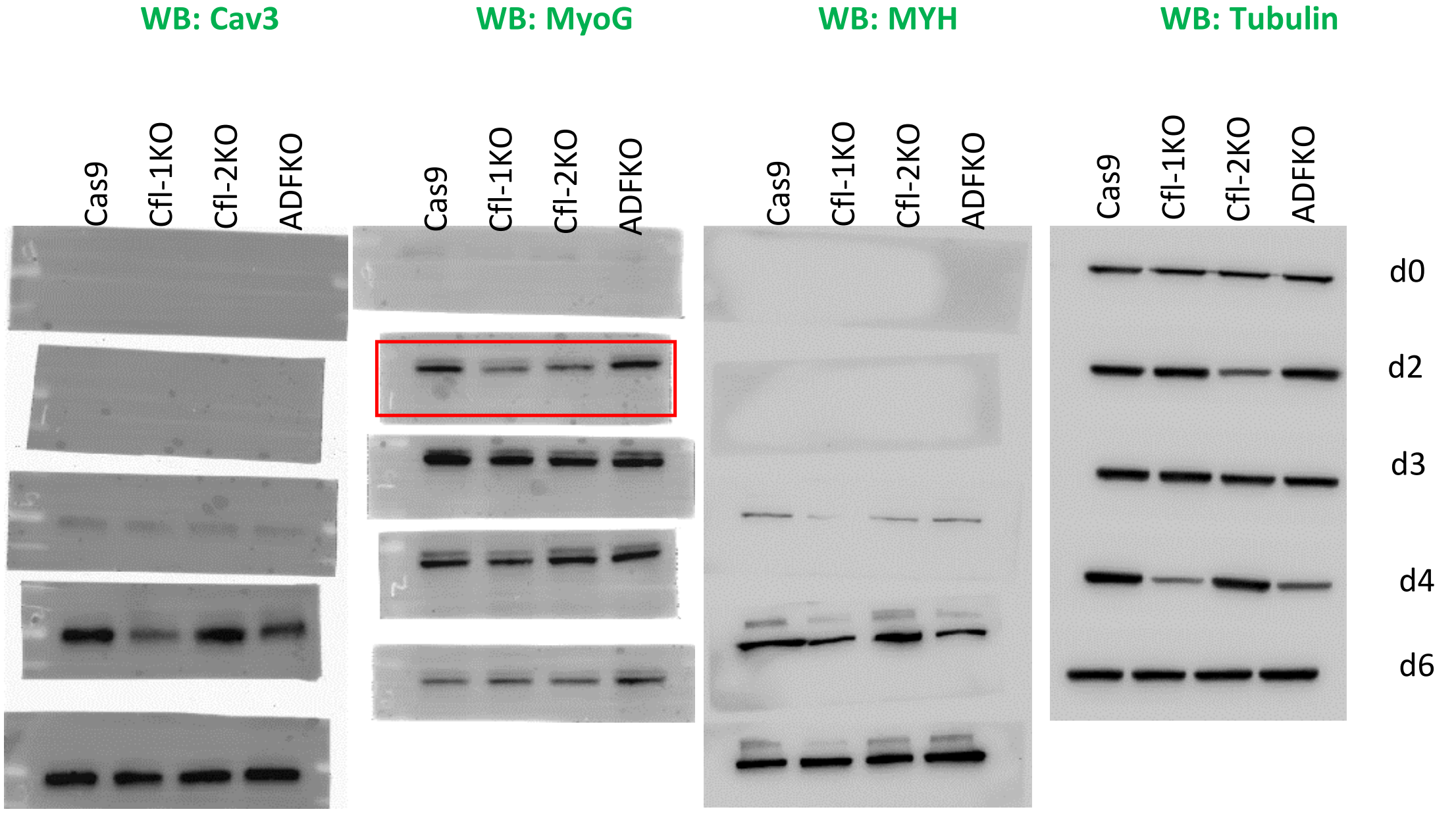

WB: Cav3
WB: MyoG
WB: MYH
WB: Tubulin
Cas9
Cfl-1KO
Cfl-2KO
ADFKO
d0
d2
d3
d4
d6

Figure 2 a, b

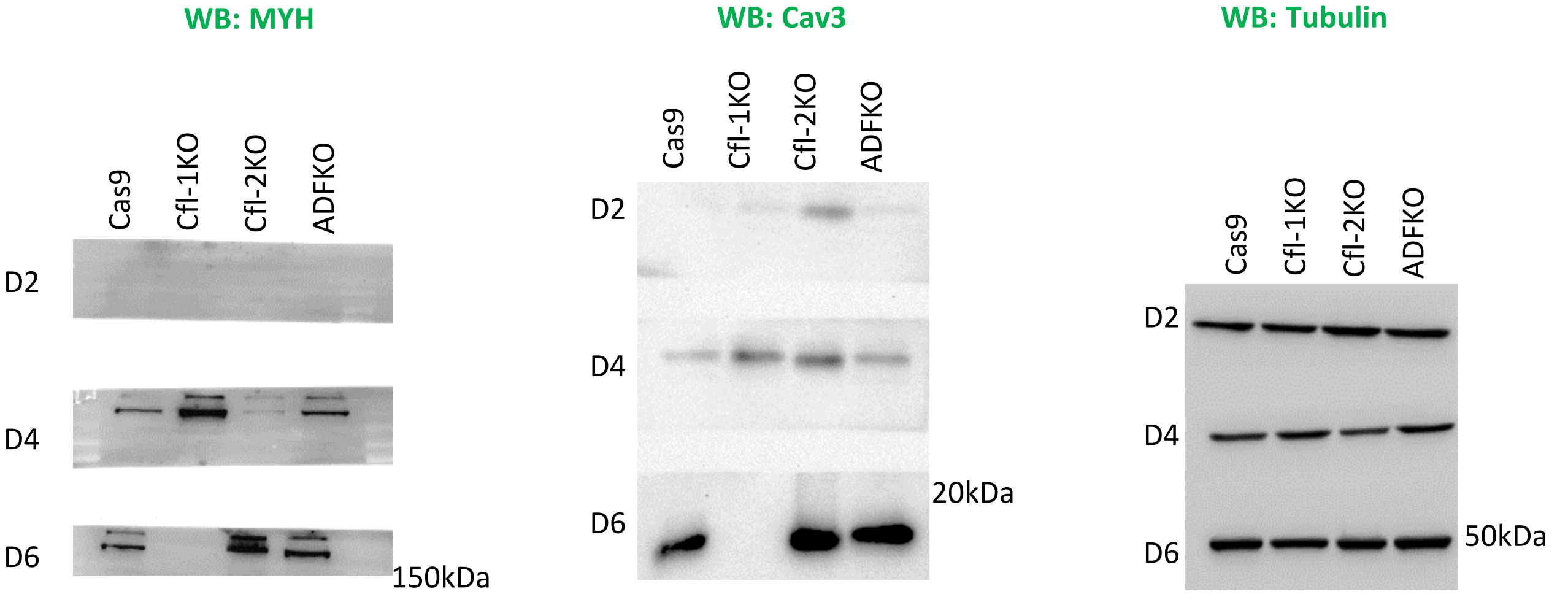
WB: MYH
Cas9
Cfl-1KO
Cfl-2KO
ADFKO
D2
D4
D6
150kDa
WB: Cav3
Cas9
Cfl-1KO
Cfl-2KO
ADFKO
D2
D4
D6
20kDa
WB: Tubulin
Cas9
Cfl-1KO
Cfl-2KO
ADFKO
D2
D4
D6
50kDa

Figure 2 a, b

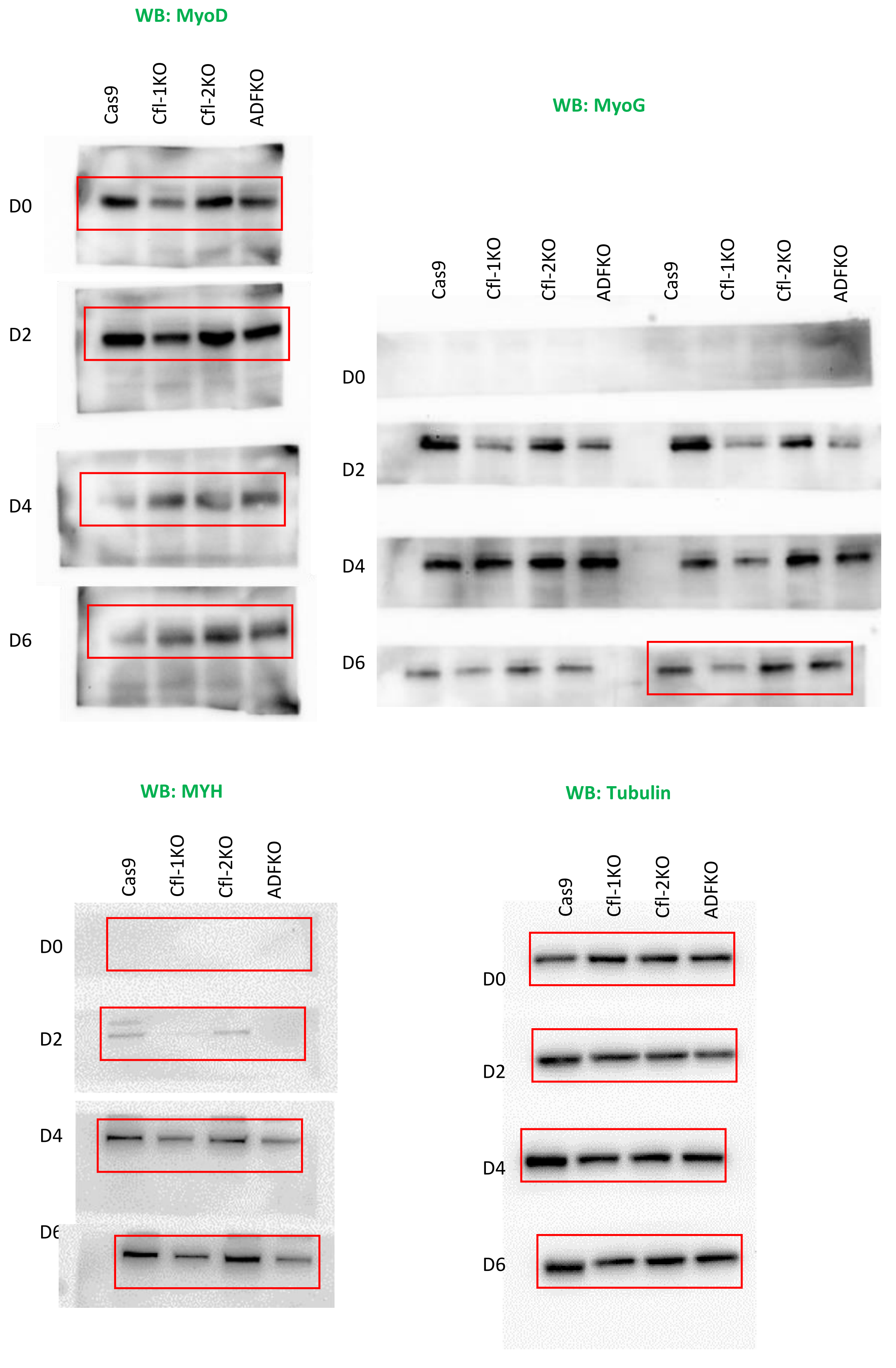
WB: MyoD
Cas9
Cfl-1KO
Cfl-2KO
ADFKO
D0
D2
D4
D6
WB: MyoG
Cas9
Cfl-1KO
Cfl-2KO
ADFKO
Cas9
Cfl-1KO
Cfl-2KO
ADFKO
D0
D2
D4
D6
WB: MYH
Cas9
Cfl-1KO
Cfl-2KO
ADFKO
D0
D2
D4
D6
WB: Tubulin
Cas9
Cfl-1KO
Cfl-2KO
ADFKO
D0
D2
D4
D6

Figure 2 a, b

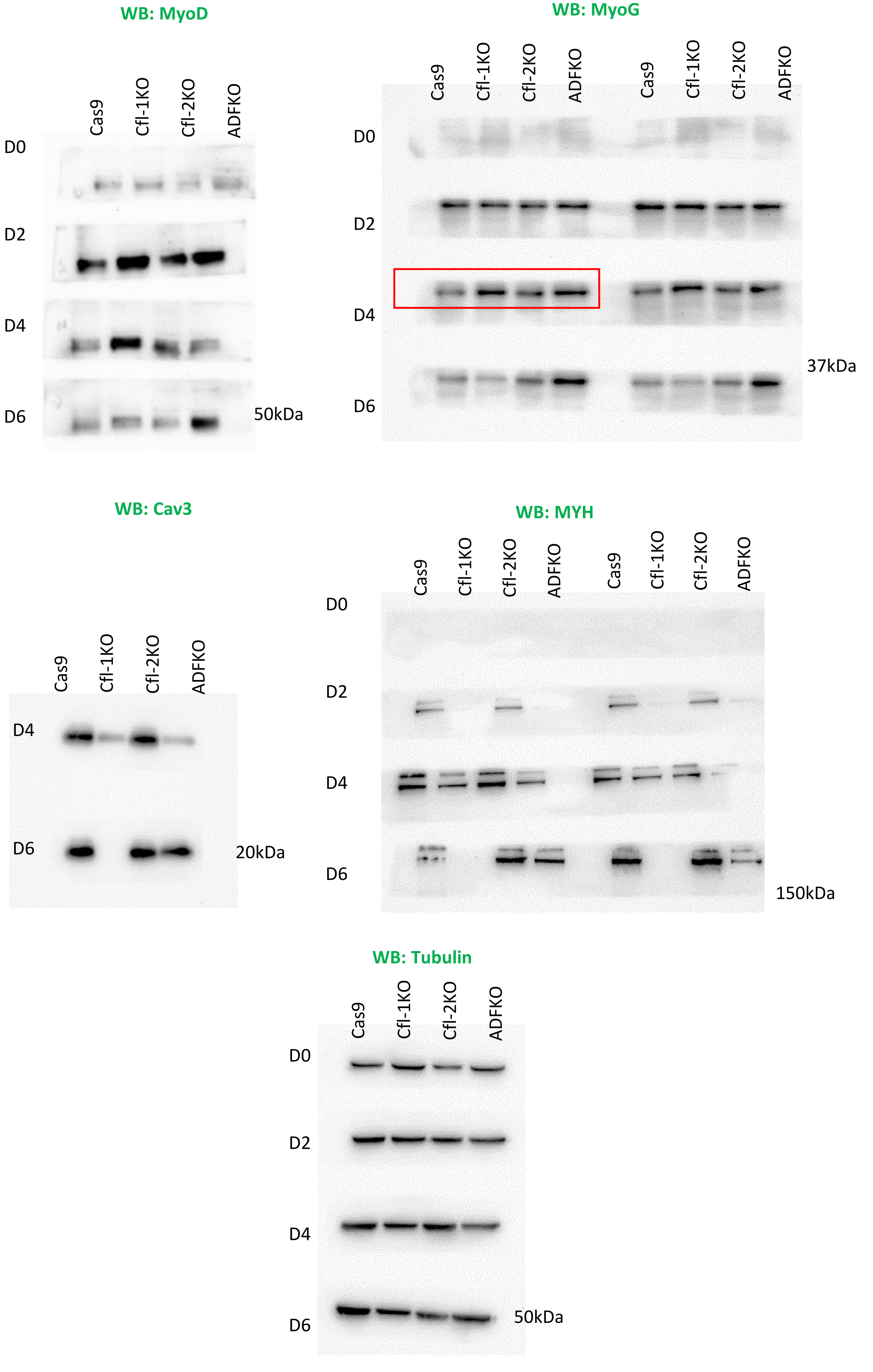
WB: MyoD
Cas9
Cfl-1KO
Cfl-2KO
ADFKO
D0
D2
D4
D6
50kDa
WB: MyoG
Cas9
Cfl-1KO
Cfl-2KO
ADFKO
Cas9
Cfl-1KO
Cfl-2KO
ADFKO
D0
D2
D4
D6
37kDa
WB: Cav3
Cas9
Cfl-1KO
Cfl-2KO
ADFKO
D4
D6
20kDa
WB: MYH
Cas9
Cfl-1KO
Cfl-2KO
ADFKO
Cas9
Cfl-1KO
Cfl-2KO
ADFKO
D0
D2
D4
D6
150kDa
WB: Tubulin
Cas9
Cfl-1KO
Cfl-2KO
ADFKO
D0
D2
D4
D6
50kDa

Figure 2 a, b

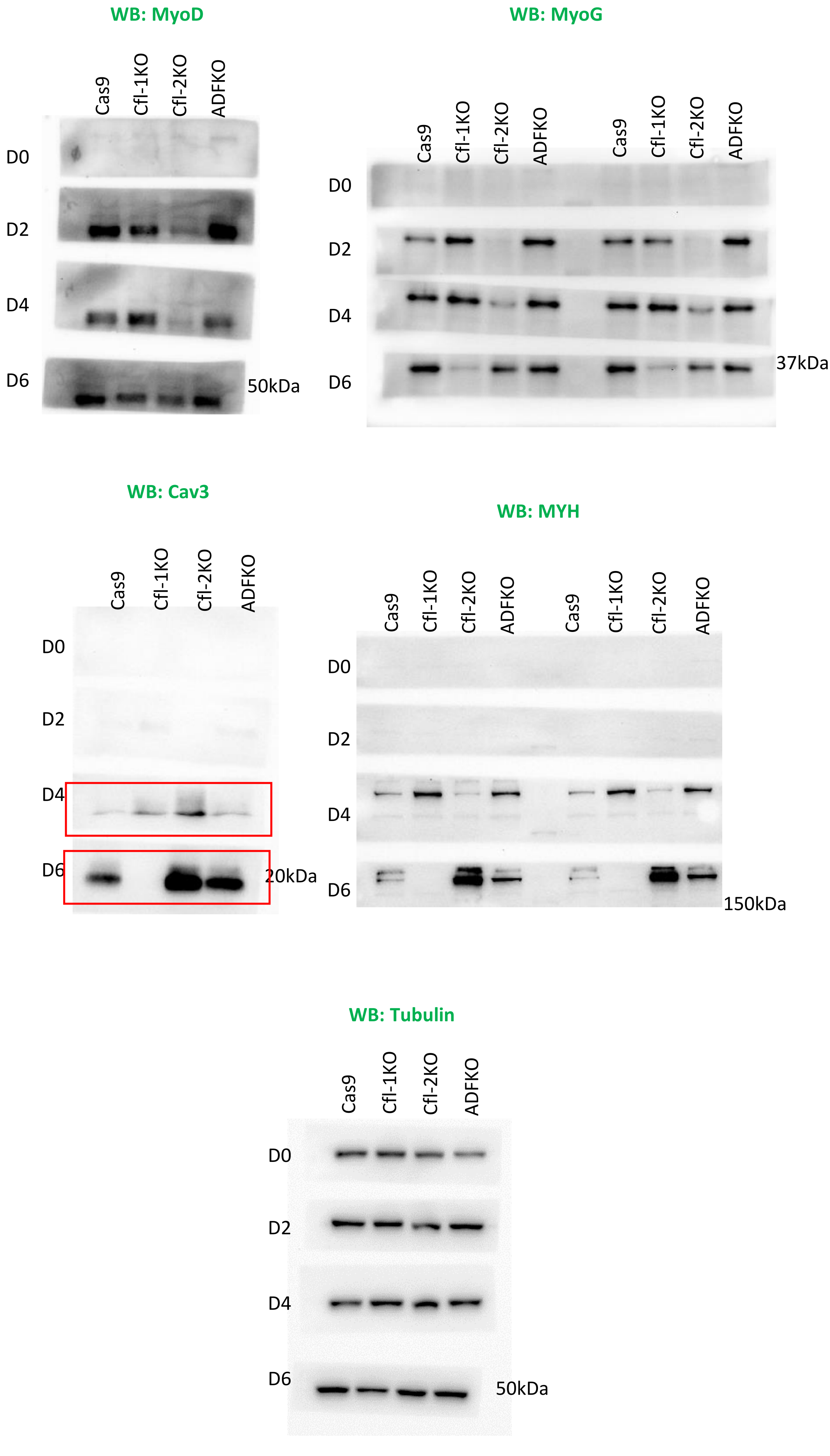
WB: MyoD
Cas9
Cfl-1KO
Cfl-2KO
ADFKO
D0
D2
D4
D6
50kDa
WB: MyoG
Cas9
Cfl-1KO
Cfl-2KO
ADFKO
Cas9
Cfl-1KO
Cfl-2KO
ADFKO
D0
D2
D4
D6
37kDa
WB: Cav3
Cas9
Cfl-1KO
Cfl-2KO
ADFKO
D0
D2
D4
D6
20kDa
WB: MYH
Cas9
Cfl-1KO
Cfl-2KO
ADFKO
Cas9
Cfl-1KO
Cfl-2KO
ADFKO
D0
D2
D4
D6
150kDa
WB: Tubulin
Cas9
Cfl-1KO
Cfl-2KO
ADFKO
D0
D2
D4
D6
50kDa

Figure 3 b, c, d

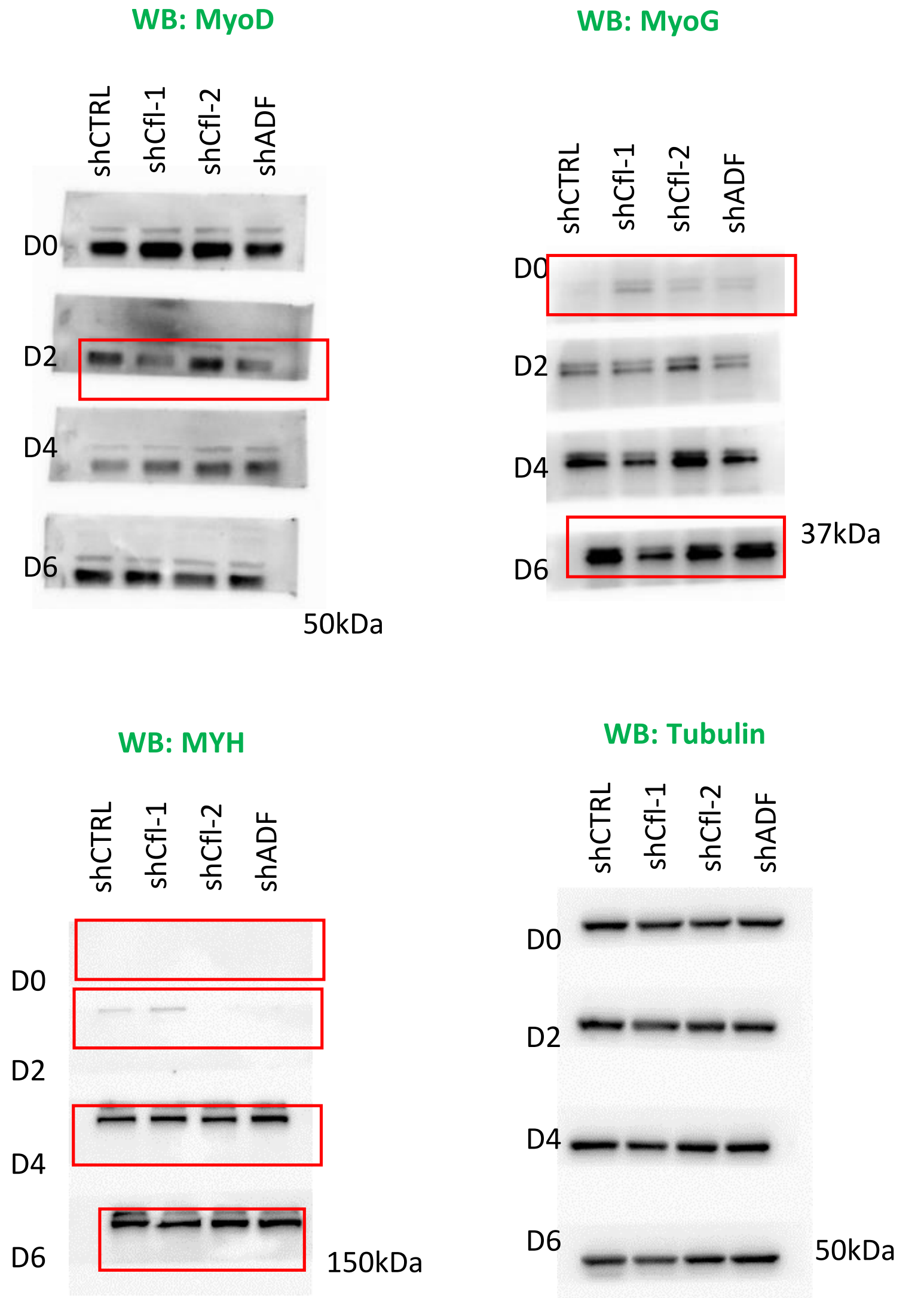

WB: MyoD
shCTRL
shCfl-1
shCfl-2
shADF
D0
D2
D4
D6
50kDa
WB: MyoG
shCTRL
shCfl-1
shCfl-2
shADF
D0
D2
D4
D6
37kDa
WB: MYH
shCTRL
shCfl-1
shCfl-2
shADF
D0
D2
D4
D6
150kDa
WB: Tubulin
shCTRL
shCfl-1
shCfl-2
shADF
D0
D2
D4
D6
50kDa

Figure 3 b, c, d

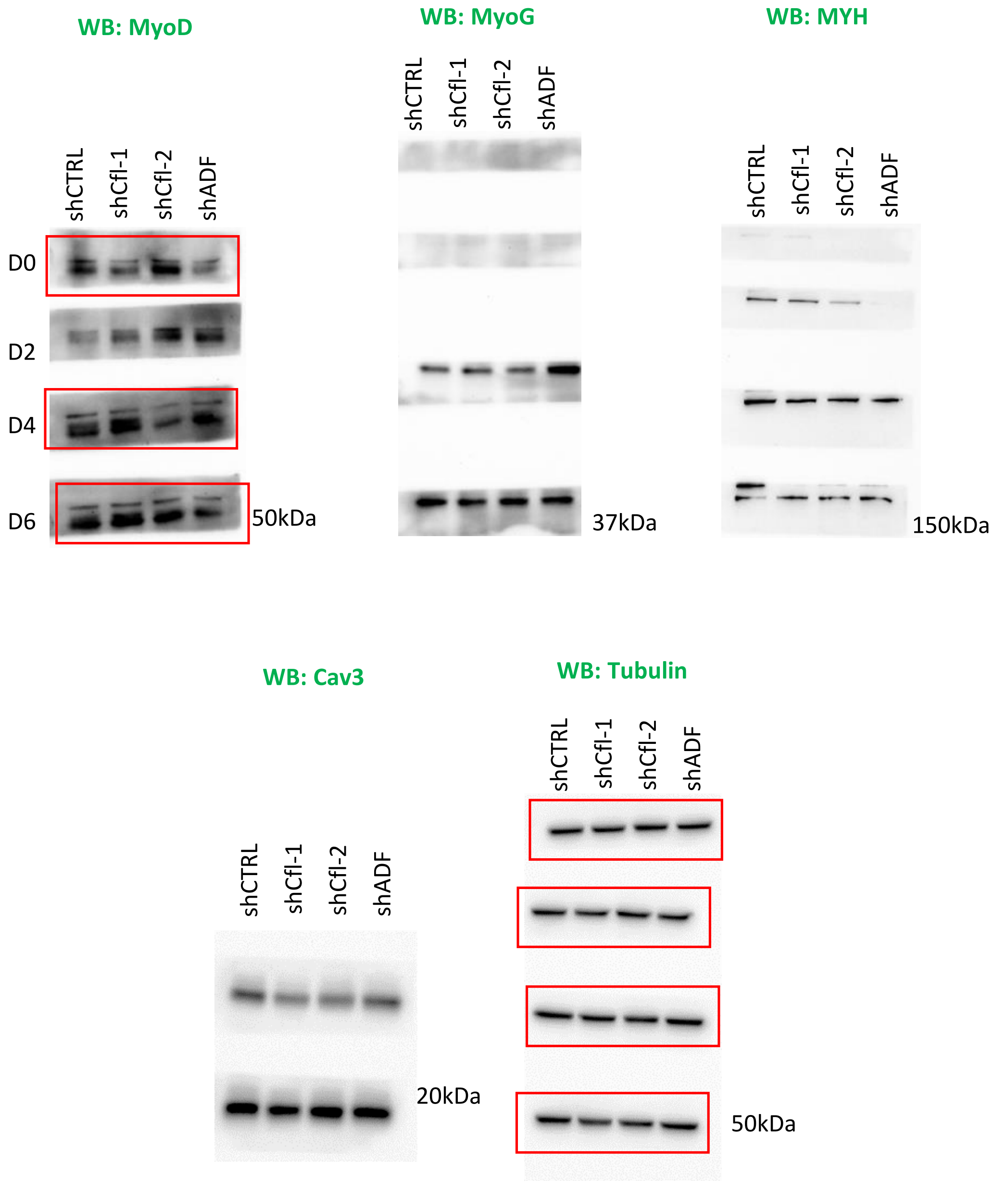
WB: MyoD
shCTRL
shCfl-1
shCfl-2
shADF
D0
D2
D4
D6
50kDa
WB: MyoG
shCTRL
shCfl-1
shCfl-2
shADF
37kDa
WB: MYH
shCTRL
shCfl-1
shCfl-2
shADF
150kDa
WB: Cav3
shCTRL
shCfl-1
shCfl-2
shADF
20kDa
WB: Tubulin
shCTRL
shCfl-1
shCfl-2
shADF
50kDa

Figure 3 b, c, d

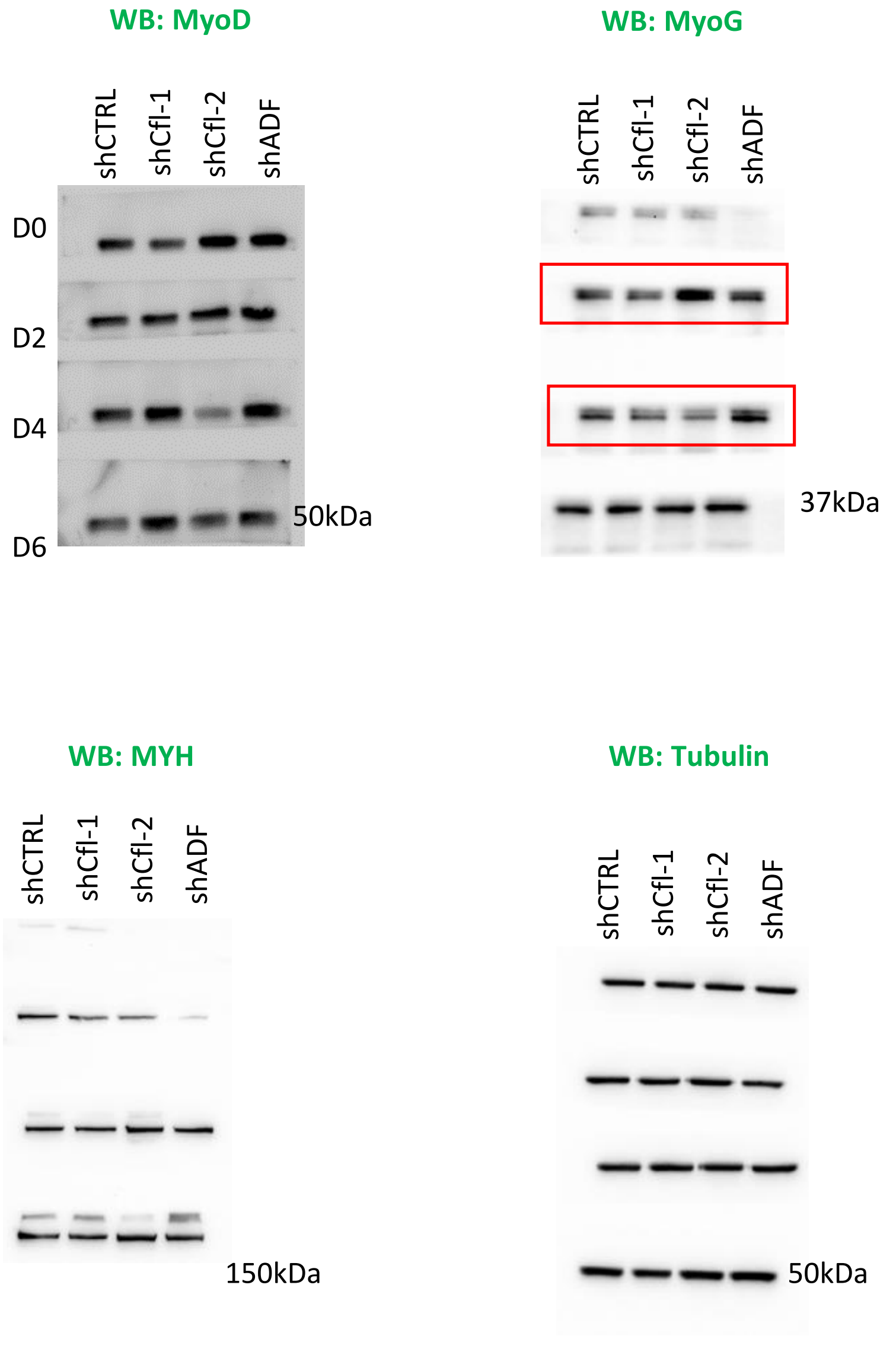
WB: MyoD
shCTRL
shCfl-1
shCfl-2
shADF
D0
D2
D4
D6
50kDa
WB: MyoG
shCTRL
shCfl-1
shCfl-2
shADF
37kDa
WB: MYH
shCTRL
shCfl-1
shCfl-2
shADF
150kDa
WB: Tubulin
shCTRL
shCfl-1
shCfl-2
shADF
50kDa

Figure 3 b, c, d

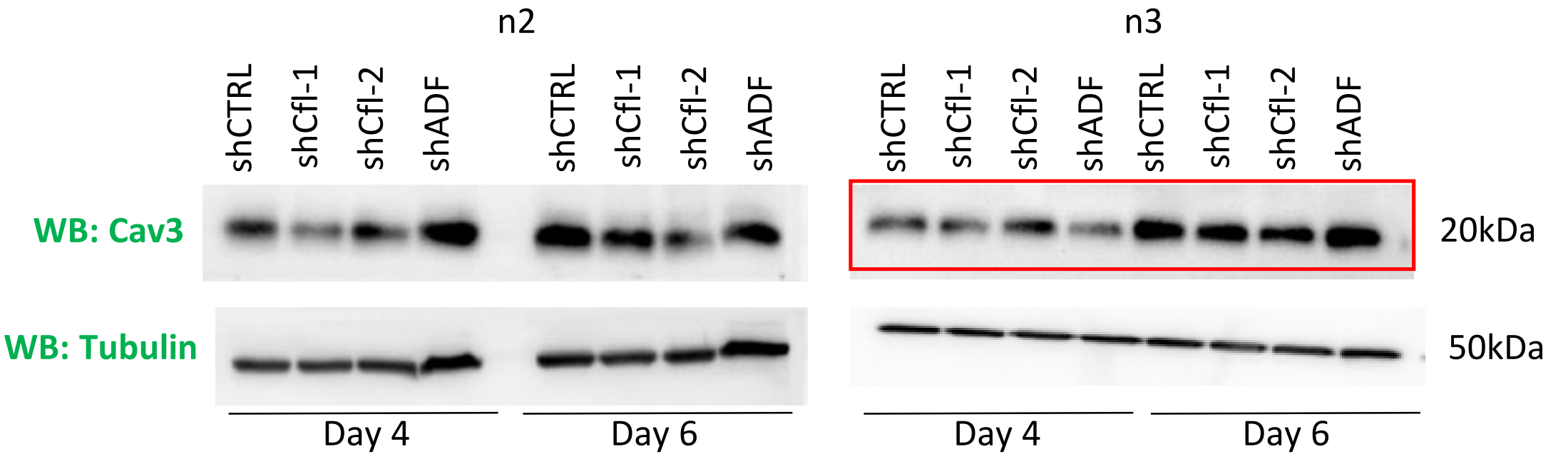

n2
n3
shCTRL
shCfl-1
shCfl-2
shADF
shCTRL
shCfl-1
shCfl-2
shADF
shCTRL
shCfl-1
shCfl-2
shADF
shCTRL
shCfl-1
shCfl-2
shADF
WB: Cav3
WB: Tubulin
20kDa
50kDa
Day 4
Day 6
Day 4
Day 6

Figure 4 c, d

WB: PH3

d2 d4 d6

shCTRL shCfl1 shADF shCTRL shCfl1 shADF shCTRL shCfl1 shADF

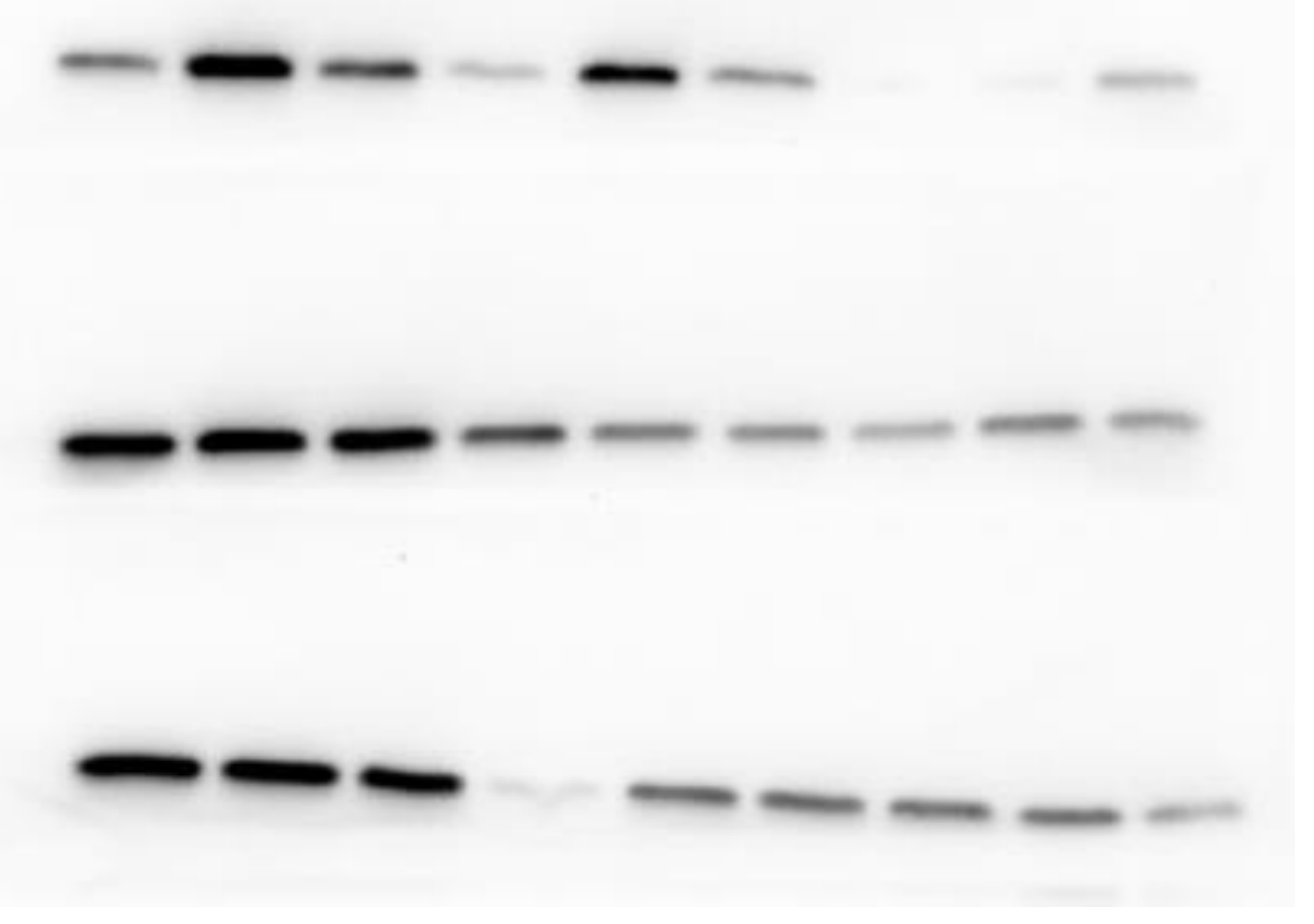

15kDa

WB: Tubulin

d2 d4 d6

shCTRL shCfl1 shADF shCTRL shCfl1 shADF shCTRL shCfl1 shADF

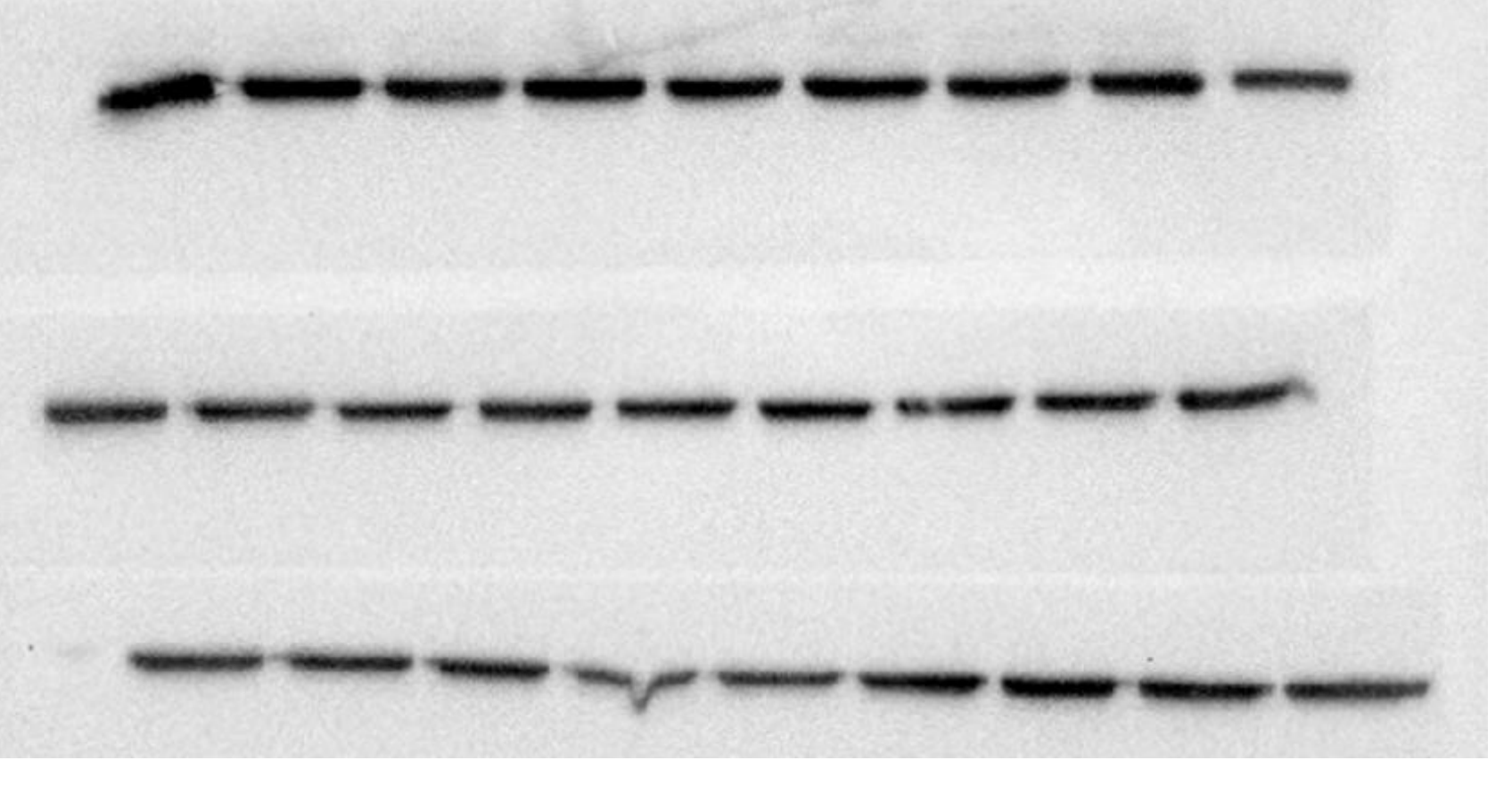

50kDa

Figure 4 c, d

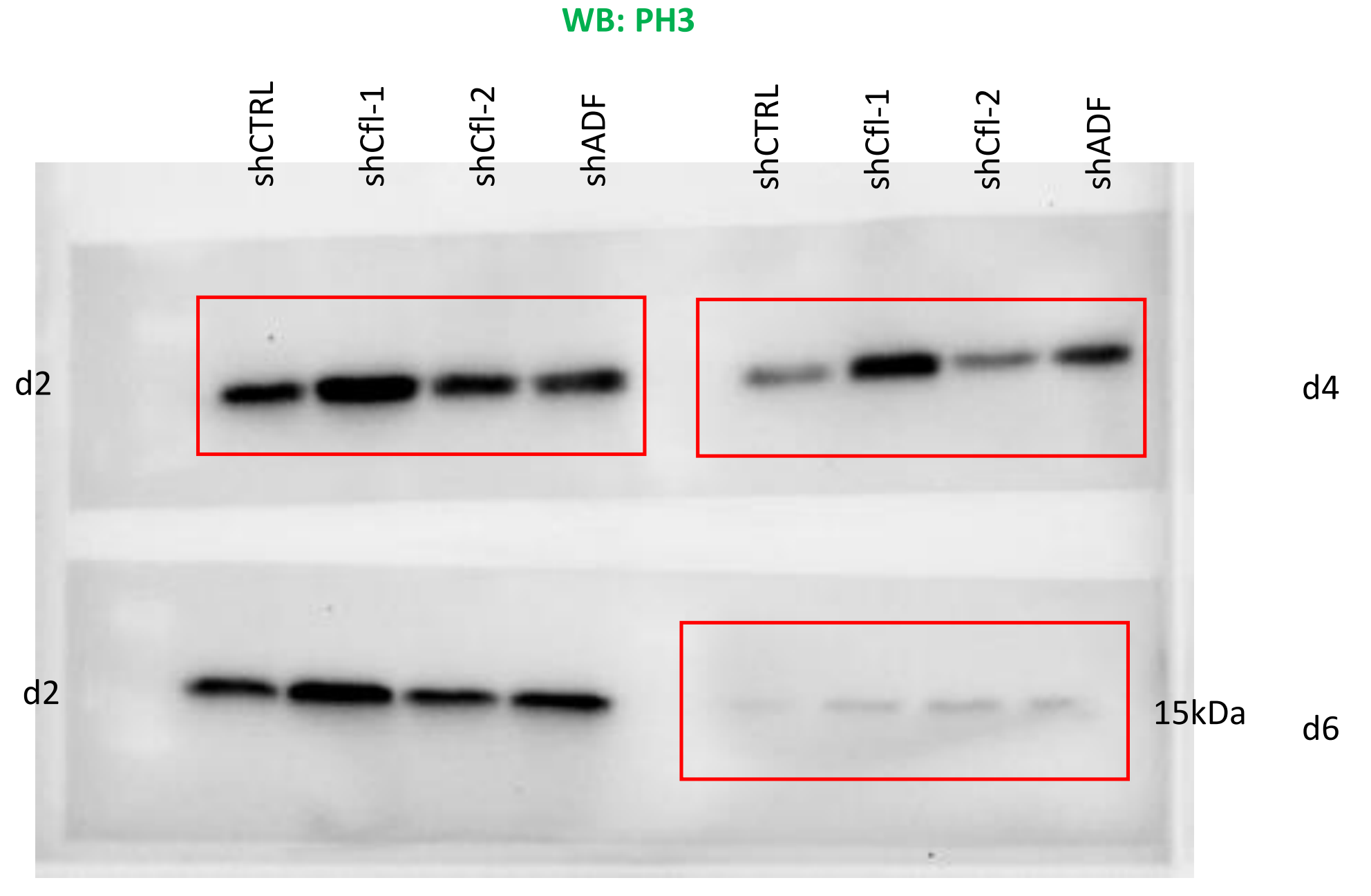
WB: PH3
shCTRL
shCfl-1
shCfl-2
shADF
shCTRL
shCfl-1
shCfl-2
shADF
d2
d4
d2
15kDa
d6

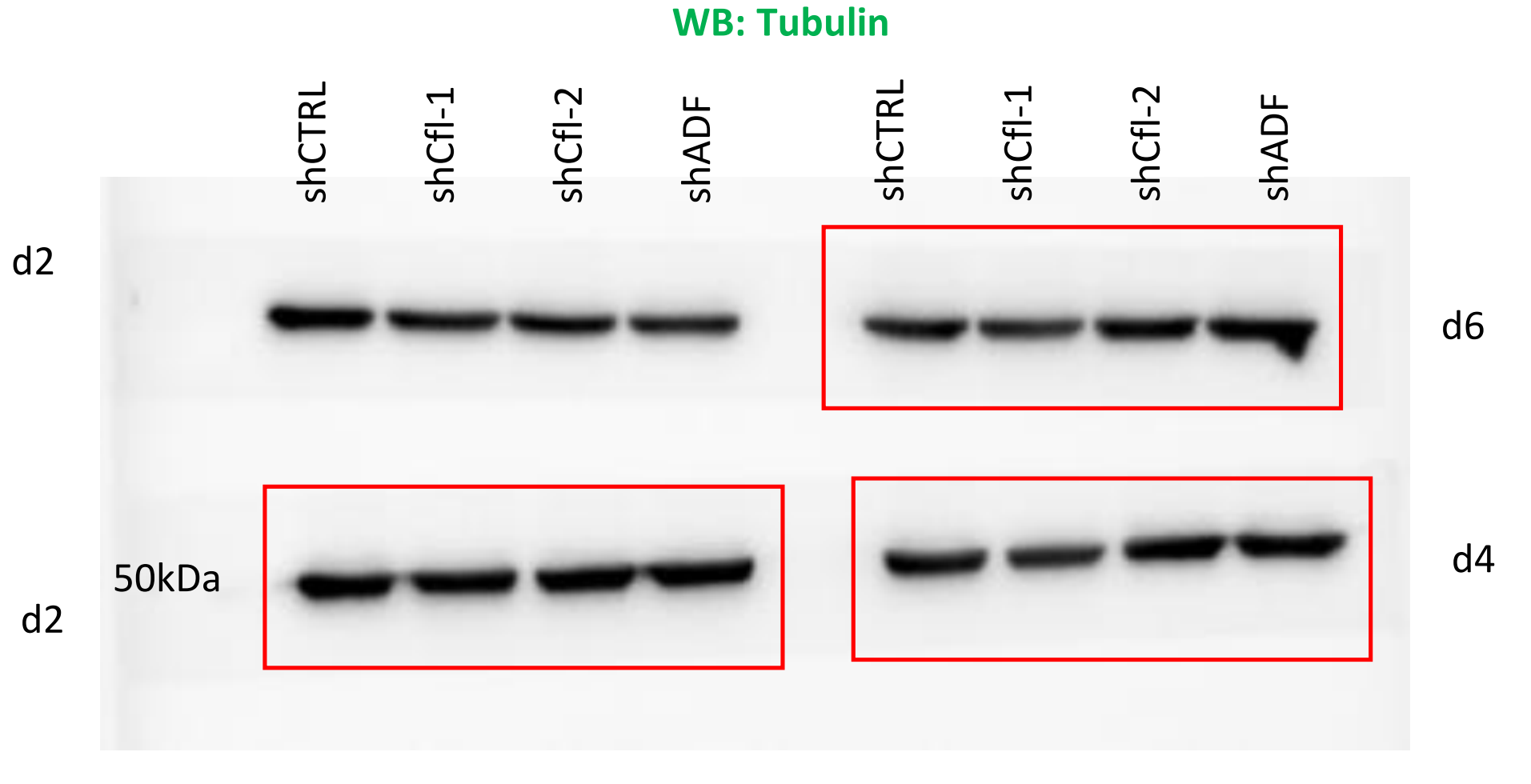
WB: Tubulin
shCTRL
shCfl-1
shCfl-2
shADF
shCTRL
shCfl-1
shCfl-2
shADF
d2
d6
50kDa
d2
d4

Figure 4 c, d

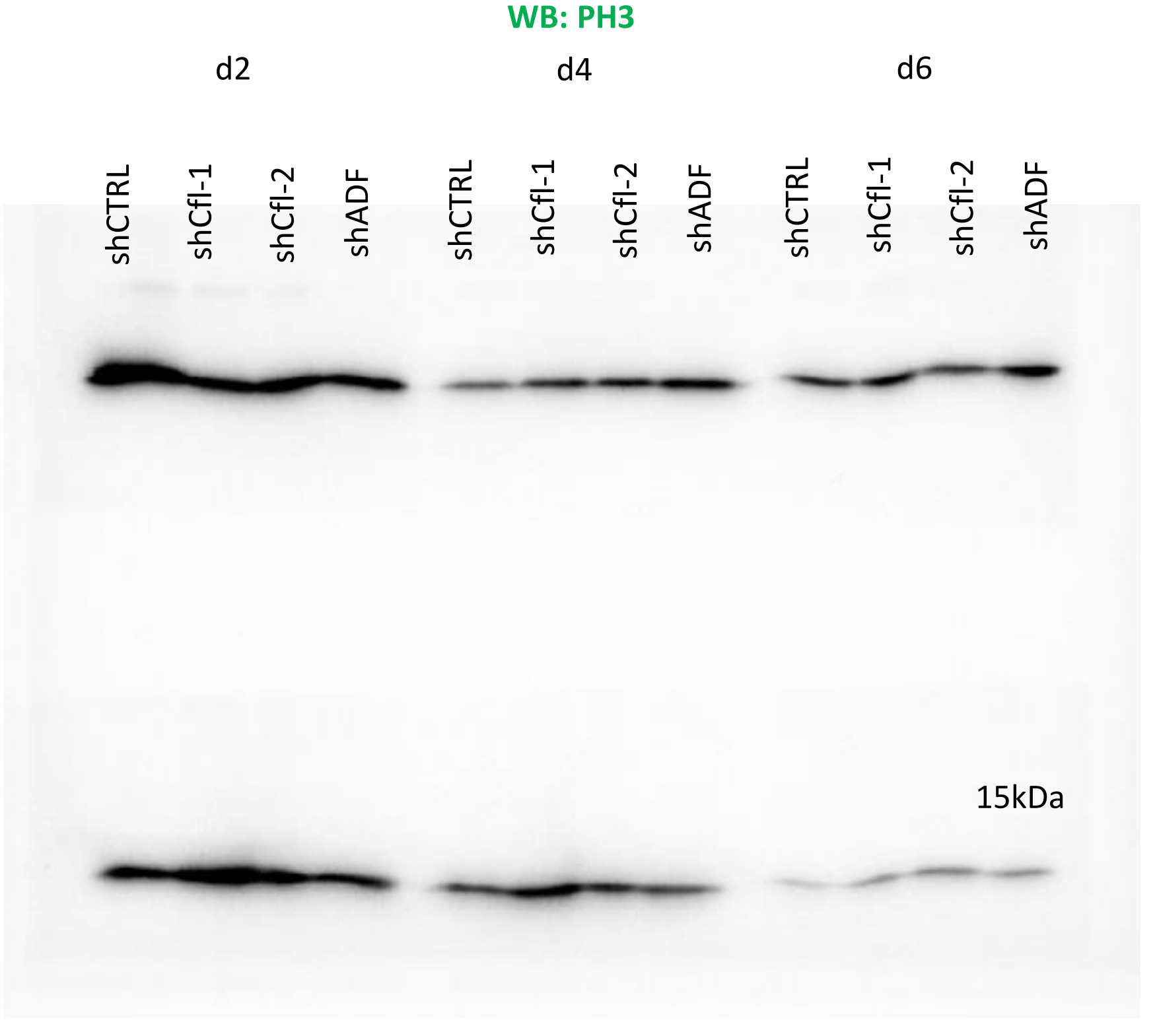
WB: PH3
d2
d4
d6
shCTRL
shCfl-1
shCfl-2
shADF
shCTRL
shCfl-1
shCfl-2
shADF
shCTRL
shCfl-1
shCfl-2
shADF
15kDa

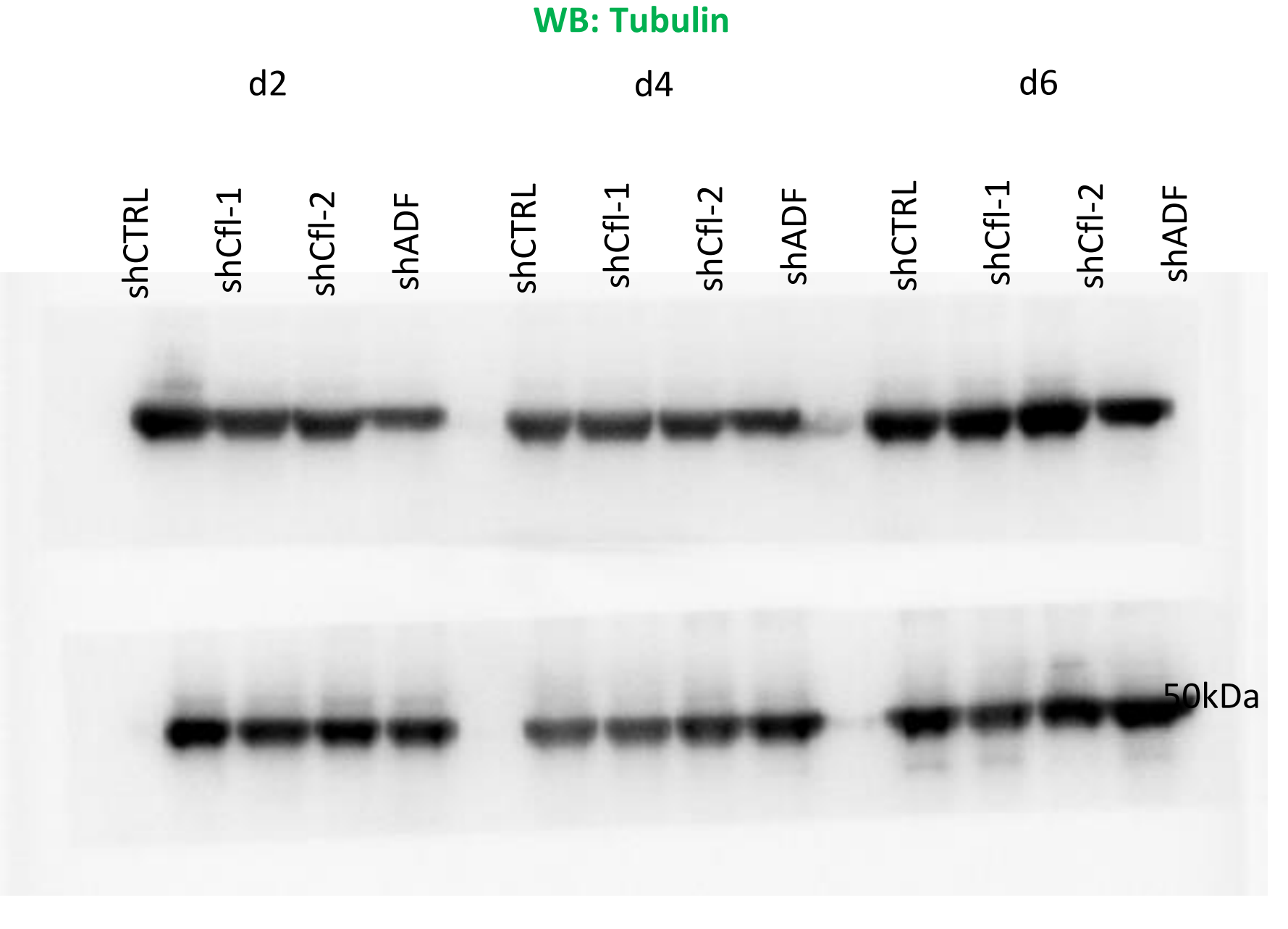
WB: Tubulin
d2
d4
d6
shCTRL
shCfl-1
shCfl-2
shADF
shCTRL
shCfl-1
shCfl-2
shADF
shCTRL
shCfl-1
shCfl-2
shADF
50kDa

Figure 5 c, d

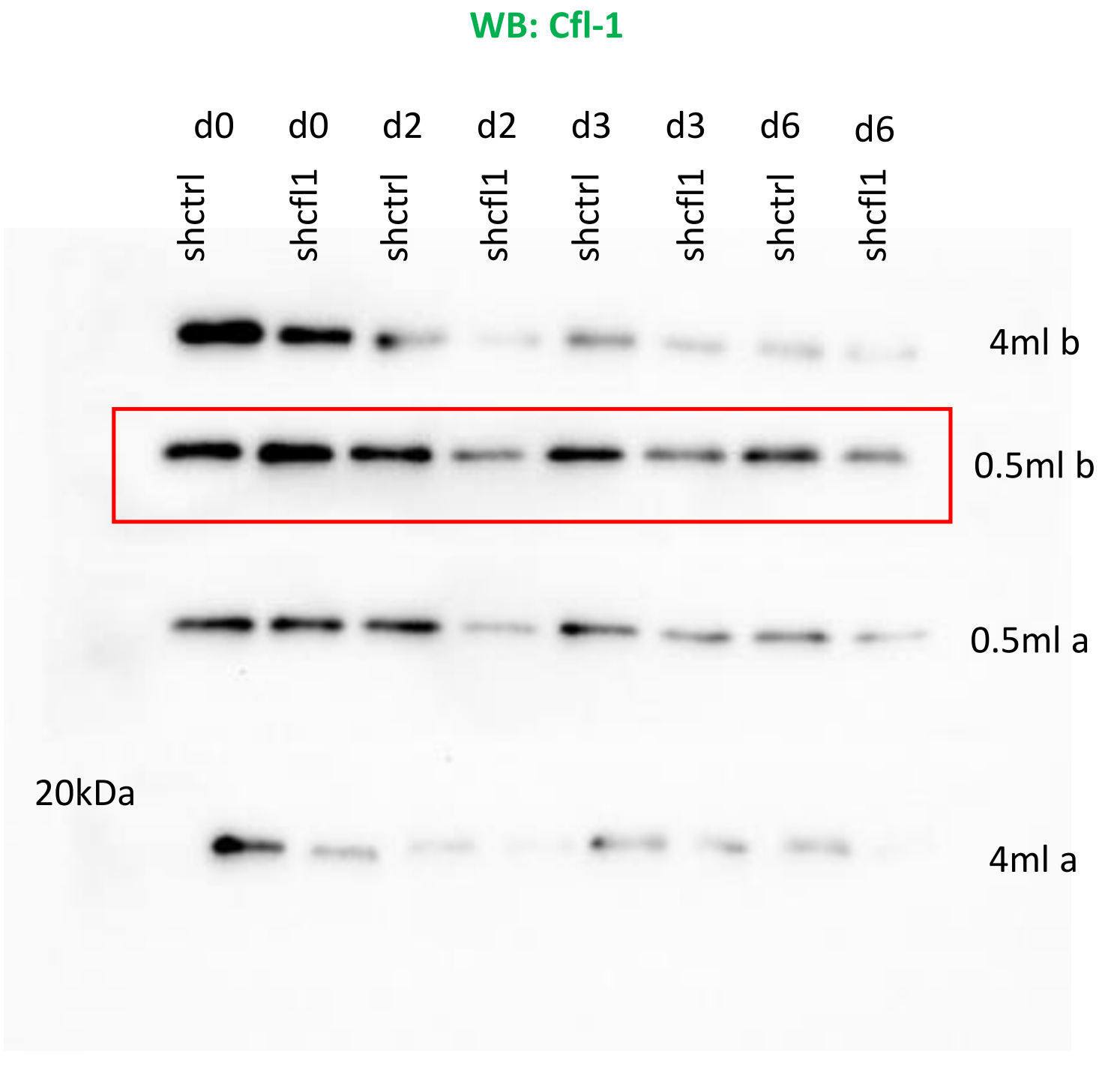
WB: Cfl-1
d0 d0 d2 d2 d3 d3 d6 d6
shctrl shcfl1 shctrl shcfl1 shctrl shcfl1 shctrl shcfl1
4ml b
0.5ml b
0.5ml a
20kDa
4ml a

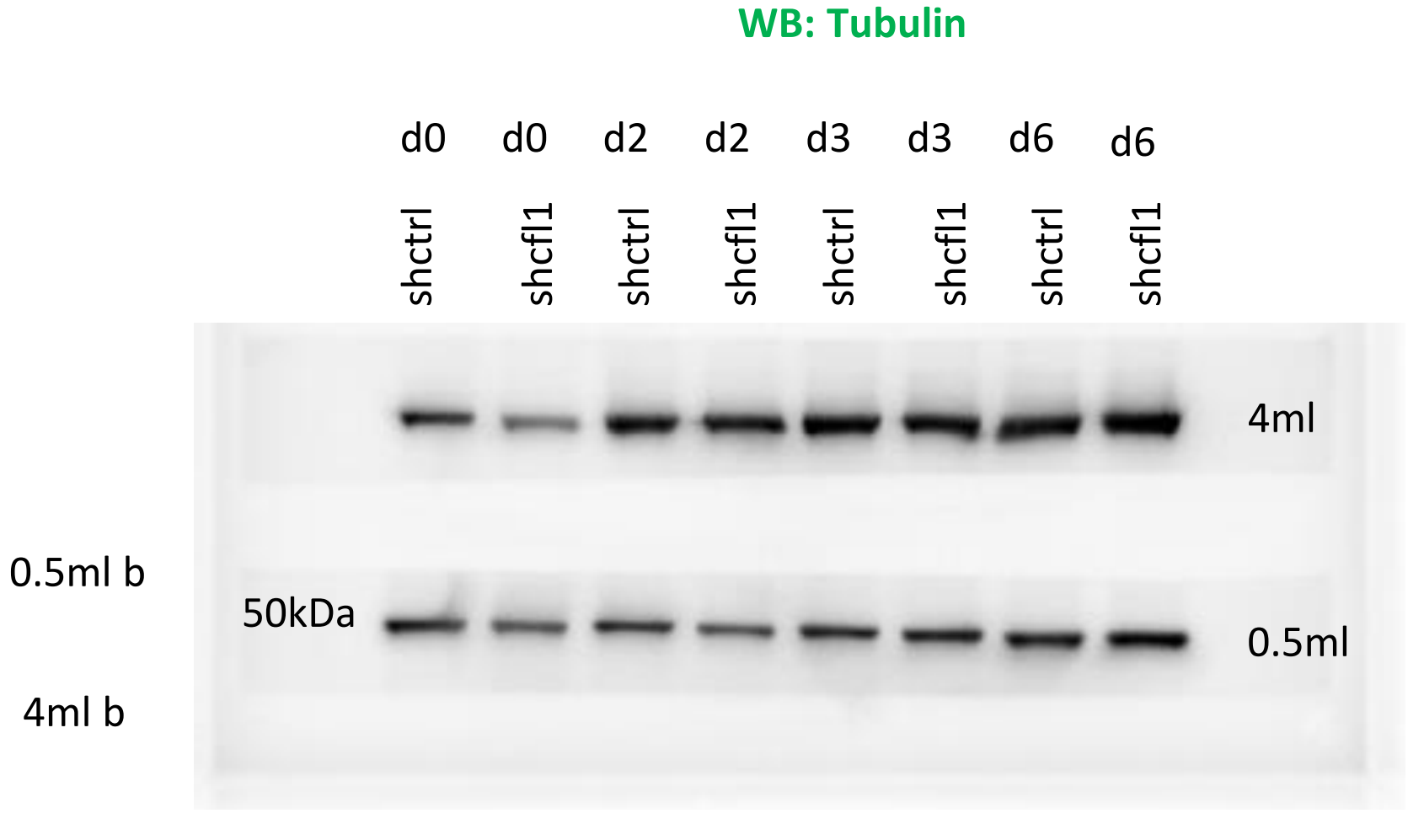
WB: Tubulin
d0 d0 d2 d2 d3 d3 d6 d6
shctrl shcfl1 shctrl shcfl1 shctrl shcfl1 shctrl shcfl1
4ml
0.5ml b
50kDa
4ml b
0.5ml

Figure 5 c, d

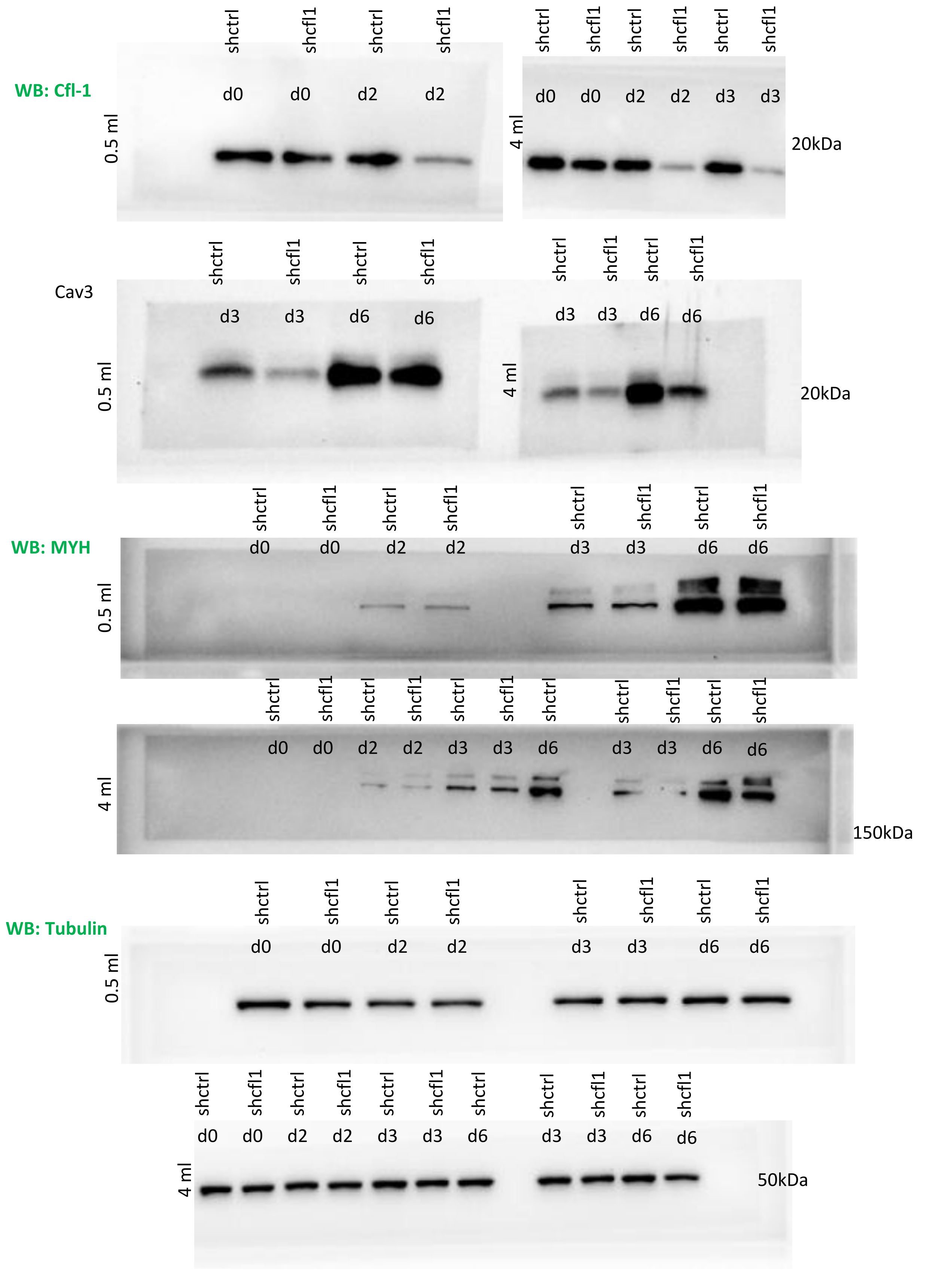
WB: Cfl-1
0.5 ml
shctrl shcfl1 shctrl shcfl1
d0 d0 d2 d2
4 ml
shctrl shcfl1 shctrl shcfl1 shctrl shcfl1
d0 d0 d2 d2 d3 d3
20kDa
Cav3
0.5 ml
shctrl shcfl1 shctrl shcfl1
d3 d3 d6 d6
4 ml
shctrl shcfl1 shctrl shcfl1
d3 d3 d6 d6
20kDa
WB: MYH
0.5 ml
shctrl shcfl1 shctrl shcfl1 shctrl shcfl1 shctrl shcfl1
d0 d0 d2 d2 d3 d3 d6 d6
4 ml
shctrl shcfl1 shctrl shcfl1 shctrl shcfl1 shctrl shctrl shcfl1 shctrl shcfl1
d0 d0 d2 d2 d3 d3 d6 d3 d3 d6 d6
150kDa
WB: Tubulin
0.5 ml
shctrl shcfl1 shctrl shcfl1 shctrl shcfl1 shctrl shcfl1
d0 d0 d2 d2 d3 d3 d6 d6
4 ml
shctrl shcfl1 shctrl shcfl1 shctrl shcfl1 shctrl shctrl shcfl1 shctrl shcfl1
d0 d0 d2 d2 d3 d3 d6 d3 d3 d6 d6
50kDa

Figure 5 c, d

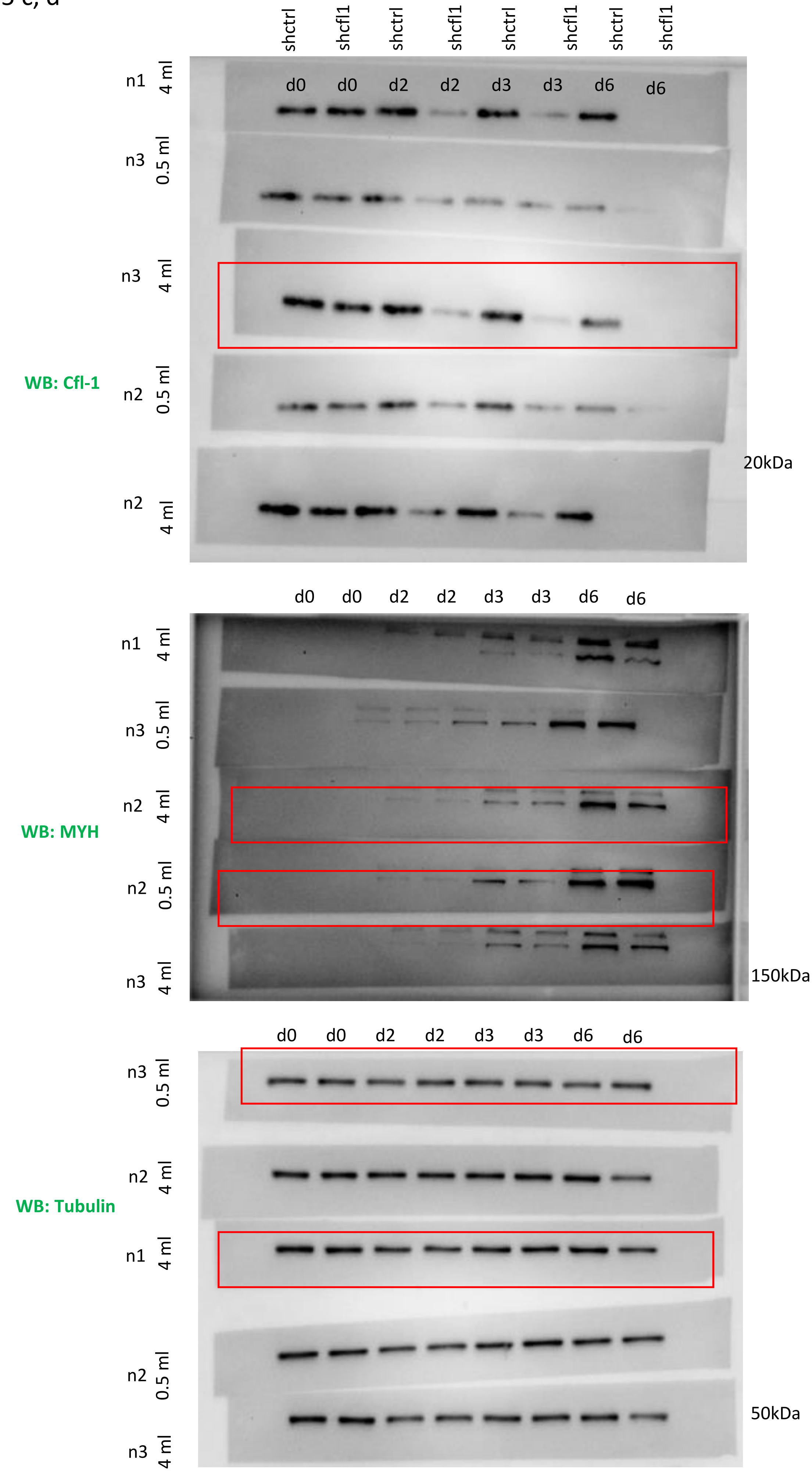

shctrl
shcfl1
shctrl
shcfl1
shctrl
shcfl1
shctrl
shcfl1
d0 d0 d2 d2 d3 d3 d6 d6
n1 4 ml
n3 0.5 ml
n3 4 ml
WB: Cfl-1
n2 0.5 ml
n2 4 ml
20kDa
d0 d0 d2 d2 d3 d3 d6 d6
n1 4 ml
n3 0.5 ml
n2 4 ml
WB: MYH
n2 0.5 ml
n3 4 ml
150kDa
d0 d0 d2 d2 d3 d3 d6 d6
n3 0.5 ml
n2 4 ml
WB: Tubulin
n1 4 ml
n2 0.5 ml
n3 4 ml
50kDa

Figure 6 b, c

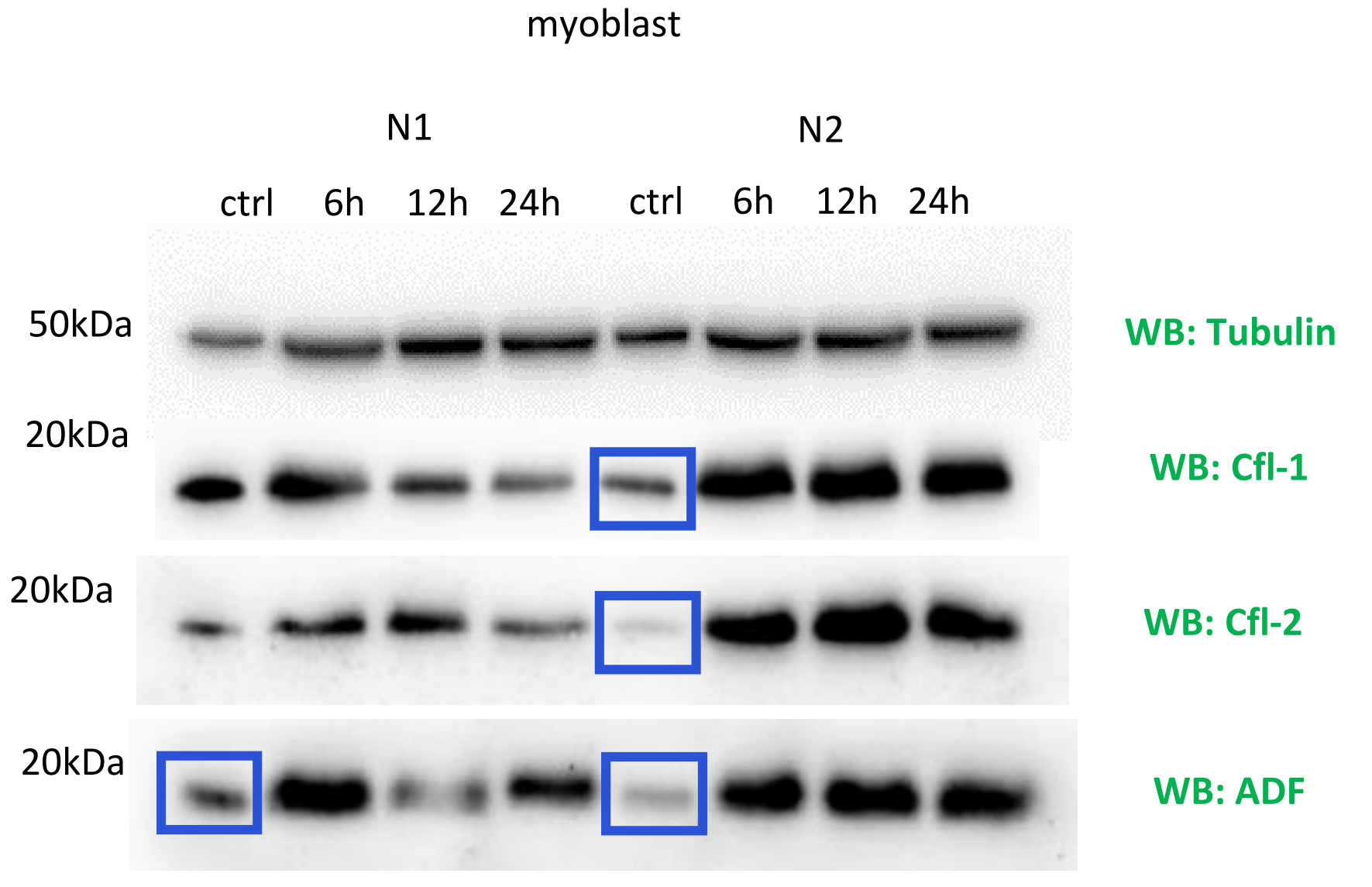

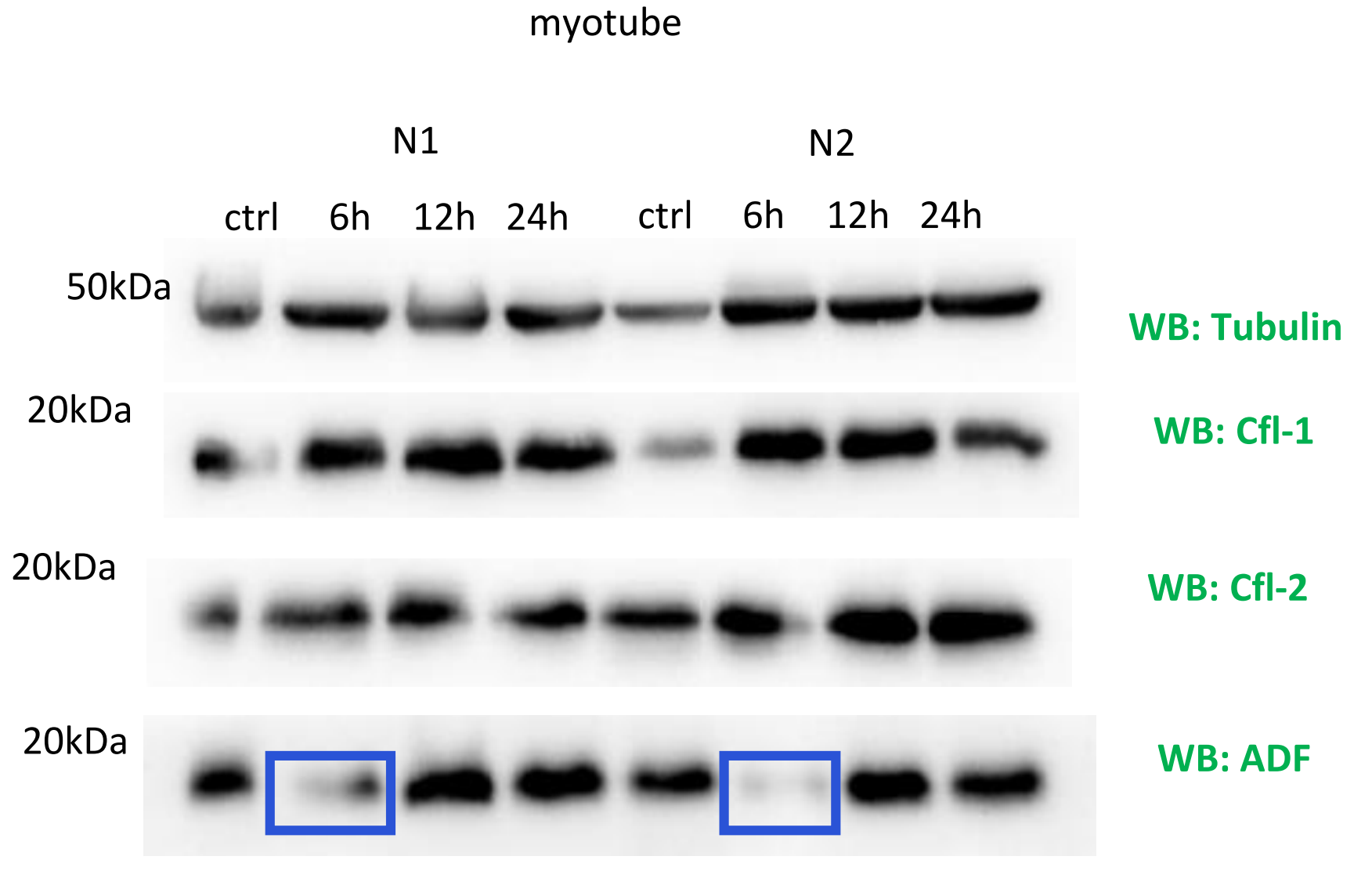

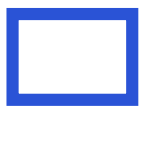

For the Western blot quantification in Figure 6, time points in the cycloheximide chase was excluded from the final analysis due to a technical loading artifact that precluded accurate densitometry.

Figure 6 b, c

myoblast

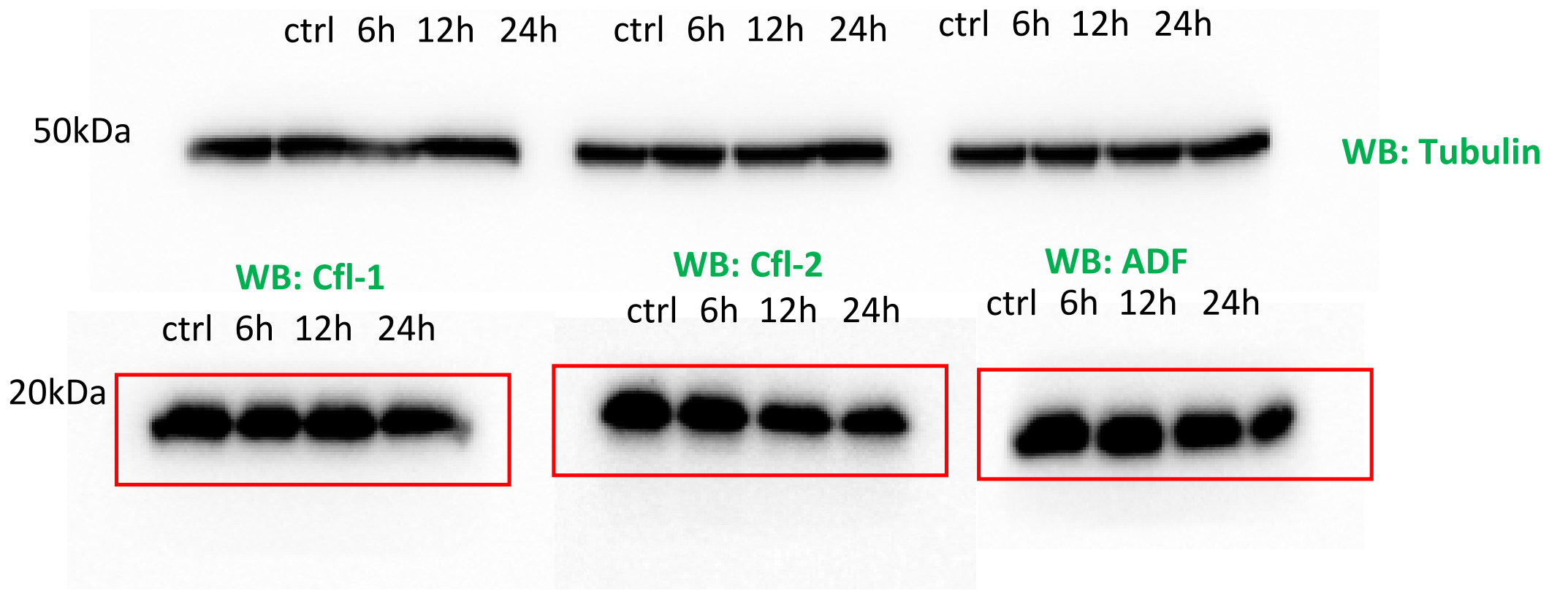

myotube

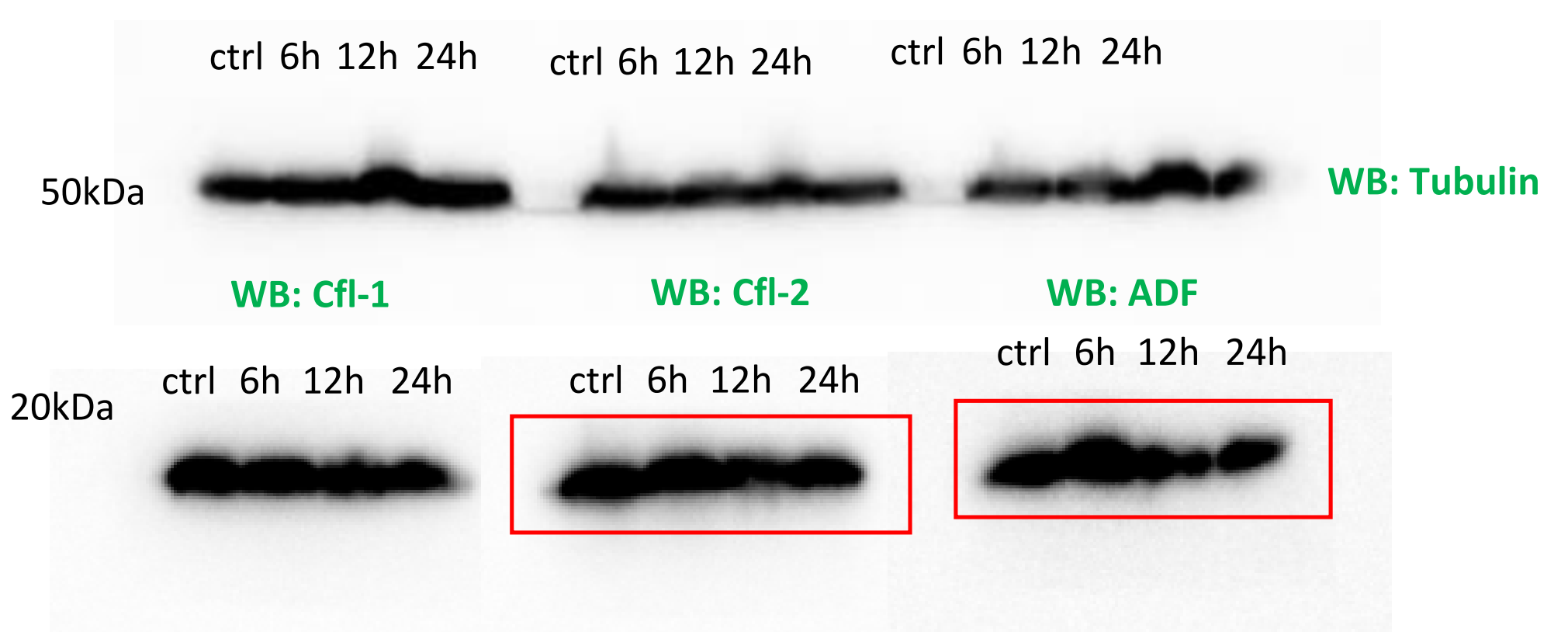

Figure 6 b, c

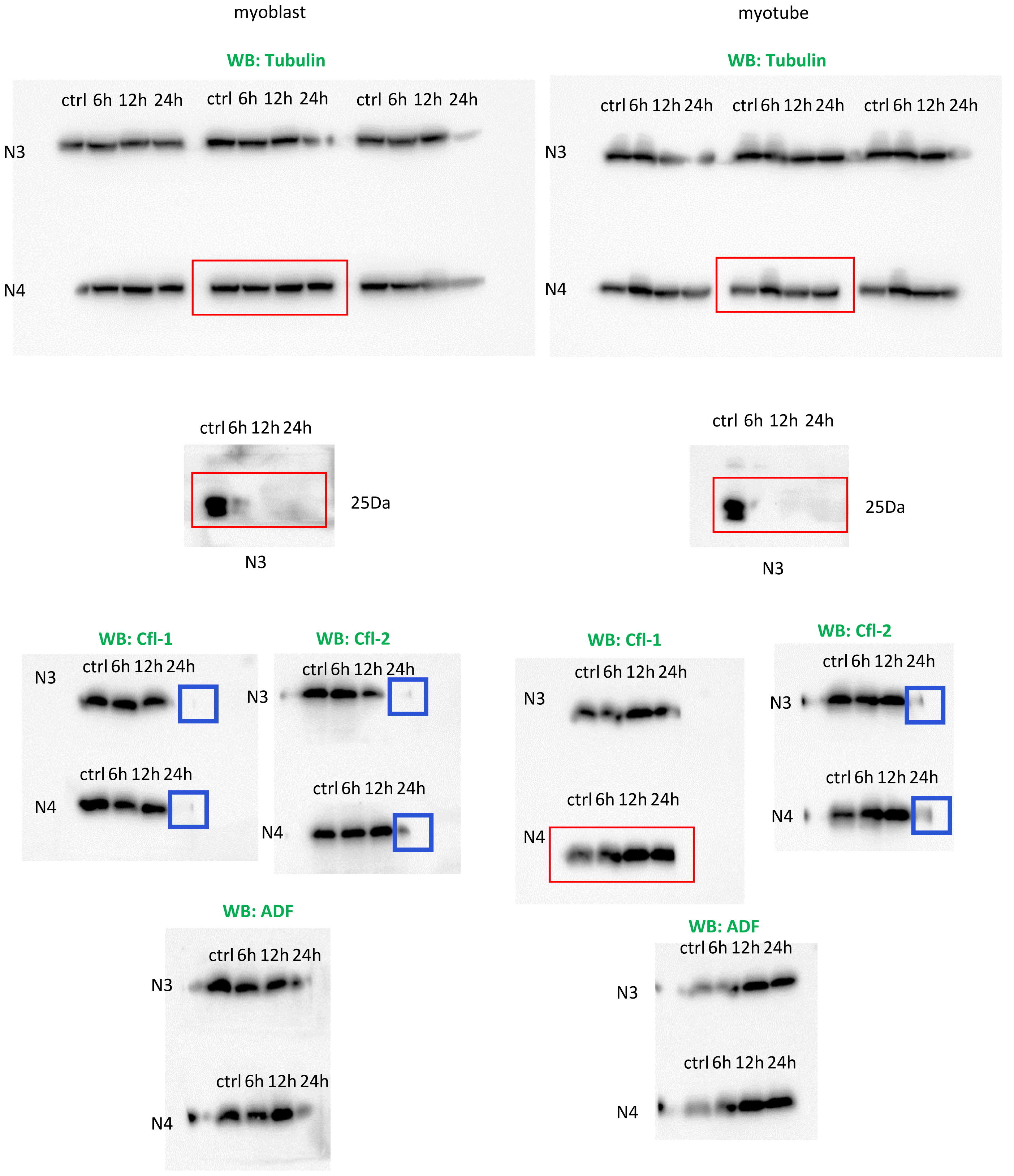

For the Western blot quantification in Figure 6, time points in the cycloheximide chase were excluded from the final analysis due to a technical loading artifact that precluded accurate densitometry.

Figure 6 b, c

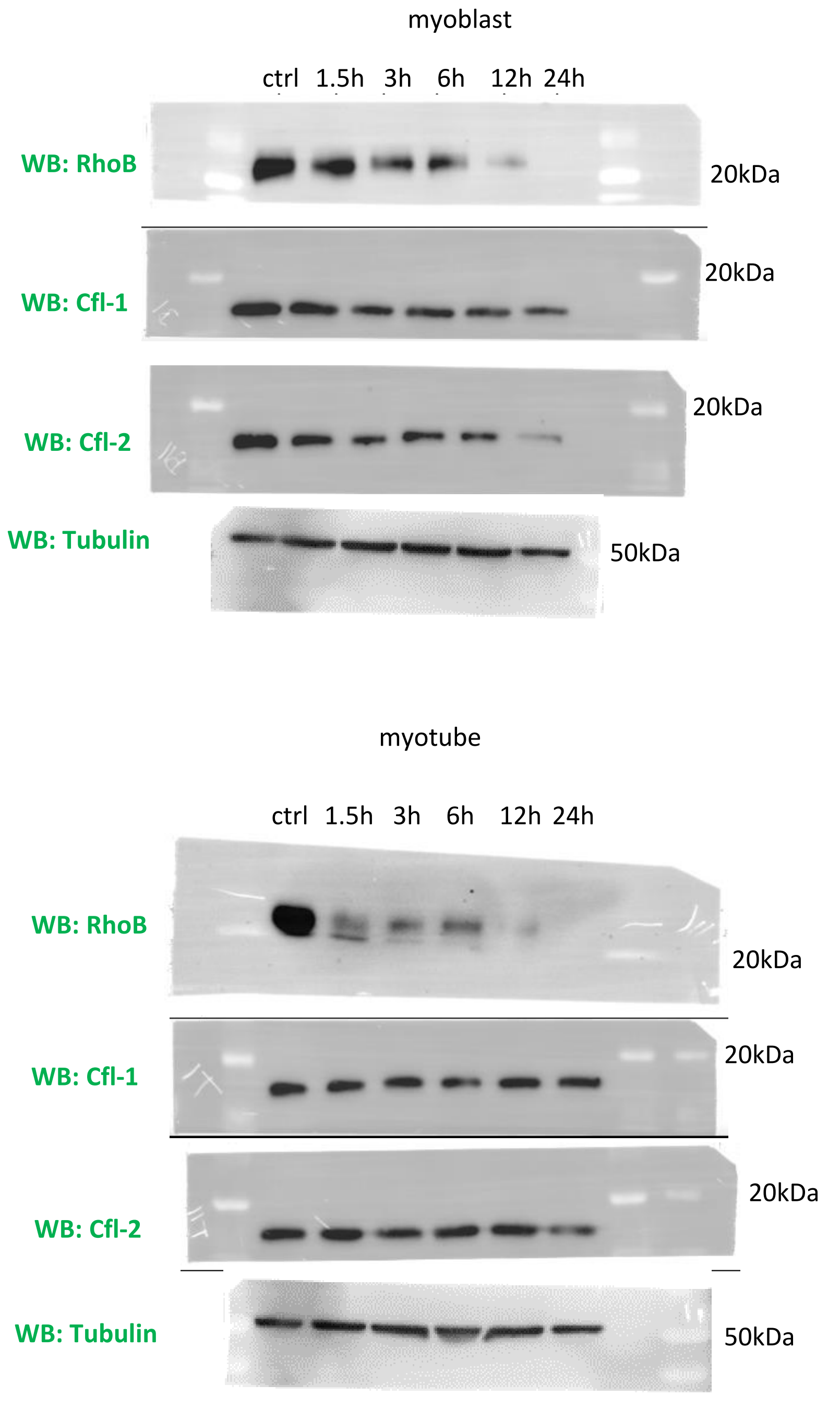
myoblast
ctrl 1.5h 3h 6h 12h 24h
WB: RhoB
20kDa
WB: Cfl-1
20kDa
WB: Cfl-2
20kDa
WB: Tubulin
50kDa
myotube
ctrl 1.5h 3h 6h 12h 24h
WB: RhoB
20kDa
WB: Cfl-1
20kDa
WB: Cfl-2
20kDa
WB: Tubulin
50kDa

Figure 7 a, b

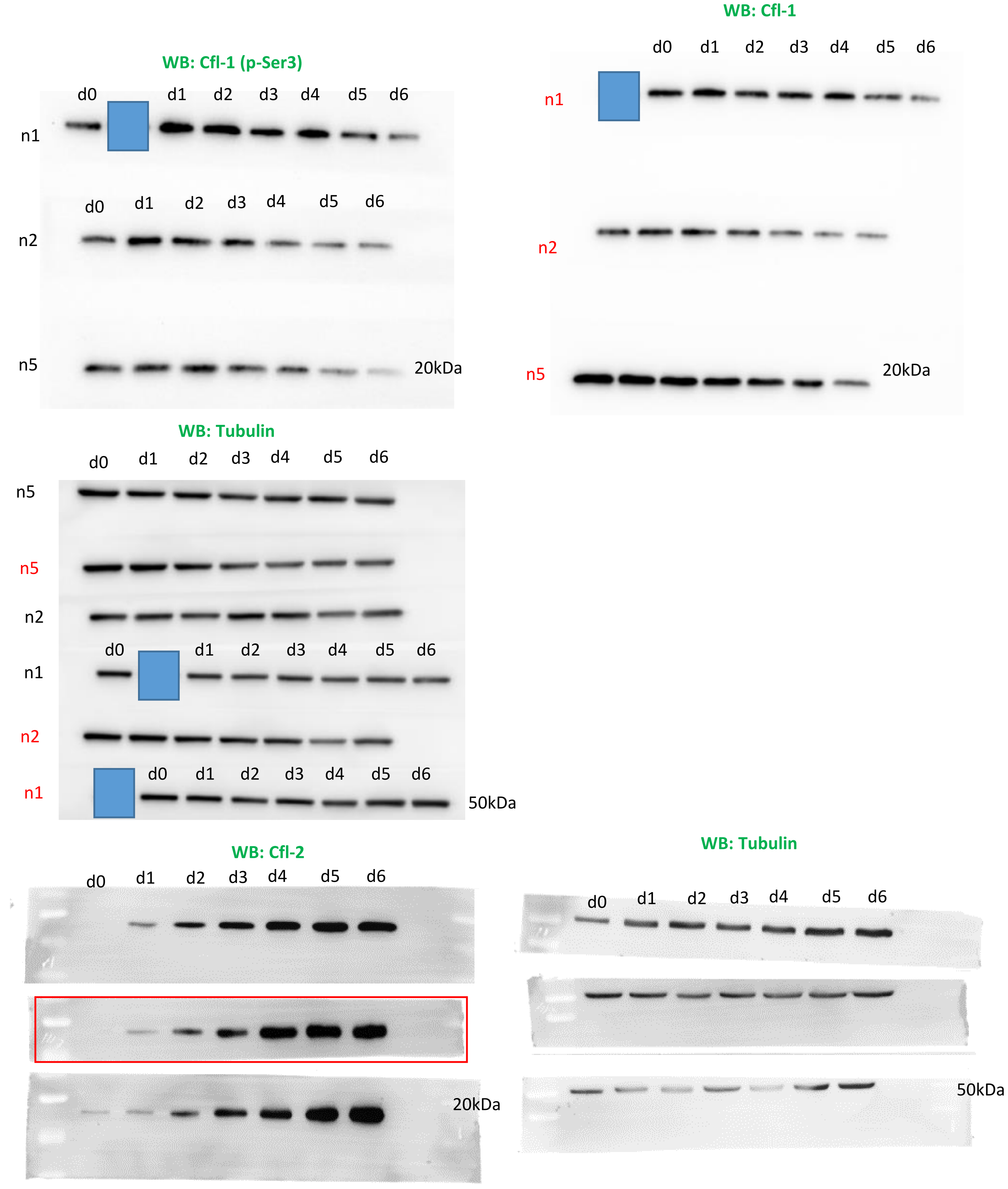

For the Western blot quantification the lane marked with an blue rectangle was excluded from quantification due to a recorded pipetting error during sample loading. An inconsistent volume was applied to this lane, resulting in an inaccurate total protein load that precluded reliable normalization. This sample was omitted from the final analysis to ensure quantitative accuracy.

Figure 7 a, b

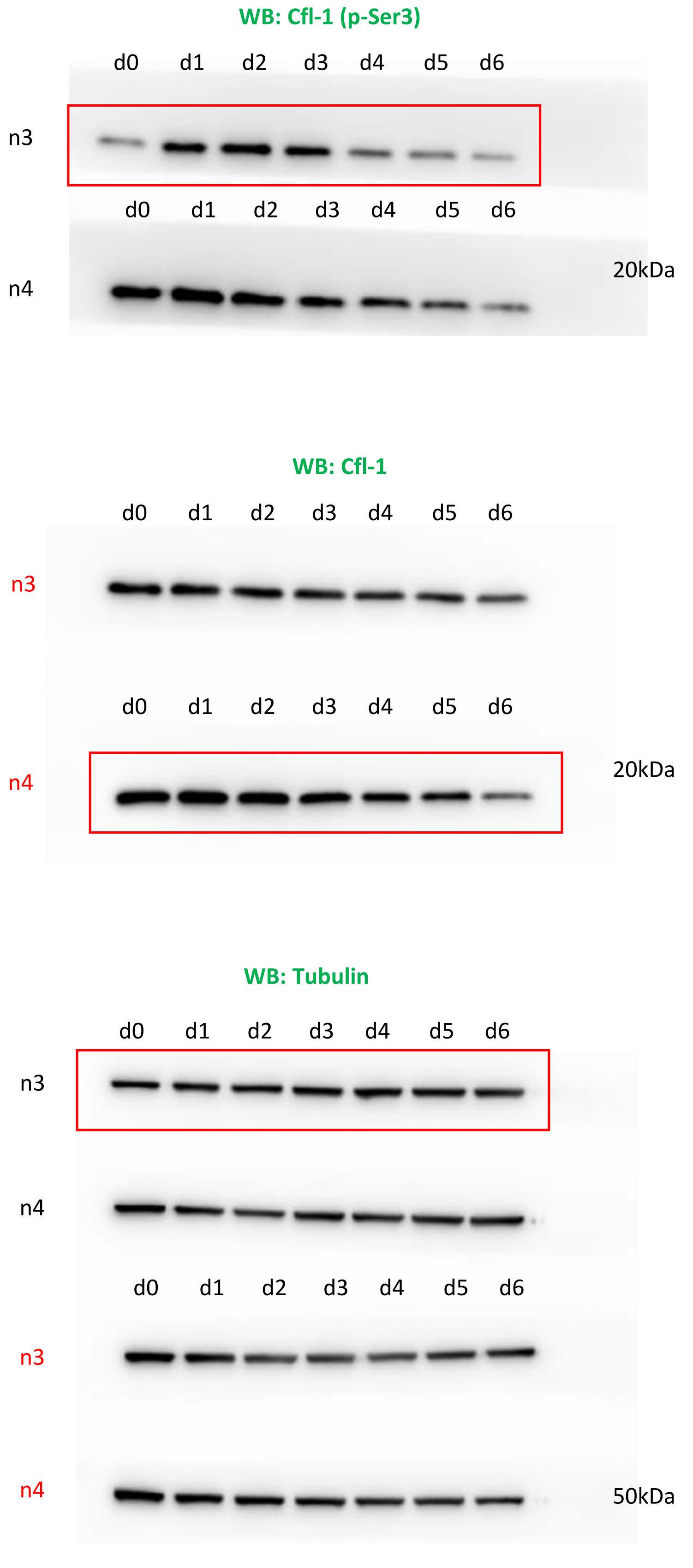
WB: Cfl-1 (p-Ser3)
d0 d1 d2 d3 d4 d5 d6
n3
d0 d1 d2 d3 d4 d5 d6
n4
20kDa
WB: Cfl-1
d0 d1 d2 d3 d4 d5 d6
n3
d0 d1 d2 d3 d4 d5 d6
n4
20kDa
WB: Tubulin
d0 d1 d2 d3 d4 d5 d6
n3
n4
d0 d1 d2 d3 d4 d5 d6
n3
n4
50kDa

Figure 7 c, d

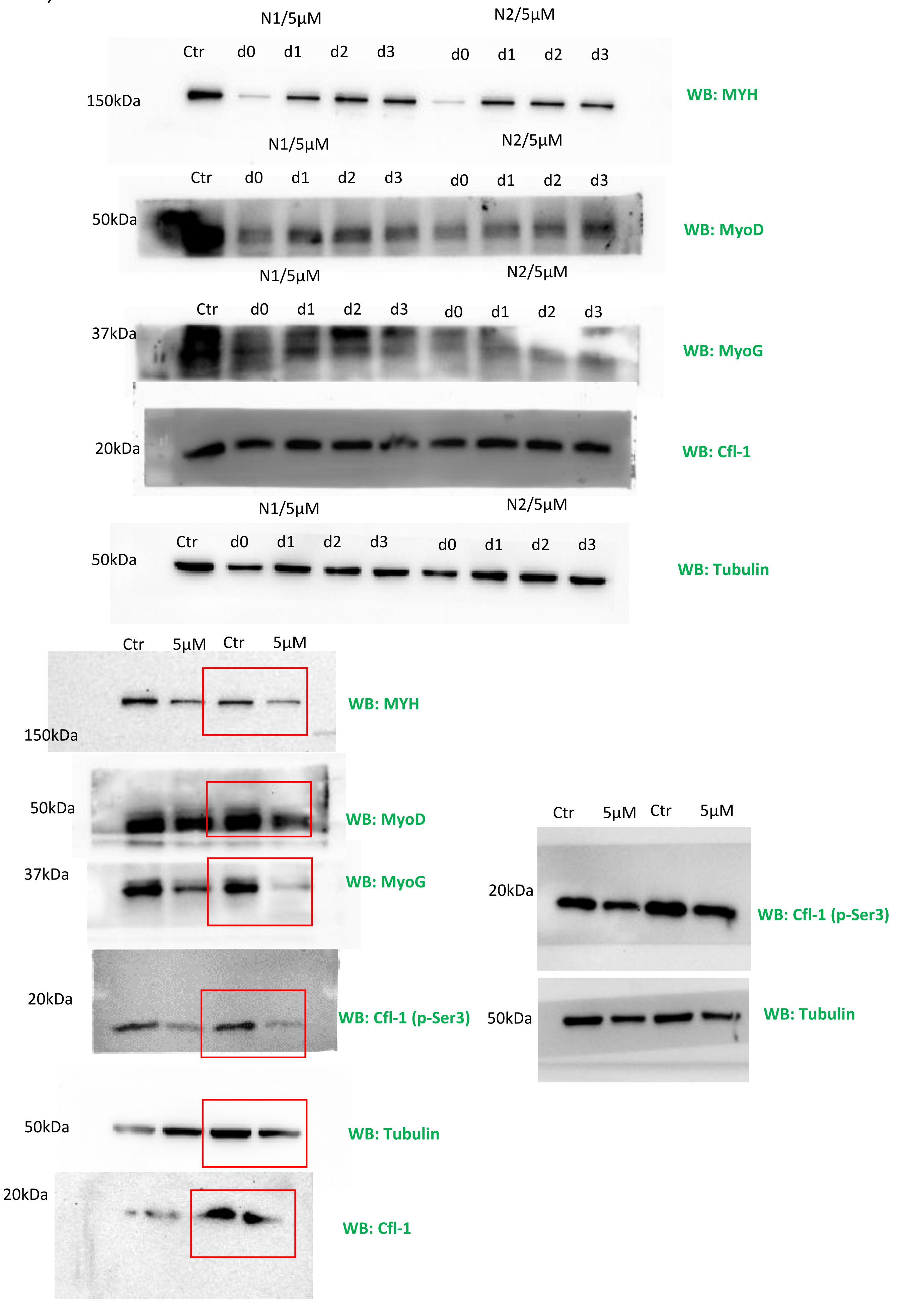
N1/5µM
N2/5µM
Ctr
d0
d1
d2
d3
d0
d1
d2
d3
150kDa
WB: MYH
50kDa
WB: MyoD
37kDa
WB: MyoG
20kDa
WB: Cfl-1
50kDa
WB: Tubulin
Ctr
5µM
Ctr
5µM
150kDa
WB: MYH
50kDa
WB: MyoD
37kDa
WB: MyoG
20kDa
WB: Cfl-1 (p-Ser3)
50kDa
WB: Tubulin
20kDa
WB: Cfl-1
Ctr
5µM
Ctr
5µM
20kDa
WB: Cfl-1 (p-Ser3)
50kDa
WB: Tubulin

Figure 7 c, d

LIMKI3 O/N do-d6 n1

WB: Cfl-1

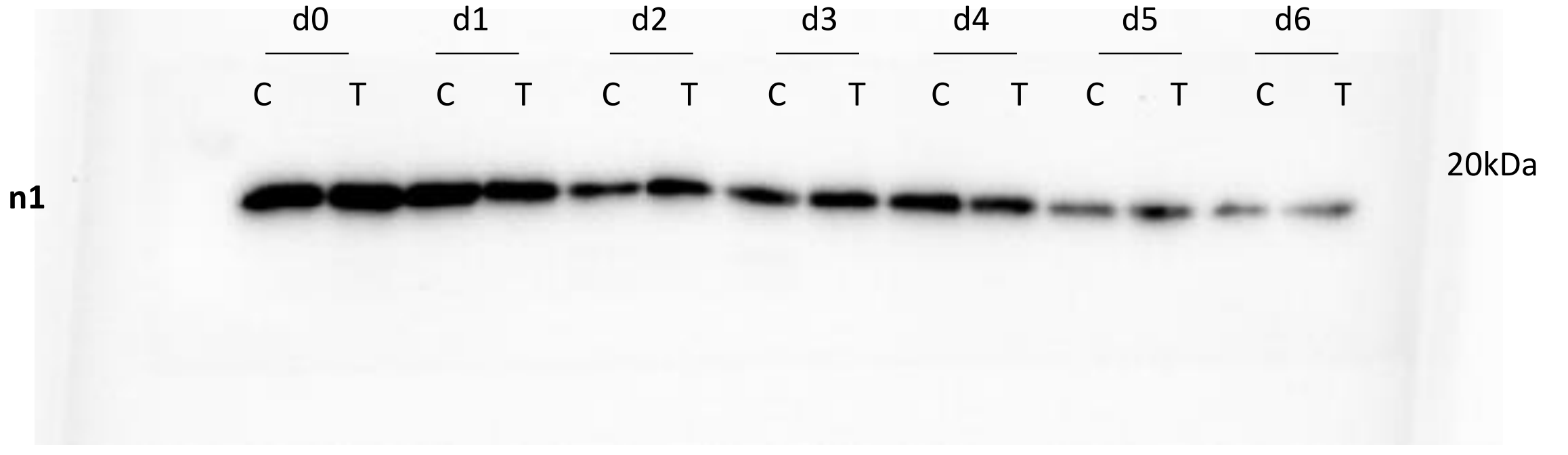

WB: MYH

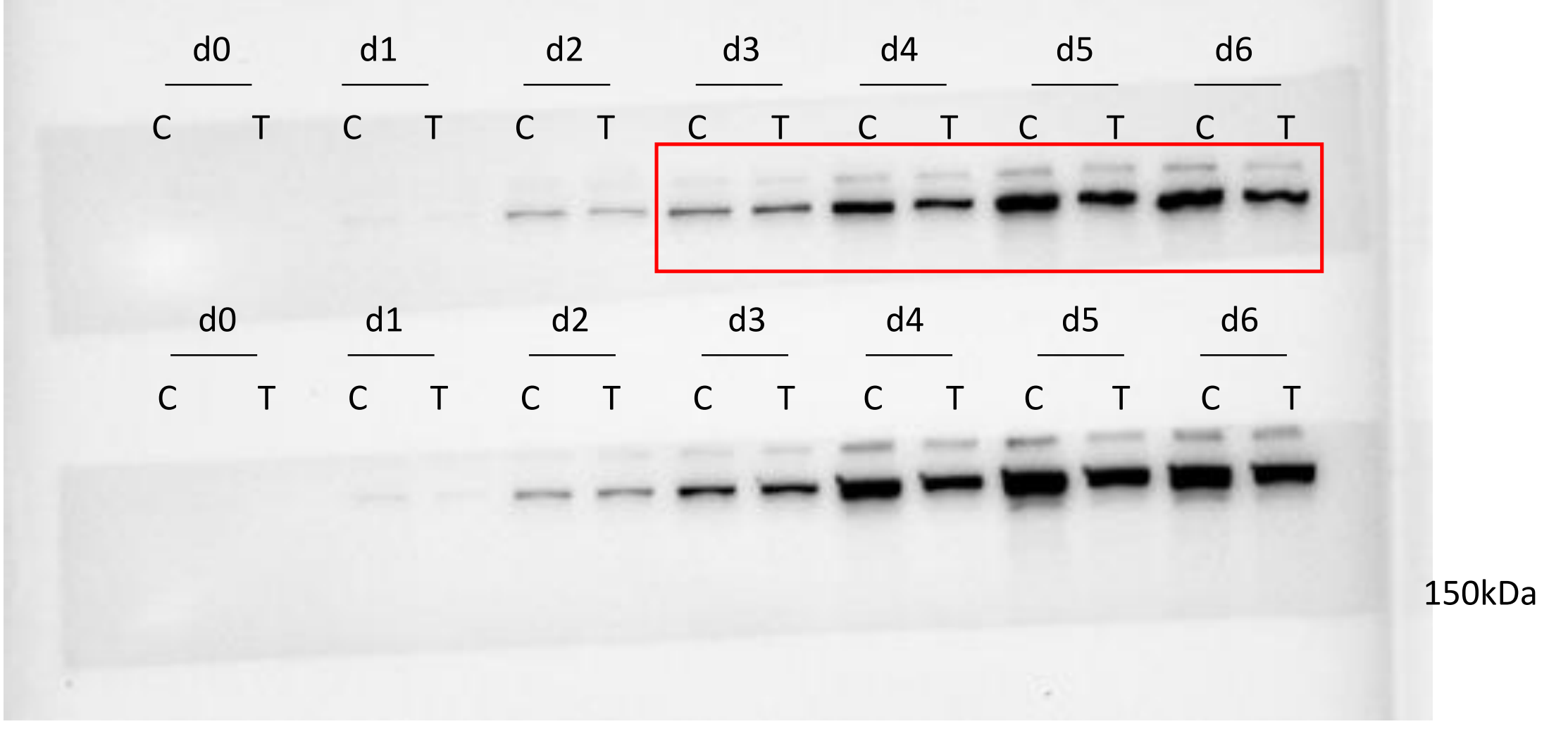

WB: Tubulin

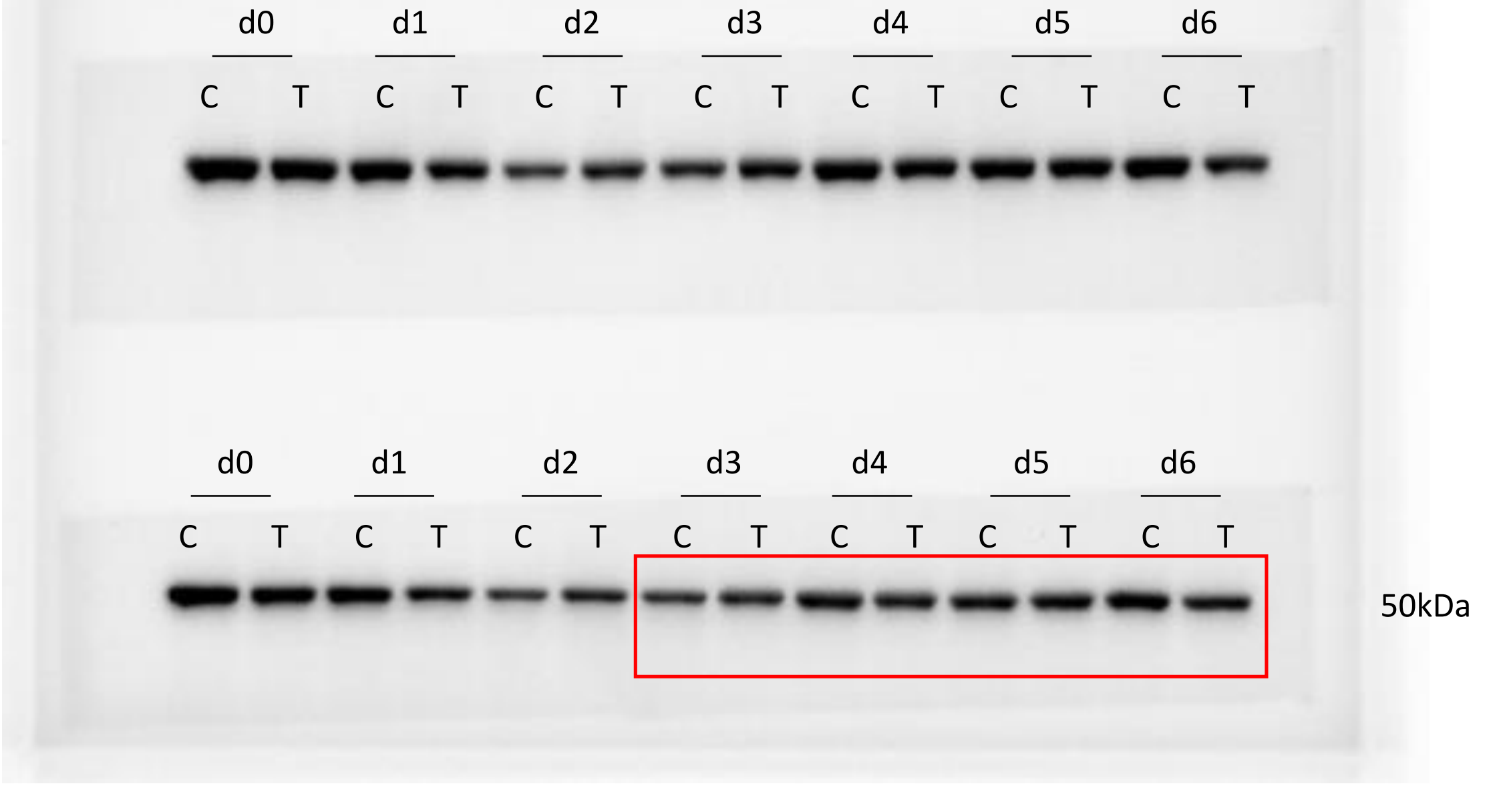

Figure 7 c, d

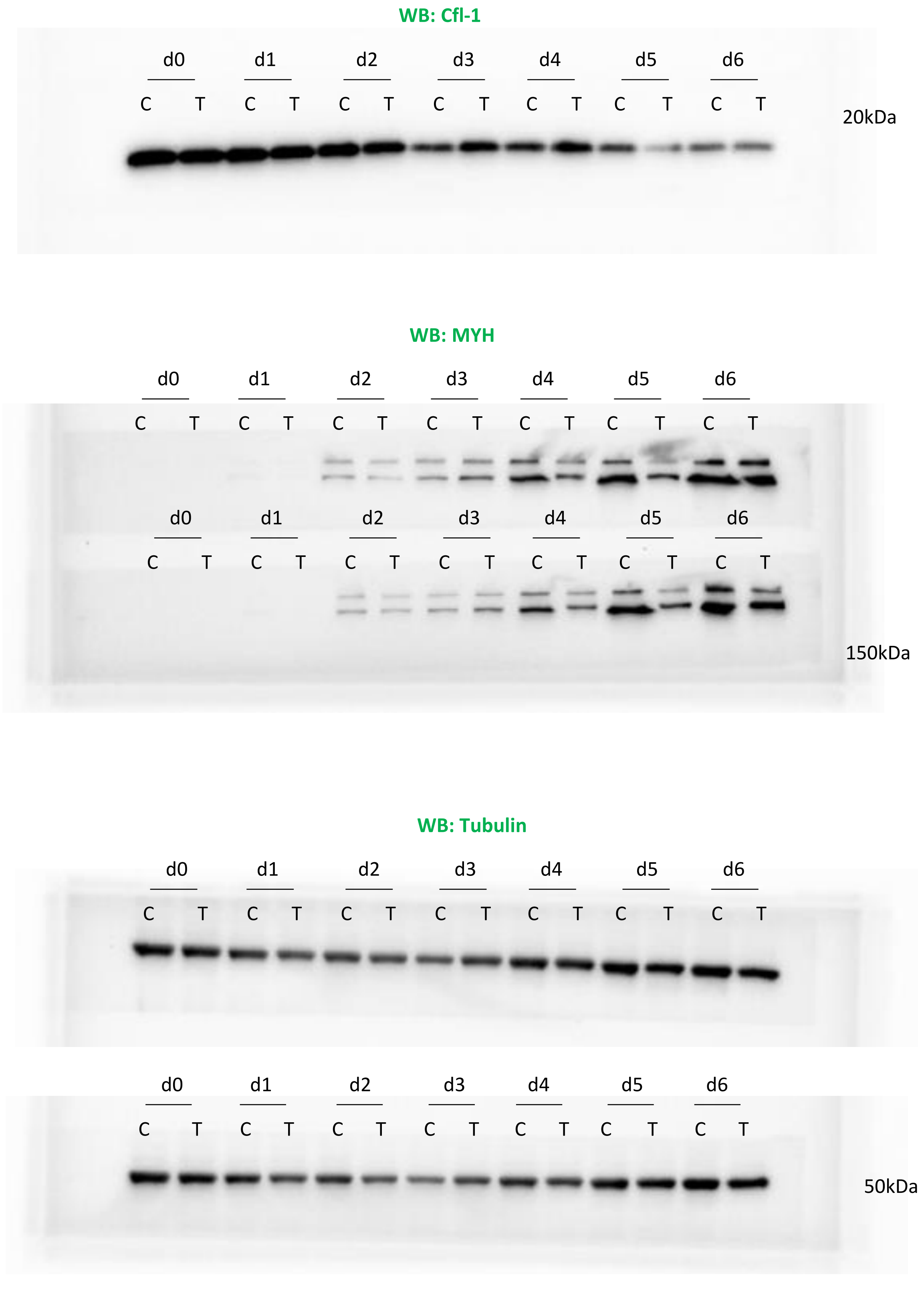
WB: Cfl-1
d0 d1 d2 d3 d4 d5 d6
C T C T C T C T C T C T C T
20kDa
WB: MYH
d0 d1 d2 d3 d4 d5 d6
C T C T C T C T C T C T C T
d0 d1 d2 d3 d4 d5 d6
C T C T C T C T C T C T C T
150kDa
WB: Tubulin
d0 d1 d2 d3 d4 d5 d6
C T C T C T C T C T C T C T
d0 d1 d2 d3 d4 d5 d6
C T C T C T C T C T C T C T
50kDa

Figure S1

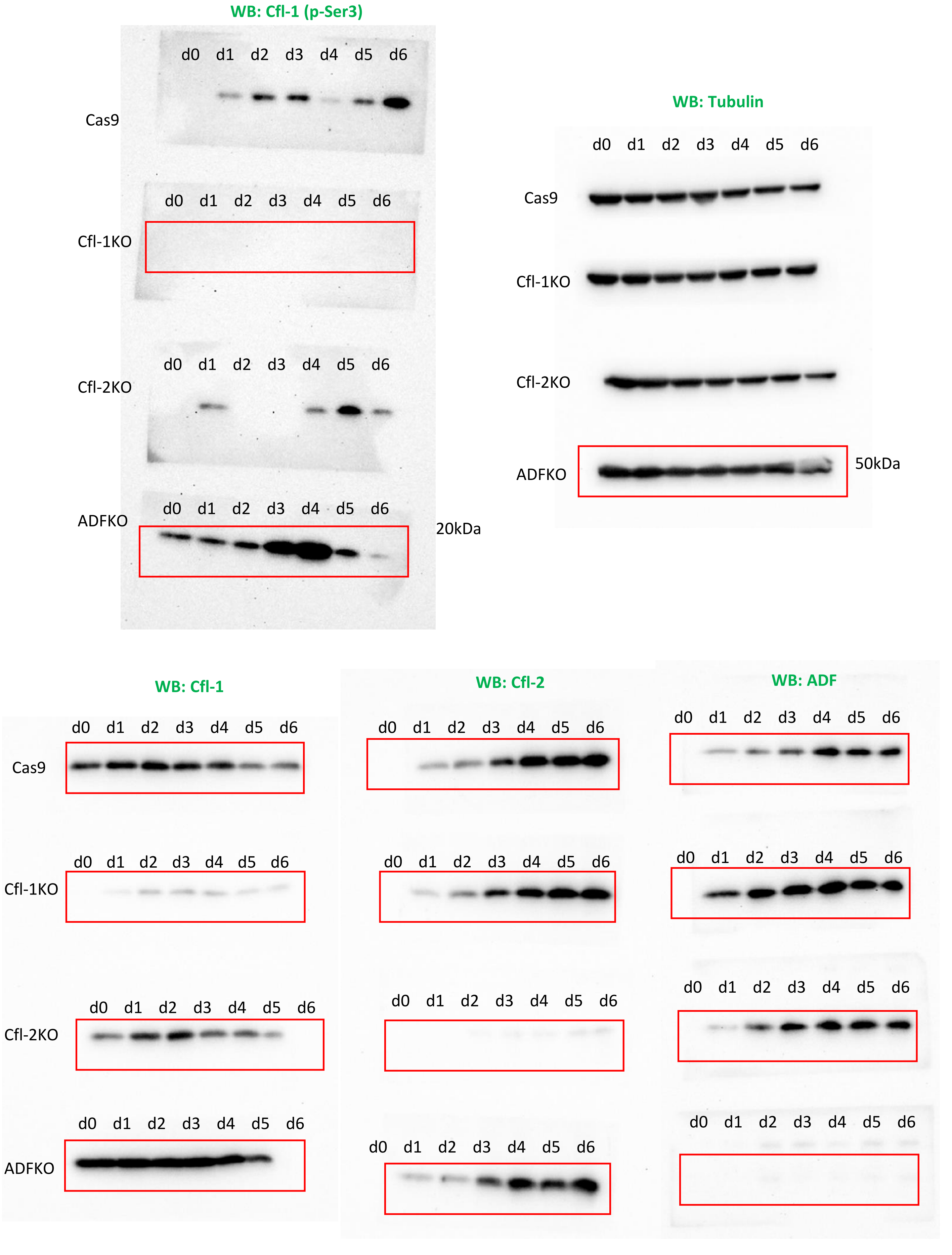
WB: Cfl-1 (p-Ser3)
d0 d1 d2 d3 d4 d5 d6
Cas9
Cfl-1KO
Cfl-2KO
ADFKO
20kDa
WB: Tubulin
d0 d1 d2 d3 d4 d5 d6
Cas9
Cfl-1KO
Cfl-2KO
ADFKO
50kDa
WB: Cfl-1
WB: Cfl-2
WB: ADF
d0 d1 d2 d3 d4 d5 d6
Cas9
Cfl-1KO
Cfl-2KO
ADFKO

Figure S4

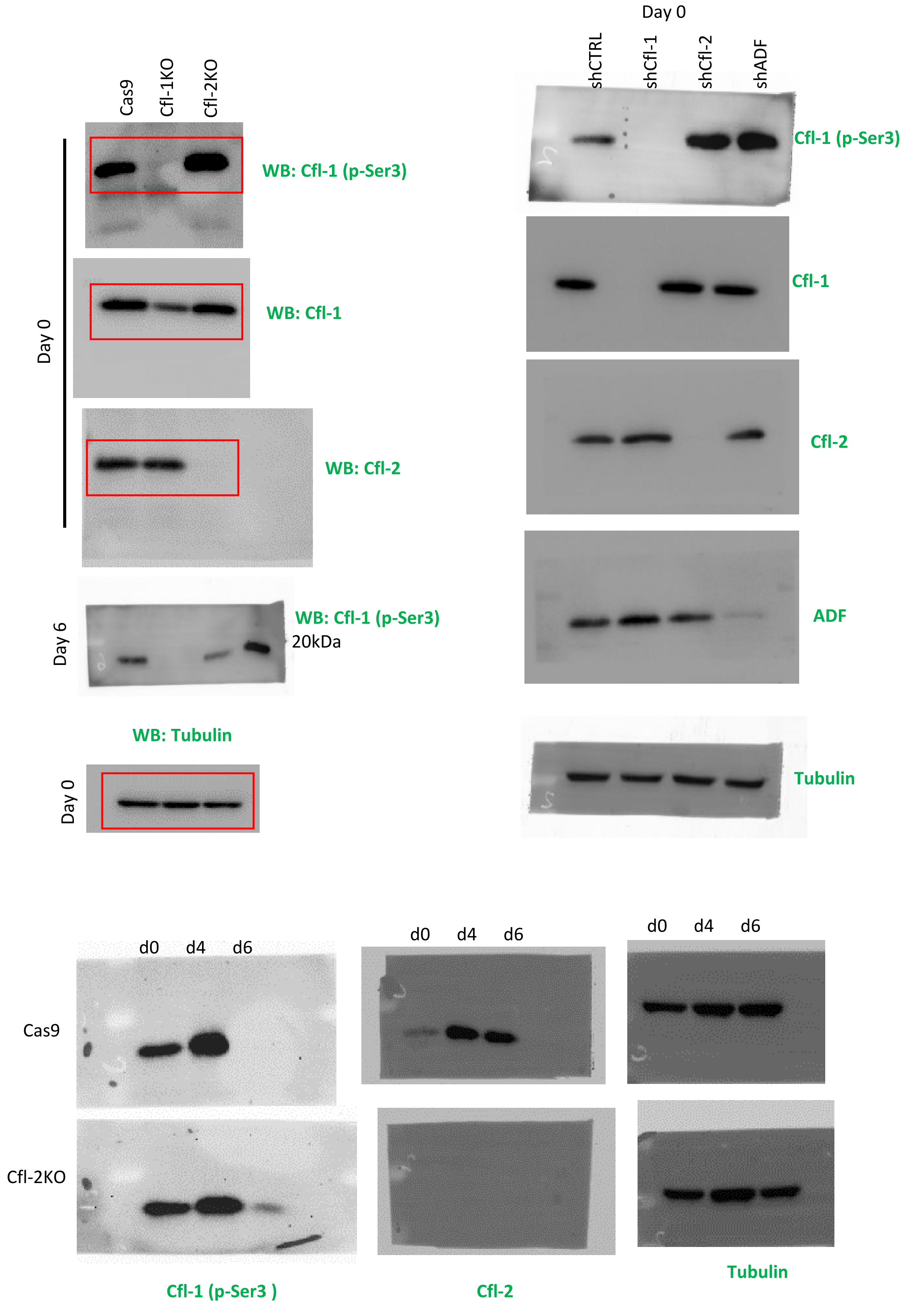

Cas9
Cfl-1KO
Cfl-2KO
Day 0
WB: Cfl-1 (p-Ser3)
WB: Cfl-1
WB: Cfl-2
Day 6
WB: Cfl-1 (p-Ser3)
20kDa
WB: Tubulin
Day 0
Day 0
shCTRL
shCfl-1
shCfl-2
shADF
Cfl-1 (p-Ser3)
Cfl-1
Cfl-2
ADF
Tubulin
d0
d4
d6
Cas9
Cfl-2KO
d0
d4
d6
d0
d4
d6
Cfl-1 (p-Ser3)
Cfl-2
Tubulin
